# Nap1L4a Cooperates with Scl/Klf1 to Recruit H2A.Z in Mediating Interactions Among *Cis*‐Regulatory Elements and Transcription Required for Primitive Erythropoiesis in Zebrafish

**DOI:** 10.1002/advs.202513762

**Published:** 2025-12-12

**Authors:** JiaHao Shi, FuMing Lai, Zheng Shen, XiaoYan Zhang, HanFei Wang, WenYe Liu, YunLong Wang, KuanYu Li, GuoLiang Li, YaPing Fang, Jing‐Xia Liu

**Affiliations:** ^1^ College of Fisheries, Key Laboratory of Freshwater Animal Breeding, Ministry of Agriculture Huazhong Agricultural University Wuhan 430070 China; ^2^ College of Informatics, Agricultural Bioinformatics Key laboratory of Hubei Province, Hubei Engineering Technology Research Center of Agricultural Big Data Huazhong Agricultural University Wuhan 430070 China; ^3^ Henan Key Laboratory of Medical Tissue Regeneration Xinxiang Medical University Xinxiang 453003 China; ^4^ State Key Laboratory of Pharmaceutical Biotechnology, Jiangsu Key Laboratory of Molecular Medicine, Medical School Nanjing University Nanjing 210093 China

**Keywords:** histone epigenomics, Kdm6b/Kmt2c, Nap1l4a, primary erythropoiesis, Scl/ Klf1, WNT/β‐Catenin

## Abstract

The chromatin remodeler nucleosome assembly protein 1‐like 4 (Nap1L4) is highly expressed in megakaryocyte‐erythroid progenitors (MEPs) and erythroid cells. Mutations, deletions, and aberrant expressions of Nap1L4 are observed in diseases such as acute myeloid leukemia (AML). However, the roles of Nap1l4a in erythropoiesis and related diseases, as well as the underlying mechanisms, remain unknown. Here, it is demonstrated that zebrafish *nap1l4a* homozygous mutants (*nap1l4a* ^−/−^) are more sensitive to hypoxia stress during the early embryonic stage and exhibit impaired primitive erythropoiesis. Mechanistically, zebrafish Nap1l4a interacts with the erythropoietic transcription factors (TFs) Scl and Klf1, and recruits the histone variant H2A.Z. This interaction remodels the cis‐regulatory element (CRE) landscape and promotes nascent RNA transcription of erythropoietic genes. Meanwhile, Nap1l4a deficiency impairs chromatin accessibility at the epigenetic regulators *kdm6b* and *kmt2c*. This results in expanded H3K27me3 and diminished H3K4me1 in erythrocytes, leading to altered histone landscapes at erythropoiesis TF loci and reduced TF expression. Moreover, Nap1l4a regulates primitive erythropoiesis by transcriptionally and epigenetically modulating the canonical WNT/β‐Catenin pathway. Together, the findings reveal a lineage‐selective transcription, with histone epigenomics‐dependent role for *nap1l4a* in vertebrate primitive erythropoiesis. These findings highlight potential mechanisms underlying human blood disorders and hypoxia responses associated with Nap1l4a deficiency.

## Graphical abstract

Nap1l4a is required in erythropoiesis and hypoxia responses via physical interaction with Klf1 and Scl to recruit the histone variant H2A.Z. This facilitates its associated *cis*‐regulatory element (CRE) remodeling and the consequent chromatin assembly, and activates the transcription of erythroid lineage‐specific genes.

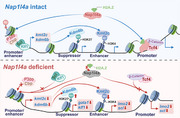

## 1 Introduction

Hematopoiesis in zebrafish consists of two major waves: the primitive wave and the definitive wave.^[^ ^1^ ^]^ The primitive wave generates erythrocytes and macrophages required for early embryonic development. In contrast, the definitive wave gives rise to hematopoietic stem and progenitor cells (HSPCs), which maintain the production of all blood lineages throughout the vertebrate lifespan.^[^ ^2^ ^]^ Primitive erythrocytes supply oxygen to the embryo, supporting its rapid growth and development.^[^ ^3^ ^]^ Failure to initiate the first wave of erythropoiesis (primitive erythropoiesis) or dysregulated of erythroid differentiation can lead to severe anemia, markedly reduced survival rates,^[^ ^4^ ^]^ and occasional lethality in embryos and larvae.^[^ ^5^ ^]^

A large‐scale in vivo reverse genetic screen targeting zebrafish homologs of 425 human chromatin factors using antisense oligonucleotide morpholinos (MOs) revealed that *nap1l4a* knockdown reduces the expression of both primitive and definitive hematopoietic genes, including *β‐globin* and *runx1*.^[^ ^6^ ^]^ We recently reported that *nap1l4a* may act as a downstream mediator of *epc1/2* in regulating HSPC development.^[^ ^7^ ^]^ These findings suggest that *NAP1L4* may play important roles in hematopoiesis and hematopoietic disorders. NAP proteins are an evolutionarily conserved family of histone chaperones that transport H2A–H2B dimers from the cytoplasm to the nucleus.^[^ ^8^ ^]^ They also regulate chromatin assembly and disassemble dynamics,^[^ ^9^ ^]^ thereby influencing transcription.^[^ ^10^ ^]^ *NAP1L4* is highly expressed in megakaryocyte‐erythroid progenitors (MEPs) and erythroid cells (BloodSpot), and shows mutations, deletions, and aberrant expression in cancers such as acute myeloid leukemia (https://www.cbioportal.org/). One study reported that depletion of *Nap1L* in *Xenopus* embryos leads to transcriptional downregulation of *α‐globin* and hematopoietic precursor genes *scl* and *runx1*.^[^ ^11^ ^]^ However, the roles of *Nap1l4a* in hematopoiesis, particularly in erythropoiesis, remain largely unknown in vertebrates.

The intricate process of erythropoiesis is orchestrated by the coordinated actions of transcription factors (TFs), epigenetic regulators, and the dynamically changing epigenomes, which together fine‐tune the activity of *cis*‐regulatory elements (CREs), including promoters, silencers, and enhancers. Erythroid lineage TFs, including *Tal1*/*Scl*, *Lmo2*, *Gata1*, *Klf1*, and *Klf6*, are suggested to form a recursive gene‐regulatory circuit that controls erythropoiesis.^[^ ^12^, ^13^ ^]^ These TFs act at CREs to recruit epigenetic regulators, including the H3K4me1 methyltransferase MLL3 (Kmt2C),^[^ ^14^ ^]^ and the H3K27ac acetyltransferases CBP and p300.^[^ ^15^ ^]^ This recruitment promotes dynamic chromatin accessibility at erythroid lineage genes and activates their transcription. Meanwhile, repressive markers at CREs, such as H3K27me3, which is removed by demethylases such as Kdm6b,^[^ ^16^ ^]^ inhibit the transcription of erythropoiesis genes.^[^ ^17^, ^18^ ^]^ However, whether and how Kmt2C and Kdm6b function in erythropoiesis, and whether they mediate the roles of Nap1l4a in erythropoiesis, remain unknown.

To address these questions, we generated *nap1l4a* homozygous mutants (*nap1l4a* ^−/−^), with a 5 bp deletion in exon 4. These mutants were more sensitive to hypoxic stress during early embryonic stages and exhibited impaired primitive erythropoiesis. We then characterized the chromatin architecture of erythropoiesis‐related genes and examined histone modifications at their CREs. In summary, our results showed that Nap1l4a functionally interacts with the erythrocyte‐specific TFs Klf1 and Scl, and recruits the histone variant H2A.Z. This interaction facilitates chromatin assembly and activates the transcription of erythroid lineage‐specific genes. Meanwhile, *nap1l4a* modulates chromatin assembly and the transcription of the epigenetic modulators *kdm6bb* and *kmt2ca*, thereby maintaining normal histone epigenomic sates (H3K27me3, H3K4me1, and H3K27ac) at the CREs of erythropoiesis genes. Moreover, *nap1l4a* deficiency results in altered epigenomic states at CREs and chromatin inaccessibility of WNT pathway genes, thereby impairing their transcription. This may represent an additional mechanism contributing to defective primitive erythropoiesis in *nap1l4a^−/−^* embryos.

## 2 Results

### 2.1 Nap14a Positively Regulates Primitive Erythropoiesis

MOs mediated knockdown of *nap14a* in zebrafish leads to reduced transcription of genes involved in both primitive and definitive hematopoiesis.^[^ ^6^ ^]^ Consistently, decreased *nap14a* expression in the intermediate cell mass (ICM) at 28 hpf (hours post fertilization) (Figure S1A,B, Supporting Information) is also observed in zebrafish mutants lacking *epc1/2* genes required for definitive hematopoiesis,^[^ ^7^ ^]^ further supporting a potential role for *nap14a* in hematopoietic regulation.

**Figure 1:**
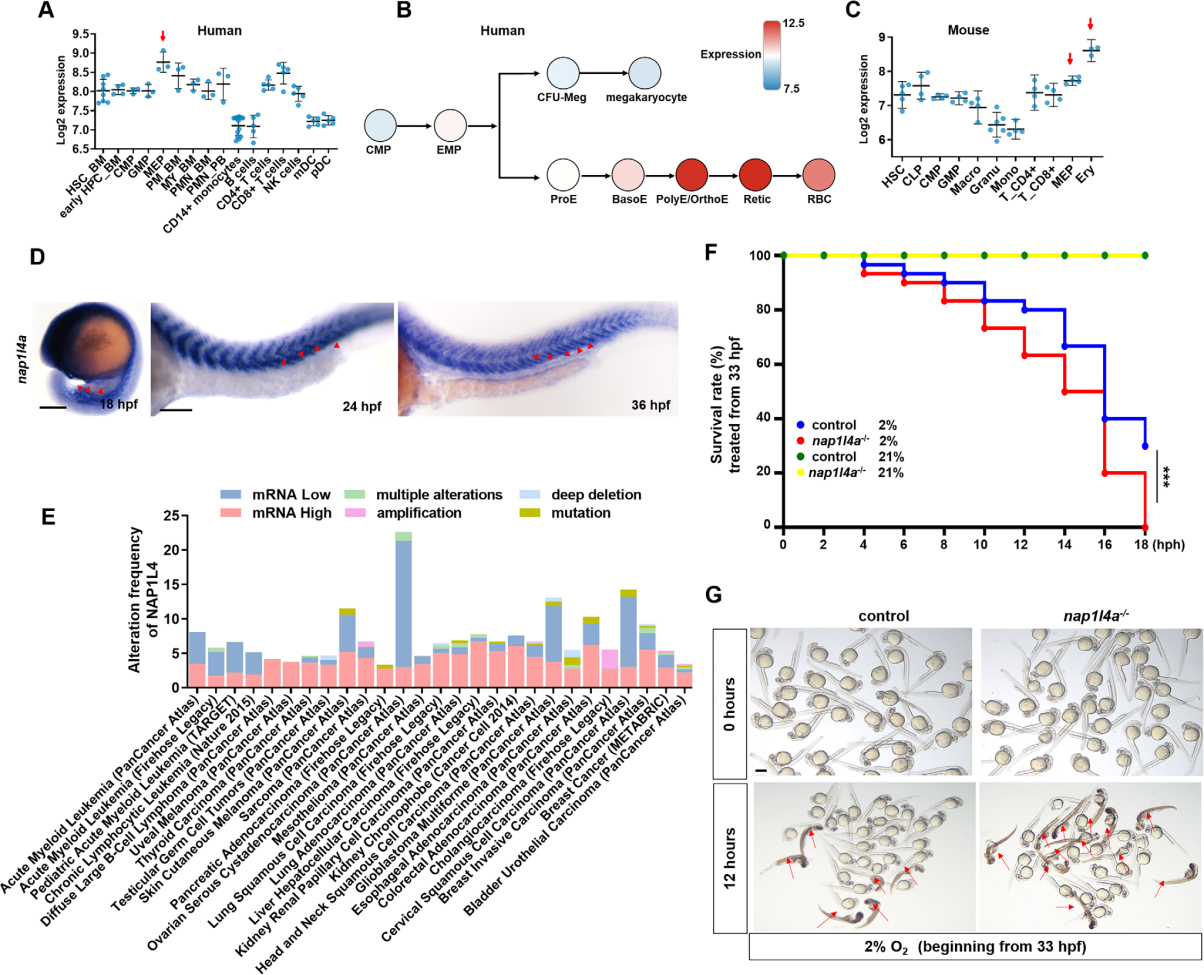
Transcriptional expression and mutation of *Nap1l4a* in human diseases, mouse, and zebrafish. A−C) *Nap1l4* expression in megakaryocyte‐erythroid progenitor cells (MEPs), erythrocytes and during erythroid differentiation in (A,B) human and (C) mouse. Enriched expression of *Nap1l4* in EMPs and erythrocytes in both human and mouse are indicated by red arrows. D) Distribution of *Nap1l4a* transcripts during zebrafish embryogenesis, predominantly in the ICM at 18 and 24 hpf, and in aorta‐gonad‐mesonephros (AGM) region at 36 hpf. ICM and AGM regions are indicated by red arrowheads. E) Abnormal expression and mutations of *Nap1l4* have been observed in cancers and myeloid cell related disease. F) Survival curves of *nap1l4a* ^−/−^and wild‐type (WT) control embryos exposed to hypoxia (2% O_2_) beginning at 33 hpf for 18h. G) Representative images of *nap1l4a* ^−/−^and WT control embryos exposed to hypoxia (2% O_2_) beginning at 33 hpf for 12h with dead larvae indicated by red arrows. D, lateral view, anterior to the left. Each experiment was repeated at least three times, and a representative result is shown. HSC_BM: Hematopoietic stem cells from bone marrow, early HPC_BM: Hematopoietic progenitor cells from bone marrow; PM_BM: Promyelocyte from bone marrow, MY_BM: Myelocyte from bone marrow, PMN_BM: Polymorphonuclear cells from bone marrow, PMN_PB: Polymorphonuclear cells from peripheral blood, NK cells: CD56^+^ natural killer cells, mDC: CD11c^+^ myeloid dendritic cells, CLP: Common lymphoid progenitor cells, Macro: Bone Marrow Macrophages, Granu: Granulocytes, Mono: Monocytes. Data are presented as mean ± SD. Hpf, hours post fertilization. *P < 0.05. Scale bar, 2 mm (G).

Here, we found that *Nap1l4* is highly expressed in MEPs and erythrocytes, as well as during erythroid differentiation, in both human and mice based on BloodSpot analysis^[^ ^19^ ^]^ (**Figure** **1**A–C). In zebrafish embryos, *nap14a* transcripts were predominantly detected in the ICM at 18 hpf and 24 hpf, and in the aorta‐gonad‐mesonephros (AGM) region at 36 hpf (Figure 1D), corresponding to the sites of primitive and definitive erythropoiesis occur, respectively.^[^ ^20^ ^]^ We also detected abundant maternal transcripts and translational Nap1l4a in early zebrafish embryos (Figure S1E,F, Supporting Information).

*NAP1L4* exhibits mutations, deletions, or aberrant expression in various cancers and is predominantly up‐ or down‐regulated at the transcriptional level in myeloid cell related disease, such as acute myeloid leukemia (Figure 1E). *Nap1l4a^−/−^* (Figure S1C,D, Supporting Information) mutants showed significantly (*P* < 0.01) higher mortality than WT siblings upon hypoxic stress at 33 hpf (Figures 1F,G; S1I, Supporting Information). Nevertheless, these mutants appeared morphologically normal (Figure S1G, Supporting Information), and could develop into fertile adults.

*Nap1l1* and *Nap1l4* are ubiquitously expressed, whereas *NAP1L2*, *NAP1L3* and *NAP1L5* are predominantly expressed in neurons.^[^ ^21^, ^22^ ^]^ In this study, the transcriptional levels of *nap1l1* and *nap1l4b* remained unchanged in *nap1l4a^−/−^* mutants at 24 hpf (Figure S1H, Supporting Information), suggesting that loss of *nap1l4a* did not trigger genetic compensation responses (GCRs) by other family members.^[^ ^23^ ^]^ Furthermore, flow‐cytometry analysis of hematopoietic cells in adult whole kidney marrow (WKM) revealed no significant differences in erythrocytes or precursor cells between *nap1l4a^−/−^* mutants and WT controls at 3 month post fertilization (mpf) (Figure S1J, Supporting Information), indicating that *nap1l4a* deficiency had no substantial effect on red blood cells in adult zebrafish.

A significant reduction in HIF‐1α (hypoxia inducible factor 1α) protein level was observed in *nap1l4a* ^−/−^ larvae at 14 hph (hours post hypoxia, and hypoxic stress beginning at 33 hpf), compared with WT control (Figure S2A, Supporting Information). In addition, the transcriptional levels of hypoxia‐responsive genes, including *hif1αb*, *hif2αb*, *hif3α*, *cited2*, *pail1*, and *idha*, were markedly reduced in mutants both before and after hypoxic stress (Figure S2B, Supporting Information). These findings indicate that *nap1l4a* deficiency impairs the expression of hypoxia‐responsive genes and proteins, which may contribute to the heightened sensitivity of *nap1l4a* ^−/−^ embryos to hypoxia stress.

**Figure 2:**
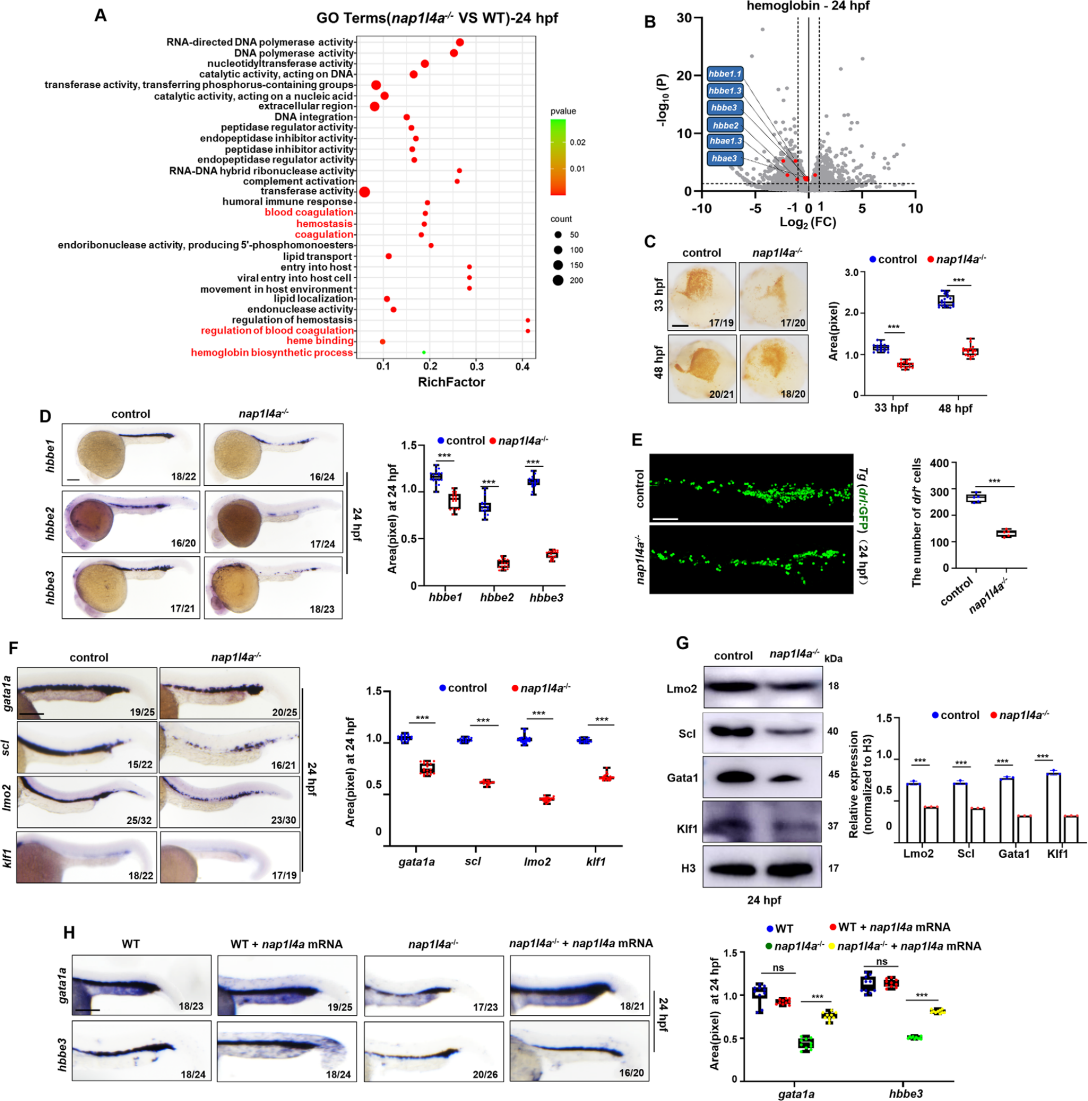
*Nap1l4a* deficiency leads to significantly reduced transcriptional and translational levels of erythropoiesis transcription factors (TFs) and hemoglobin genes. A) Bubble plot showing GO enrichment of differentially expressed genes (DEGs) in *nap1l4a* ^−/−^ versus (versus) the WT control embryos at 24 hpf. 2 biological repeats. B) Volcano plot of hemoglobin related DEGs between *nap1l4a* ^−/−^ and WT control embryos at 24 hpf. C) O‐dianisidine staining of erythrocytes at 36 and 48 hpf in *nap1l4a* ^−/−^ and WT control embryos, with box plots showing statistical analysis of O‐dianisidine staining results. D) WISH analysis of embryonic hemoglobin genes (*hbbe1*/*hbbe2*/*hbbe3*) in *nap1l4a* ^−/−^ and WT control embryos at 24 hpf, with box and whisker plots showing relative transcriptional levels. E) Representative images of *Tg* (*drl*: GFP) embryos in *nap1l4a* ^−/−^ and WT genetic background at 24 hpf, with box and whisker plots showing the number of *drl* ^+^ cells. F) WISH analysis of erythroid TFs (*gata1a*, *scl*, *lmo2*, and *klf1*) in *nap1l4a* ^−/−^ and WT control embryos at 24 hpf, with box and whisker plots showing the relative transcriptional levels. G) Protein levels of Lmo2, Scl, Gata1, and Klf1 in *nap1l4a* ^−/−^ and WT control embryos at 24 hpf, with graphs showing quantification. H) WISH analysis of *gata1a* and *hbbe3* expression in *nap1l4a* ^−/−^ and WT control embryos and in embryos rescued with full‐length *nap1l4a* mRNA at 24 hpf, with box and whisker plots showing the relative transcriptional levels. C, ventral view, anterior to the left. D, E, F, H, lateral view, anterior to the left. Each experiment was repeated at least three times, and a representative result is shown. ImageJ was used for quantifying the WISH, WB, and fluorescence signals in different groups. Data were analyzed by *t* test using GraphPad Prism 8.0. Data are presented as mean ± SD. ***P < 0.001, ns, not significant. Scale bars: 75 µm in (C–F,H).

Erythrocytes are essential for oxygen delivery during embryo development.^[^ ^3^, ^24^ ^]^ We analyzed the transcriptomic profiles of *nap1l4a* ^−/−^ embryo at 24 hpf (Table S1, Supporting Information). Gene Ontology (GO) terms related to hemoglobin (Table S2, Supporting Information) were significantly enriched among the differentially expressed genes (DEGs), and genes involved in hemoglobin and heme binding were markedly down‐regulated in the *nap1l4a* ^−/−^ mutants (**Figure** **2**A,B). GO terms associated with skeletal muscle thin filament assembly were enriched among the down‐regulated DEGs, whereas GO terms related to peripheral nervous system neuron differentiation were enriched among the up‐regulated DEGs (Figure S3A, Supporting Information). These findings suggest that, despite its ubiquitous expression during embryogenesis, Nap1l4a deficiency does not cause gross disturbance in organogenesis.

Significantly reduced hemoglobin content was observed in *nap1l4a* ^−/−^ embryos at both 33 hpf and 48 hpf (Figure 2C). The transcription levels of the embryonic globin genes *hbbe1*, *hbbe2*, and *hbbe3* were markedly decreased in the *nap1l4a* ^−/−^ embryos at 14, 24, and 33 hpf (Figures 2D; S3C–E, Supporting Information). A marked reduction in *drl* ^+^ cells (erythrocytes and their progenitors) was detected in *Tg* (*nap1l4a^−/−^*; *drl*:GFP^[^ ^25^ ^]^) embryos (Figures 2E; S3F,L, Supporting Information), and reduced numbers of mature erythrocytes in *Tg* (*LCR*: GFP) (Figure S3I, Supporting Information), and of erythrocytes and their progenitors in *Tg* (*gata1a*: DsRed) (Figure S3G,H,M,N, Supporting Information) embryos, were detected with *nap1l4a* deficiency, respectively. *Nap1l4a* ^−/−^ embryos showed a significant decrease in the expression of erythroid lineage specific TFs Tal1/Scl, Lmo2, Gata1a, and Klf1 at 24 hpf (Figure 2F,G), and *gata1a* expression was significantly down‐regulated at 14 hpf (Figure S3J, Supporting Information). Consistently, flow cytometry‐sorted (FASC) *drl* ^+^ cells from embryos injected with *nap1l4a* MOs showed significant reduction of *gata1a*, *scl*, *lmo2*, *klf1*, *hbbe1*, *hbbe2*, and *hbbe3* (Figure S3K, Supporting Information). Together, these findings demonstrate that *nap1l4a* is required for primitive erythropoiesis.

A significant decrease was observed in Annexin V‐PE‐labeled *drl* ^+^ cells in *nap1l4a* knockdown embryos at 24 hpf, as well as in TUNEL‐labeled *gata1a* ^+^ cells in *nap1l4a^−/^* ^−^ embryos. However, although the overall percentages of both *drl* ^+^ and *gata1a* ^+^ cells was markedly reduced in embryos with *nap1l4a* deficiency (Figure S3L,M, Supporting Information). These results suggest that apoptosis is unlikely to account for the primitive erythropoiesis defects. In contrast, *nap1l4a* ^−/−^ embryos exhibited a significant reduction in EdU*^+^gata1a^+^* cells at 24 hpf (Figure S3N, Supporting Information). These findings indicate that impaired cell proliferation is a likely contributor to the defective primitive erythropoiesis in *nap1l4a* deficient embryos.

We next investigated whether the primitive erythropoiesis defects were specific to *nap1l4a* ^−/−^ mutants. The mesodermal markers *sall4* and *pax2a*, the mesoderm progenitor *myod*, and ectodermal marker *six3b*, exhibited unchanged expression in *nap1l4a* ^−/−^ at 10 hpf (Figure S4A, Supporting Information). Expression of the vascular marker *flk1* was even expanded at 24 hpf (Figure S4B, Supporting Information). In contrast, the HSPC markers (*runx1* and *c‐myb*) (Figure S4C,D, Supporting Information) and the lymphoid marker *rag1* (Figure S4E, Supporting Information), showed markedly down‐regulated expression in *nap1l4a* ^−/−^ embryos. The myogenic regulatory factor *myod1* showed no significant change, whereas the fast muscle‐specific marker *myhz2* was significantly downregulated at 24 hpf (Figure S4F, Supporting Information), consistent with the GO terms enrichment of muscle related down‐regulated DEGs in the mutants (Figure S3A, Supporting Information). The expression patterns of the tested genes in *nap1l4a* ^−/−^ embryos at different developmental stages are summarized in Figure S4G (Supporting Information). *Nap1l4a* full‐length mRNA could effectively recover the transcriptional expression of *gata1a* and *hbbe3* in *nap1l4a* ^−/−^ at 24 hpf (Figure 2H), further demonstrating that *nap1l4a* is required to regulate primitive erythropoiesis specifically.

We further investigate the specificity of *nap1l4a* MOs by co‐injecting *p53*‐MO in this study. The expression levels of erythroid genes in embryos co‐injected with *nap1l4a* MO and *p53* MO were not significantly different from those injected with *nap1l4a* MO alone (Figure S5A,B, Supporting Information). These findings further demonstrate the specificity of *nap1l4a* MOs, indicating that disruption of *nap1l4a* does not affect overall embryogenesis, and that the mutants exhibit defects in primitive erythrogenesis and definitive hematopoiesis similar to those observed in *nap1l4a* knockdown embryos.^[^ ^6^ ^]^

### 2.2 Loss of Nap1l4a does not Broadly Perturb Transcription but Markedly Reduces Expression of Erythroid‐Specific Genes

**Figure 3:**
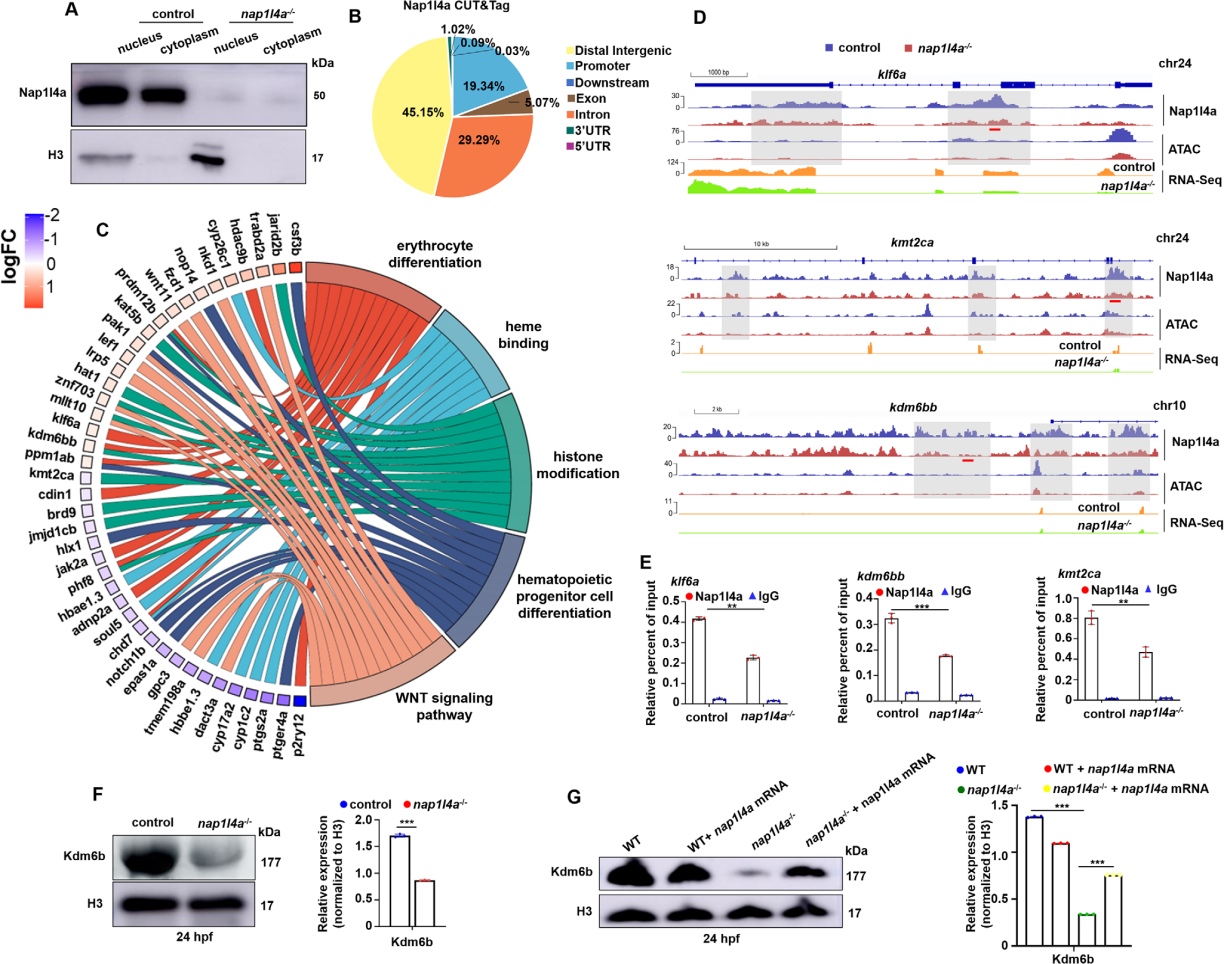
Nap1l4a functions as a transcriptional regulator in primitive erythropoiesis. A) Nap1l4a protein is prominently observed in both the cytoplasm and nucleus. B) Genome‐wide distribution of Nap1l4a CUT&Tag peaks, obtained after filtering non‐specific signals from *nap1l4a* ^−/−^ cells, with 19.34% of peaks located at promoter regions. C) Circular plot of 42 representative genes directly targeted by Nap1l4a, illustrating relationships between gene expression changes (left semicircle perimeter) and associated biological processes (right semicircle perimeter). Color indicates the log_2_ (fold change) value. D) IGV browser tracks showing Nap1l4a (1of n=2) binding profiles and corresponding targeted transcript levels for *klf6a*, *kmt2ca*, and *kdm6bb*, with ATAC (1of n=2) tracks indicating the chromatin accessibility. Gray‐shaded regions highlight differential binding enrichment of Nap1l4a and chromatin accessibility between WT and *nap1l4a* ^−/−^ embryos. E) ChIP‐qPCR analysis revealed reduced Nap1l4a binding on the promoters of genes *klf6a*, *kmt2ca*, and *kdm6bb*, respectively. Tested loci are underlined in red. F) Protein levels of Kdm6b in *nap1l4a* ^−/−^ and WT embryos. The tested loci are red underlined. G) The down‐regulated protein level of Kdm6b could be effectively rescued via injecting with full‐length *nap1l4a* mRNA in *nap1l4a* ^−/−^ at 24 hpf. Each experiment was repeated at least three times, and a representative result is shown. ImageJ was used for quantifying the WB signals in different groups. Data were analyzed by *t* test using GraphPad Prism 8.0. Data are presented as mean ± SD.**P < 0.01. ***P < 0.001.

NAP1L4 belongs to the histone chaperone family that mediates nucleosome assembly and disassembly.^[^ ^9^ ^]^ Most NAP1L proteins translocate into the nucleus at specific phage of the cell cycle,^[^ ^26^ ^]^ suggesting potential roles in nuclear transcriptional regulation. Here, we found that Nap1l4a was distributed in both the nucleus and cytoplasm, and its protein levels were exhibited markedly reduced in *nap1l4a* ^−/−^ embryos at 24 hpf (**Figure** **3**A). The above results indicate that impaired proliferation and erythrocyte differentiation occur in embryos with *nap1l4a* deficiency. To characterize the regulatory network of Nap1l4a in erythropoiesis, we performed CUT&Tag (Cleavage Under Targets and Tagmentation) and ATAC‐Seq (Assay for Transposase‐Accessible Chromatin with high‐throughput sequencing) on 10^5^ cells each from WT control and *nap1l4a* ^−/−^ embryos in 24 hpf. CUT&Tag profiling identified 3017 high‐confidence Nap1l4a binding sites (Table S3, Supporting Information), after filtering out non‐specific signals detected in *nap1l4a* ^−/−^ cells. Approximately 19.34% of Nap1l4a binding sites were located at promoters (within ±3 kb of transcription start site (TSS)), whereas 45.15% were distributed in distal intergenic regions (Figure 3B). This genome‐wide distribution suggests that Nap1l4a regulates transcription not only at promoters, but also through modulating chromatin architecture at distal regulatory elements.

To identify the targets and regulatory mechanisms of Nap1l4a in erythropoiesis, we integrated RNA‐Seq and CUT&Tag datasets, and identified 1510 genes (Table S4, Supporting Information) regulated by Nap1l4a. These genes were enriched in biological processes including erythrocyte differentiation, heme binding, histone modification, hematopoietic progenitor cell differentiation, and the WNT signaling pathway (Figures 3C; S6A, Supporting Information). By further integrating RNA‐Seq and CUT&Tag results with ATAC‐Seq, we found that Nap1l4a localized to transcriptional regulatory regions of primitive erythropoiesis genes (*adnp2a* and *klf6a* ^[^ ^27^, ^28^ ^]^), heme binding genes (*hbae1.3* and *hbbe1.3*), HSPC and myeloid regulators (*runx1* ^[^ ^29^ ^]^ and *chd7*), and epigenetic modifiers including histone demethylase and methyltransferases (*kdm6bb*, *kmt2ca*, and *jarid2b* ^[^ ^30^, ^31^, ^32^ ^]^) (Figures 3D,E; S6B,C, Supporting Information). IGV browser analysis revealed decreased chromatin accessibility at loci such as *kdm6bb*, *kmt2ca*, *klf6a* (Figure 3D) at the absence of Nap1l4a, suggesting that Nap1l4a is required to maintain chromatin accessibility at these sites. Consistent with the chromatin changes, these genes were significantly down‐regulated at the transcriptional or translational level in *nap1l4a* ^−/−^ mutants (Figures 3D,F,G; S6B,D, Supporting Information). Notably, ectopic expression of full‐length *nap1l4a* mRNA effectively restored the reduced Kdm6b protein levels in the mutants (Figure 3G). Together, these findings demonstrate that Nap1l4a is necessary but not solely sufficient for primitive erythropoiesis. Rather than broadly deregulating gene expression, Nap1l4a specifically modulates chromatin organization and transcription of key hematopoiesis and epigenetic regulators, such as *klf6a*, *kdm6bb*, and *kmt2ca*.

### 2.3 Nap1l4a Cooperates with Erythroid TFs Scl and Klf1 in Sequential Primitive Erythropoiesis

**Figure 4:**
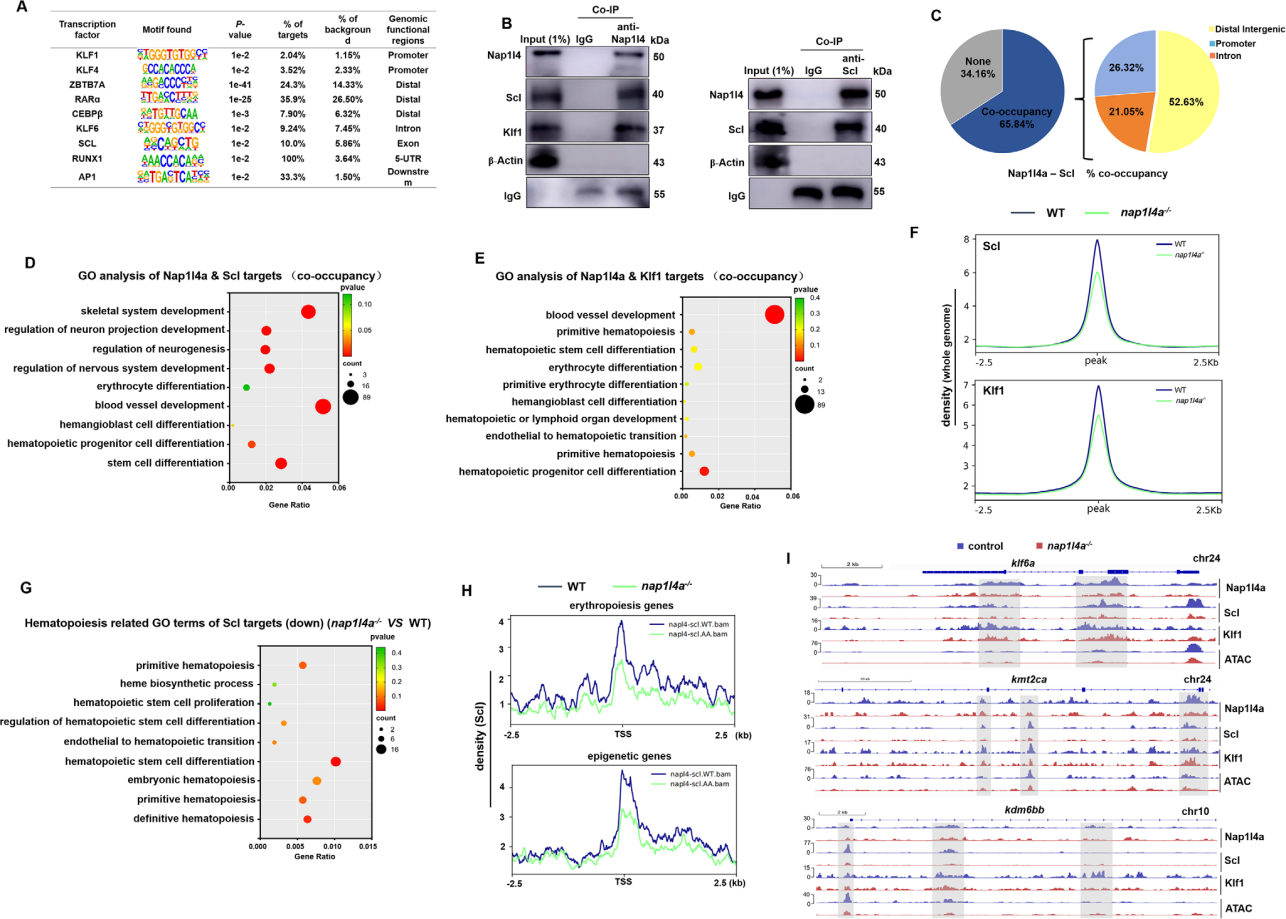
Nap1l4a cooperates with Scl and Klf1 to regulate erythropoiesis. A) Nap1l4a shows significantly enrichments on DNA‐binding motifs of hematopoietic and erythroid TFs, including CEBP, KLF factors, SCL, ZBTB7A, and others, as identified by HOMER known motif analysis. B) Co‐IP analysis showing that Nap1l4 physically interacts with Scl and Klf1. Each experiment was repeated at least three times, and a representative result is shown. C) Co‐occupancy of Nap1l4a and Scl (1of n=2) during zebrafish embryogenesis at 24 hpf, and the two proteins sharing 65.84% co‐occupancy. 52.63% co‐occupancy loci distribute in distal intergenic. D) Bubble plot showing GO enrichment of Nap1l4a and Scl co‐occupancy loci associated genes. E) Bubble plot showing GO enrichment of Nap1l4a and Klf1 (1of n=2) co‐occupancy loci associated genes. F) Scl and klf1 density on whole genome, centered in ±2.5kb around the TSS, in *nap1l4a* ^−/−^ and WT embryos. G) Bubble plot showing hemopoiesis‐related GO terms of down‐regulated Scl targets in *nap1l4a* ^−/−^ relative to WT embryos at 24 hpf. H) Scl density on representative erythropoiesis and epigenetic genes, respectively, centered ±2.5kb around the TSS, in *nap1l4a* ^−/−^ and WT embryos. I) IGV browser tracks showing binding profiles of Nap1l4a (1of n=2), Scl (1of n=2), and Klf1 (1of n=2) on genes *klf6a*, *kmt2ca*, and *kdm6bb*, respectively, with ATAC tracks (1of n=2). Gray‐shaded regions highlight differential binding enrichment of Nap1l4a and proteins Klf1 and Scl between WT and *nap1l4a* ^−/−^ embryos. GO and KEGG enrichment analyses using ClusterProfiler (v 4.8.1) R package. ImageJ was used for quantifying the WB signals in different groups. Data were analyzed by *t* test using GraphPad Prism 8.0. Each experiment was repeated at least three times, and a representative result is shown.

Previous studies reported that NAP1L4 is predicted to interact with P300^[^ ^33^ ^]^ which mediates H3K27ac acetylation,^[^ ^15^ ^]^ suggesting that NAP1L4 may participate in shaping the enhancer landscape and promoting transcriptional activation during hematopoiesis. However, the TFs responsible for recruiting Nap1l4a and facilitate subsequent P300 deposition remain unknown. Using HOMER known motif analysis of Nap1l4a occupancy sites, we identified significant enrichment of sequence motifs corresponding to hematopoietic and erythroid TFs, including KLF1, KLF4, KLF6, CEBPβ, RARα, SCL, RUNX1, and AP1 (**Figure** **4**A). We also identified enrichment for BCL11A and TCF3. Notably, CEBPβ cooperates with P300, RUNX1 and RARα, to regulate HSPC development,^[^ ^34^ ^]^ whereas KLF1, KLF4, KLF6, and SCL are important TFs for erythropoiesis.^[^ ^12^, ^13^, ^35^ ^]^ Here, endogenous Co‐immunoprecipitation (Co‐IP) assays unveiled physical interactions between Nap1l4a and Scl, as well as between Nap1l4a and Klf1 (Figure 4B). These findings suggest that Nap1l4a acts in concert with Klf1 and Scl within the same regulatory complex to promote erythropoiesis, and further support the notion that Nap1l4a may associate with the P300/CEBP complex as recently reported.^[^ ^33^ ^]^

Nap1l4a shared 65.84% co‐occupancy with Scl during zebrafish embryogenesis at 24 hpf (Figure 4C). Among these co‐binding sites, 52.62% were located in distal intergenic regions, and 26.32% in promoter regions (Figure 4C; Table S5, Supporting Information). Genes associated with these loci were enriched for biological processes such as hemangioblast cell differentiation, erythrocyte differentiation, blood vessel development, nervous system development, skeletal system development (Figure 4D; Table S6, Supporting Information). Similarly, Nap1l4a shared 66.83% co‐occupancy with Klf1, with 52.59% of the shared sites located in distal intergenic regions and 22.96% in promoters (Figure S7A and Table S7, Supporting Information). Genes associated with these Nap1l4a‐Klf1 co‐binding regions were enriched for pathways involved in blood vessel development, primitive hematopoiesis, hematopoietic stem cell differentiation (Figure 4E; Table S6, Supporting Information).

Scl and Klf1 displayed an overall reduction in genome‐wide binding density in *nap1l4a^−/−^* mutants (Figure 4F). Notably, 86.63% of Scl‐binding sites, and 92.78% of Klf‐binding sites, showed largely unchanged chromatin occupancy in *nap1l4a^−/−^* cells (Figure S7B, Supporting Information). Only 11.13% Scl‐binding sites exhibited increased binding and 0.74% showed decreased binding, whereas for Klf1, 6.85% Klf1‐binding sites exhibited increased binding and 0.38 % showed decreased binding in the mutants (Figure S7B and Table S8, Supporting Information). Genes associated with the 0.74% of Nap1l4a‐Scl dependent loci and 0.38% of Nap1l4a‐Klf1 dependent loci (Figure S7B, Supporting Information) were enriched for GO terms related to primitive hematopoiesis, heme biosynthetic process, hematopoietic stem cell proliferation, mesenchyme development (Figures 4G; S7C,E, Supporting Information). These loci associated genes were also enriched for GO terms associated with chromatin organization and iron ion homeostasis (Figure S7F and Table S9, Supporting Information).By contrast, loci with increased Scl (11.13%) or Klf1 (6.85%) occupancy were enriched for GO terms related to vascular process, ventricular system development, neuroblast division, B cell mediated immunity (Figure S7D and Table S10, Supporting Information). Collectively, these findings demonstrate that Nap1l4a is largely dispensable for Scl and Klf1 binding at the majority of their genomic targets. However, the less than 1% of loci whose occupancy depends on Nap1l4a primarily regulate genes involved in erythropoiesis and epigenetic processes.

Overall, Scl exhibited reduced densities at erythropoiesis‐related and epigenetic genes loci in the mutants (Figure 4H). At co‐occupied regulatory regions of the erythroid transcription factor *klf6a*, the chromatin binding of Nap1l4a, Scl, and Klf1 was jointly diminished in *nap1l4a* ^−/−^ embryos (Figure 4I). A similar reduction in occupancy was also observed at the regulatory regions of the epigenetic modifiers *kmt2ca* and *kdm6bb* (Figure 4I). The decreased chromatin accessibility at the aforementioned occupancy loci were observed at the absence of Nap1l4a, suggesting that Nap1l4a is also required to maintain chromatin accessibility at these sites. These findings demonstrate a previously unrecognized role of Nap1l4a in erythropoiesis and epigenetic regulation through co‐occupancy with the erythrocyte TFs Scl and Klf1, demonstrating that Nap1l4a is required for Scl and Klf1 binding at key erythropoiesis and epigenetic genes and the consequent chromatin accessibility.

### 2.4 Nap1l4a Orchestrates Chromatin Accessibility Dynamics Governing Primitive Erythropoiesis

**Figure 5:**
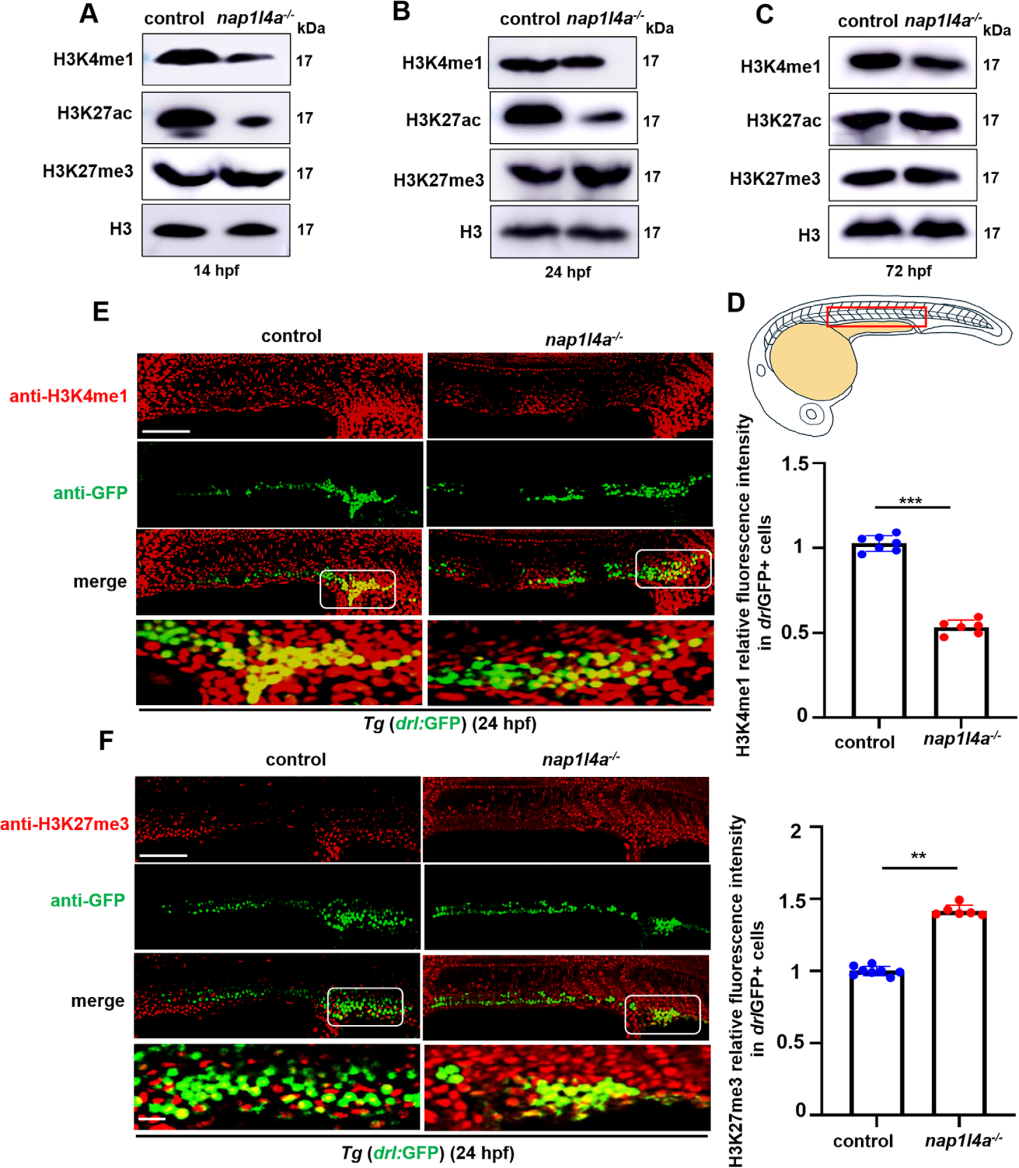
Effects of *nap1l4a* deficiency on proteins H3K27ac, H3K4me1 and H3K27me3 in embryos and erythrocytes. A−C) Protein levels of H3K27ac, H3K4me1, and H3K27me3 in *nap1l4a* ^−/−^ and WT embryos at A) 14 hpf, B) 24 hpf, and C) 72 hpf. D) Representative immunofluorescence domain in embryos. E,F) Double staining of *drl*GFP^+^ (green) and H3K4me1 (red) (E), and *drl*GFP^+^ (green) and H3K27me3 (red) (F), in *nap1l4a^−/−^* and WT embryos at 24 hpf, respectively, and graphs show the quantitative analysis of H3K4me1 and H3K27me3 fluorescence intensity in *drl*GFP^+^. Regions within white boxes in (E,F) are at higher magnification. Each experiment was repeated at least three times, and a representative result is shown. ImageJ was used for quantifying the WB and fluorescence signals in different groups. Data were analyzed by *t* test using GraphPad Prism 8.0. Data are presented as mean ± SD. ***P < 0.001, **P < 0.01, Scale bars, 100 µm, and 40 µm in higher magnifications (E,F).

Nucleosome assembly proteins (NAPs) have been implicated in the regulation of covalent histone modifications and epigenetic transcription.^[^ ^10^ ^]^ Our data indicate that *nap1l4a* deficiency induces pronounced transcriptional and translational changes in histone demethylase and methyltransferases (Figures 3F,G; S6E, Supporting Information). Consistently, *nap1l4a* ^−/−^ embryos exhibited a marked reduction in H3K4me1 and H3K27ac levels, accompanied by an increase in H3K27me3 at both 14 hpf and 24 hpf (**Figures** **5**A,B; S8A, Supporting Information). Notably, these histone modification levels were restored at 72 hpf in the mutants (Figures 5C; S8A, Supporting Information).

A significant reduction in the number of *drl* ^+^ cells was observed in *nap1l4a* deletion embryos, accompanied by decreased intracellular H3K4me1 levels and increased H3K27me3 levels in these erythrocytes (Figure 5E,F). Representative immunofluorescence images of the embryos are shown in Figure 5D. These findings indicate that *nap1l4a* deficiency alters histone modification patterns in both *nap1l4a* ^−/−^ embryos and their erythrocytes.

**Figure 6:**
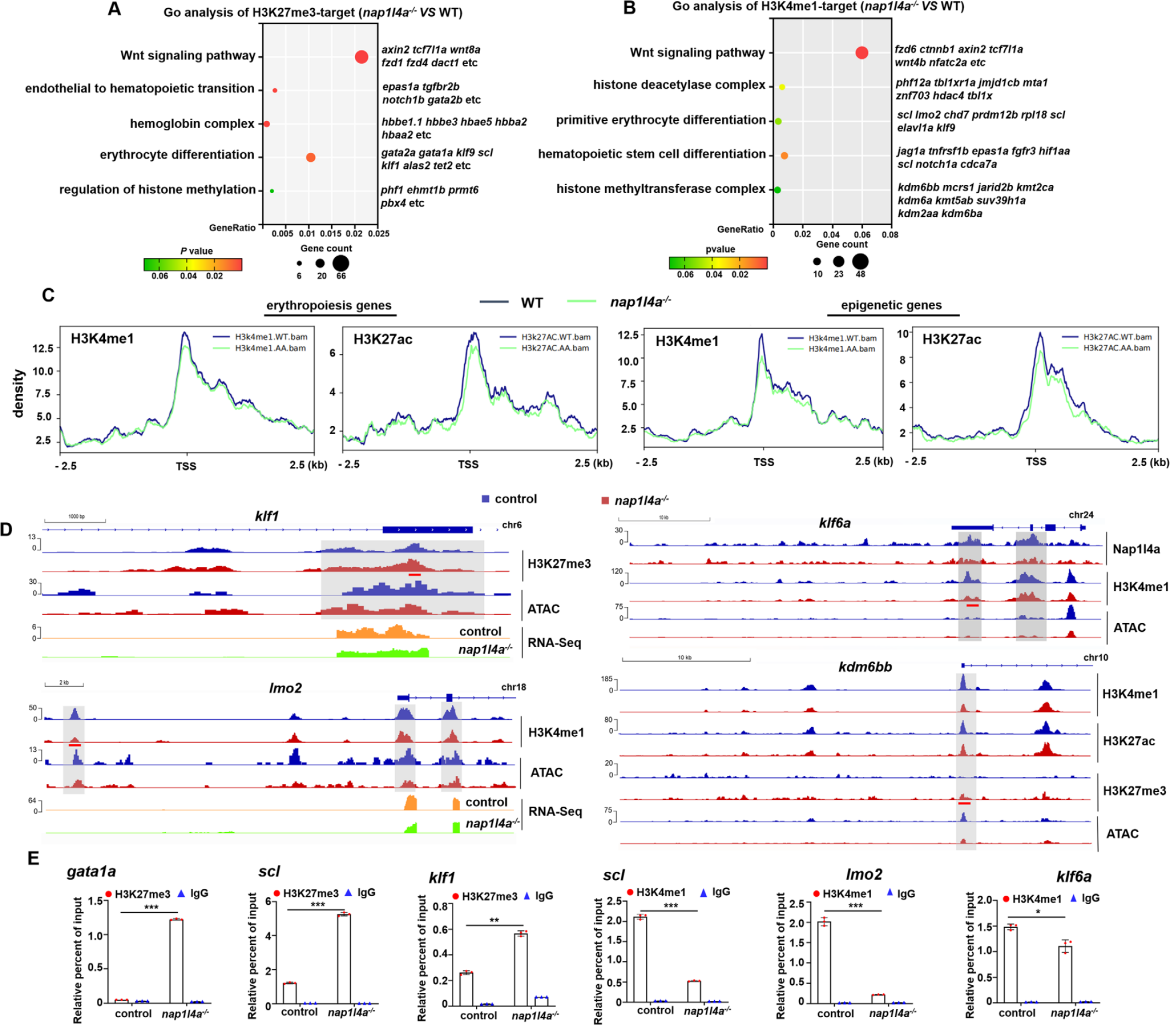
Nap1l4a regulates erythropoiesis by modifying the epigenetic histone landscapes. A,B) Bubble plot showing GO enrichment of transcriptional DEGs with altered binding enrichment of H3K27me3 (A) and H3K4me1 (B). C) H3K4me1 and H3K27ac density on representative erythropoiesis and epigenetic genes, respectively, centered ±2.5kb around the TSS, in *nap1l4a* ^−/−^ and WT embryos. D) IGV browser tracks showing binding profiles of H3K27me3 (1of n=2), H3K4me1 (1of n=2), and H3K27ac (1of n=2) on genes *klf1*, *klf6a*, *lmo2*, and *kdm6bb*, respectively, with ATAC tracks (1of n=2). Gray‐shaded regions highlight differential binding enrichment loci. E) Enrichment of H3K27me3 or H3K4me1 binding (by ChIP‐qPCR) on promoter or enhancer regions of targeted genes in WT and *nap1l4a* ^−/−^. Tested loci are underlined in red. Each experiment was repeated at least three times, and a representative result is shown. GO and KEGG enrichment analyses using ClusterProfiler (v 4.8.1) R package. Data were analyzed by *t* test using GraphPad Prism 8.0. Data are presented as mean ± SD. ***P < 0.001, **P < 0.01, *P < 0.05.

Numerous studies have demonstrated that histone modifications play critical roles in erythrocyte development.^[^ ^17^, ^36^ ^]^ Therefore, we further examined histone epigenomic profiles in *nap1l4a* ^−/−^ mutants. Integration of CUT&Tag and transcriptomic data revealed that genes with altered H3K4me1, H3K27ac, or H3K27me3 binding in the *nap1l4a* ^−/−^ mutants were enriched in biological processes including erythrocyte differentiation, heme binding, histone modification, hematopoietic progenitor cell differentiation, and the WNT signaling pathway (**Figures** **6**A,B; S9 and Tables S11–S13, Supporting Information). Both H3K4me1 and H3K27ac exhibited a little general reduction in chromatin density at erythropoiesis and epigenetic genes (Figure 6C), suggesting that integral Nap1l4a is required to propagate the regulatory landscape of these genes.

In *nap1l4a* ^−/−^ mutants, the enrichment of histone H3K27me3 was increased at the suppressor regions of erythroid TFs and heme binding genes, including *klf1*, *scl*, *gata1a*, *hbbe2*, *hbbe1.3*, and *kdm6bb* (Figures 6D,E; S10A,B, Supporting Information). A reduction in chromatin accessibility was observed at the loci of these genes (Figures 3D and 6D). Furthermore, the binding of H3K4me1 or H3K27ac at enhancer regions (Figures 6D,E; S10A,B, Supporting Information) were significantly decreased in *klf6a*,^[^ ^28^ ^]^ *lmo2*, *kdm6ba*,^[^ ^37^ ^]^ *kdm6al*,^[^ ^38^ ^]^ and *kmt2bb*,^[^ ^39^ ^]^ in addition to the genes mentioned above. All these genes exhibited significantly down‐regulated expressions in *nap1l4a* ^−/−^ mutants (Figures 2; S6D, Supporting Information). These findings support the mutually exclusive nature of H3K27ac and H3K27me3 binding,^[^ ^40^ ^]^ further suggesting that Nap1l4a regulates erythropoiesis by remodeling histone epigenomes and the associated regulatory landscapes.

Moreover, the enrichment of histone H3K27me3 was significantly reduced at adult hemoglobin genes, including *hbaa2*, *hbaa1*, *hbba2*, and *hbba1* (Figure S10A,B, Supporting Information).^[^ ^41^ ^]^ Using HOMER tool, we found that Nap1l4a may interact with Bcl11a, which is reported to repress γ‐globin gene expression to regulate the fetal‐to‐adult hemoglobin switch.^[^ ^42^ ^]^ These results suggest that Nap1l4a may be required to suppress the fetal‐to‐adult hemoglobin switch through cooperating with Bcl11a.

### 2.5 Nap1l4a Modulates Chromatin Accessibility via Recruiting H2A.Z

**Figure 7:**
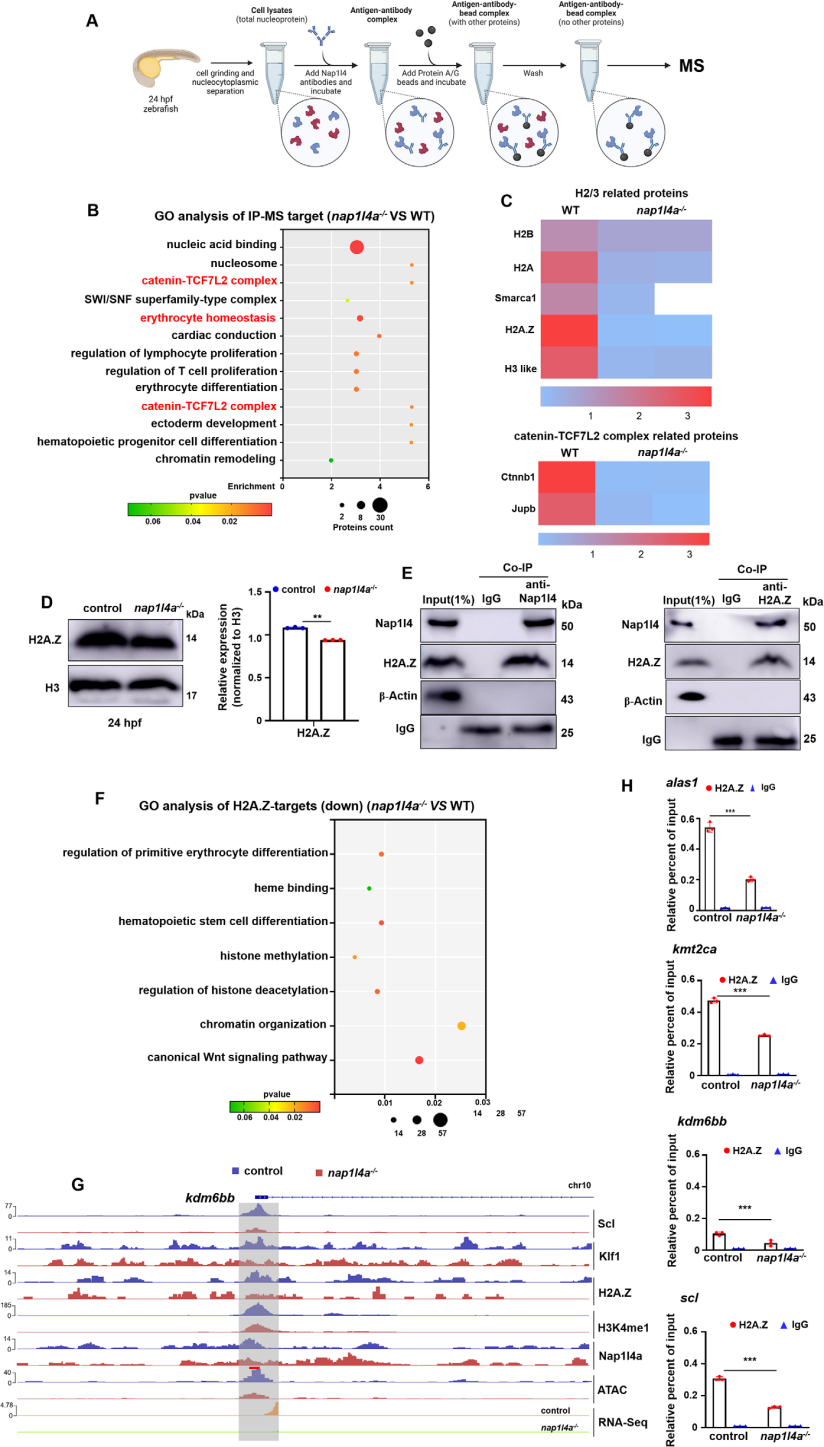
Nap1l4a recruits H2A.Z to modulate chromatin organization and histone modification during erythropoiesis. A) Schematic overview of Nap1l4 immunoprecipitation‐mass spectrometry (IP‐MS). B) GO functional annotation of differentially enriched proteins (WT vs *nap1l4a* ^−/−^) identified by IP‐MS using Nap1l4 antibody. 2 biological repeats. C) Heatmaps of H2/3 and Catenin‐TCF7L2 complex related proteins (WT vs *nap1l4a* ^−/−^) identified by IP‐MS using Nap1l4 antibody. D) Protein levels of H2A.Z in *nap1l4a* ^−/−^ and WT embryos at 24 hpf. E) Co‐IP showing that Nap1l4 and H2A.Z were parts in the same complex. F) Bubble plot showing GO enrichment of transcriptional DEGs with altered H2A.Z binding (1of n=2). G) Binding profiles of H2A.Z (1of n=2) at representative target genes *kdm6bb* and *klf6a*, visualized in the IGV browser, with tracks of different epigenetic proteins and ATAC (1of n=2, each). H) Enrichment of H2A.Z binding (by ChIP ‐ qPCR) on promoter or enhancer regions of targeted genes in WT and *nap1l4a* ^−/−^. Tested loci are underlined in red. Each experiment was repeated at least three times, and a representative result is shown. Signals from WB images were quantified with ImageJ. Data were analyzed by *t* test using GraphPad Prism 8.0. Data are presented as mean ± SD. ***P < 0.001.

In this study, we observed that Nap1l4a deficiency induced chromatin compaction at key target genes. To further investigate proteins that interact with Nap1l4a and contribute to its role in chromatin accessibility and subsequent activation of erythropoiesis and epigenetic genes, we performed immunoprecipitation‐mass spectrometry (IP‐MS) using a Nap1l4 antibody at 24 hpf, along with *nap1l4a* ^−/−^ embryos, to identify differentially bound proteins (**Figure** **7**A; Table S14, Supporting Information). The differential bound proteins identified by IP‐MS were enriched in terms of Catenin‐TCF7L2 complex, H2/3 related proteins, erythrocyte homeostasis complex (Figures 7B,C; S11A, Supporting Information). Among these, H2A.Z and the WNT signaling transcriptional factor β‐Catenin (Ctnnb1) were of particular interest (Figure 7C).

We found that H2A.Z levels was reduced in *nap1l4a* ^−/−^ mutants (Figure 7D), and demonstrated that Nap1l4a and histone H2A.Z were components of the same complex (Figure 7E). The H2A.Z variant facilitates the recruitment and chromatin binding of CBP and P300, thereby promoting chromatin opening and enhancing HSPC gene expression.^[^ ^33^ ^]^ We next asked whether Nap1l4a cooperates with H2A.Z to facilitate its deposition and subsequent chromatin opening at erythropoiesis genes. H2A.Z shared 41.09% co‐occupancy with Nap1l4a (Figure S11C, Supporting Information), and genes associated with these co‐occupancy loci were enriched in GO terms elated to regulation of neurogenesis, canonical WNT signaling, primitive erythrocyte differentiation (Figure S11B,E, Supporting Information).

Only 0.50% of H2A.Z binding loci exhibited reduced enrichment in *nap1l4a^−/^* ^−^ embryos at 24 hpf, and genes associated with these loci were enriched in GO terms, such as primitive erythrocyte differentiation, heme binding, epigenetic modulation, iron ion homeostasis, the WNT signaling pathway, based on integrative analysis of CUT&Tag and transcriptome data (Figures 7F; S11B–G and Table S15, Supporting Information). In *nap1l4a* ^−/−^ mutants, both H2A.Z enrichment and chromatin accessibility were reduced at enhancer regions of key epigenetic and hematopoiesis genes, including *kmt2ca*, *kdm6bb*, *klf6a*, *scl*, *alas1* (the key enzyme in heme biosynthesis),^[^ ^43^ ^]^ *nfe2*,^[^ ^44^ ^]^ and *runx1* in the absence of Nap1l4a at these same binding sites, accompanied by reduced H3K27ac enrichment (Figures 7G,H; S11H, Supporting Information). These findings further demonstrate that Nap1l4a recruits H2A.Z to loci of erythrogenesis and epigenetic genes to promote chromatin opening and transcriptional activation.

### 2.6 Epigenetic Histone Modifier Treatment Mimics the Primitive Erythropoiesis Defects

**Figure 8:**
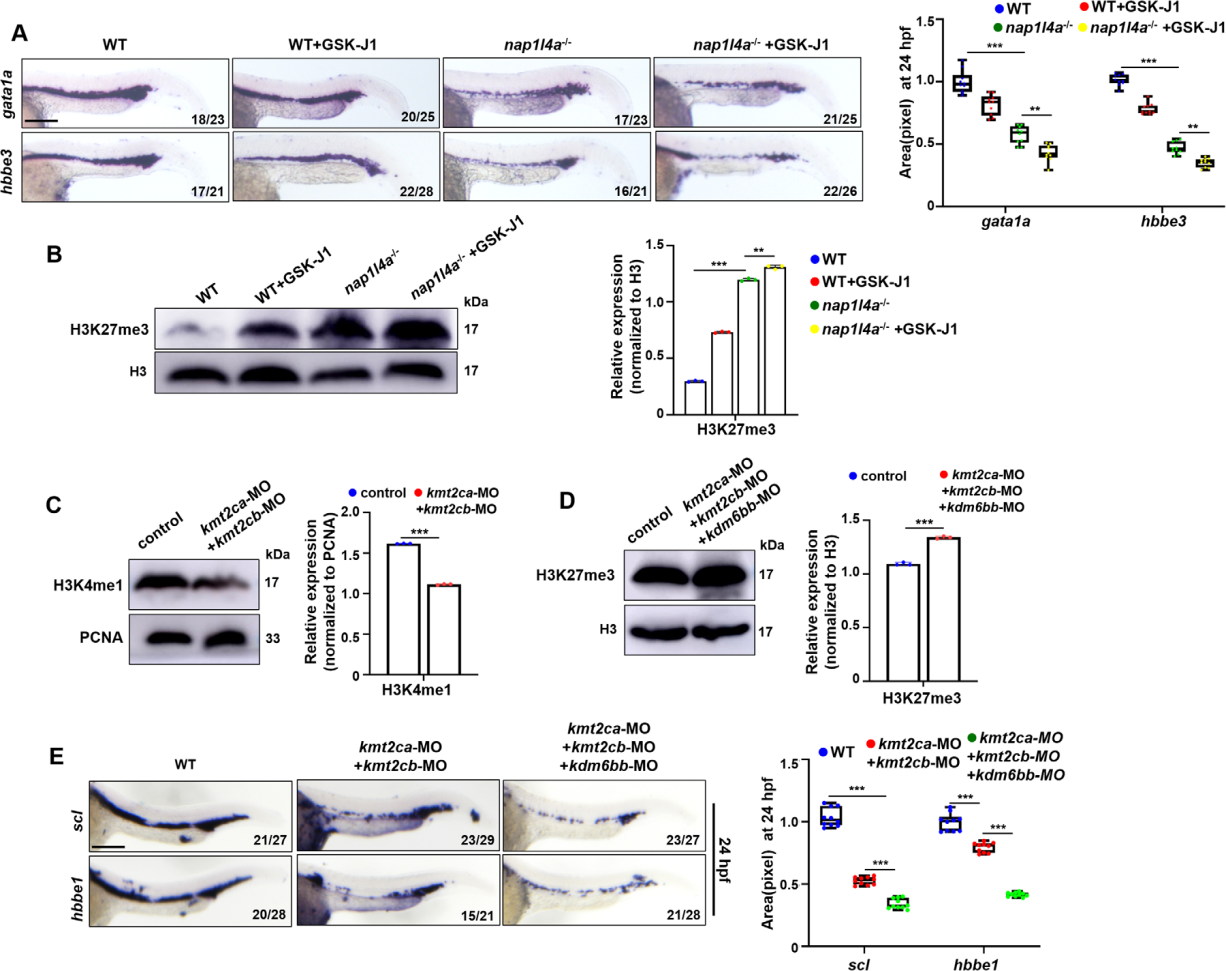
Epigenetic histone modifiers Kmt2ca, Kmt2cb, and Kdm6bb mediate Nap1l4a function in regulating primitive erythropoiesis. A) WISH analysis of *gata1a* and *hbbe3* expression in *nap1l4a* ^−/−^ and WT embryos and in embryos treated with GSK‐J1 at 24 hpf, and box and whisker plots show relative transcriptional levels across groups. B) Protein levels of H3K27me3 in *nap1l4a* ^−/−^ and WT embryos and in embryos treated with GSK‐J1 at 24 hpf, and graphs show the relative protein levels. C) Protein levels of H3K4me1 in WT embryos and embryos injected with both *kmt2ca* and *kmt2cb* morpholinos (MO) at 24 hpf, and graphs show relative protein levels. D) Protein levels of H3K27me3 in WT embryos and embryos injected with *kmt2ca*, *kmt2cb*, and *kdm6bb* MO at 24 hpf, and graphs show relative protein levels. E) WISH analysis of *scl* and *hbbe1* expression in WT embryos, embryos injected with both *kmt2ca* and *kmt2cb* MOs, and embryos co‐injected with *kmt2ca*, *kmt2cb*, and *kdm6bb* MOs at 24 hpf, and box and whisker plots show relative transcriptional levels. Each experiment was repeated at least three times, and a representative result is shown. Signals from WB and WISH images were quantified with ImageJ. Data were analyzed by *t* test using GraphPad Prism 8.0. Data are presented as mean ± SD. A,E) lateral view, anterior to the left. ***P < 0.001, **P < 0.01, Scale bars, 75 µm (A,E).

We next investigated whether the down‐regulation of *kmt2cb*, *kmt2ca*, *kdm6bb* and histone demethylases and methyltransferases, leads to altered histone modification levels, thereby contributing to primitive erythropoiesis defects in the mutants. GSK‐J1, an inhibitor of the histone H3K27me3 demethylase activity of KDM6B both in vitro and in vivo,^[^ ^45^ ^]^ was applied from 11 hpf to both WT control and *nap1l4a* ^−/−^ embryos. GSK‐J1 treatment induced a significant reduction in *gata1a* and *hbbe3* expression in WT embryos, with an even greater reduction observed in *nap1l4a* ^−/−^ embryos at 24 hpf (**Figure** **8**A). Furthermore, GSK‐J1 treatment significantly increased H3K27me3 levels in WT embryos, with an even higher increase in *nap1l4a* ^−/−^ embryos at 24 hpf (Figure 8B). These findings demonstrate that Nap1l4a deficiency induced elevated H3K27me3 levels impair primitive erythropoiesis.

*Kmt2ca* and *kmt2cb* morphants, injected with both *kmt2ca* and *kmt2cb* morpholinos (MOs), exhibited a marked decrease in H3K4me1 levels throughout the embryos (Figure 8C) and a significant reduction in *scl* and *hbbe1* transcription (Figure 8E) at 24 hpf. Furthermore, morphants co‐injected with *kdm6bb*, *kmt2ca*, and *kmt2cb* morpholinos (MOs) exhibited a pronounced increase in H3K27me3 levels throughout the embryos (Figure 8D) accompanied by an even more severe reduction in *scl* and *hbbe1* transcription (Figure 8E). These observations suggest that either treatment with the histone modifier GSK‐J1 or knockdown of *kmt2ca*/*cb* and *kdm6bb*, leads to defects in primitive erythropoiesis that recapitulate those observed in *nap1l4a* ^−/−^ mutants. Collectively, these findings further demonstrate that *nap1l4a* maintains the epigenetic landscape required for normal erythroid gene expression, and directly targets *kdm6bb*, *kmt2ca, kmt2cb* to maintain normal epigenetic landscapes.

### 2.7 Nap1l4a is Required for WNT Pathway Activities in Primitive Erythrogenesis

**Figure 9:**
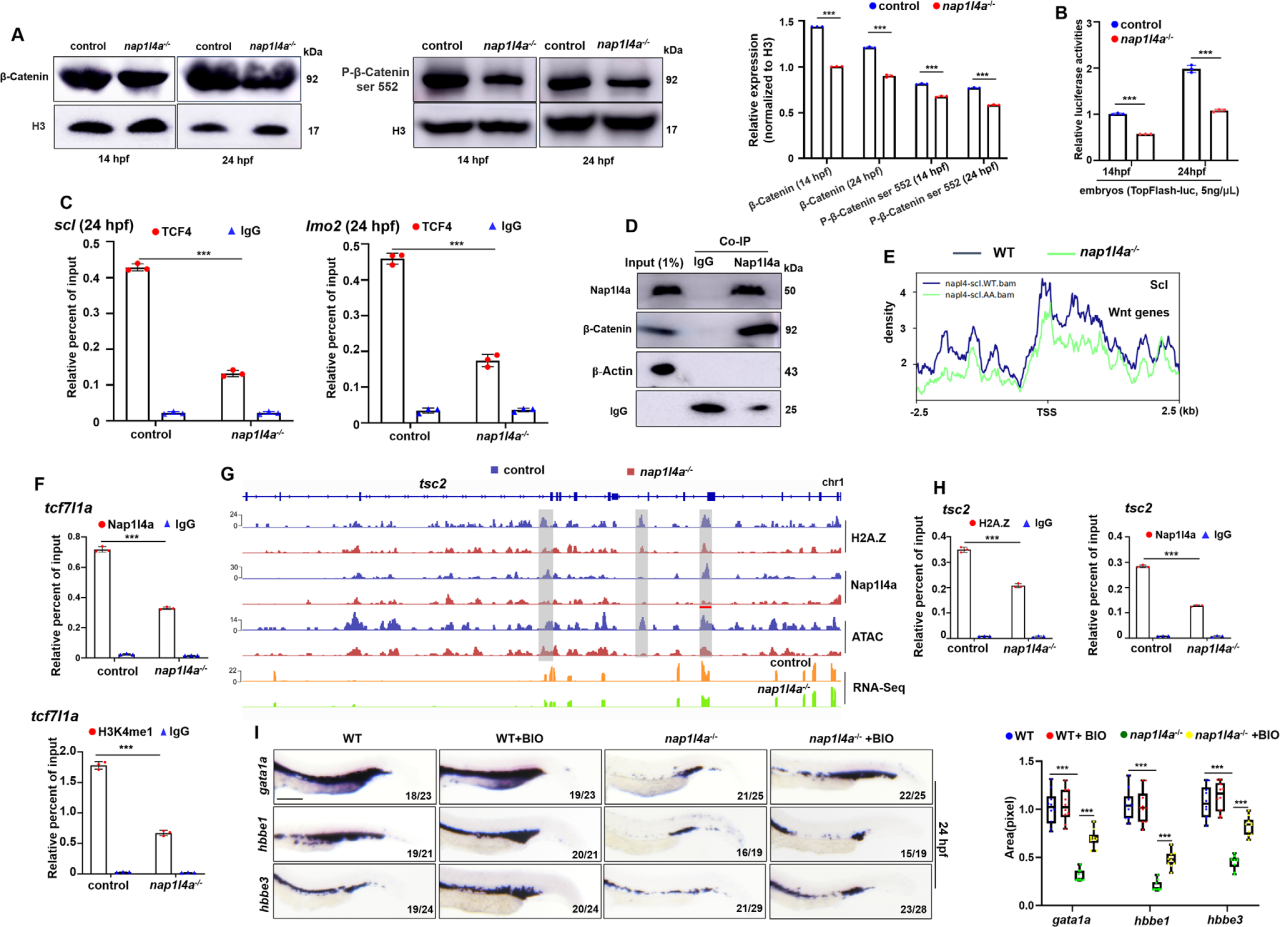
WNT/β‐Catenin signaling mediates *nap1l4a* function in primitive embryogenesis. A) Protein levels of β‐Catenin and P‐β‐Catenin ser 552 in *nap1l4a* ^−/−^ and WT embryos at 14 and 24 hpf, and graphs show relative protein levels of each sample. B) Luciferase activity assays of WNT/β‐Catenin reporter in zebrafish *nap1l4a* ^−/−^ and WT embryos at 14 and 24 hpf. C) Reduced binding enrichment of TCF4 on the promoters of *scl* and *lmo2*, as revealed by ChIP‐qPCR assays. Anti‐TCF4 was used for ChIP‐qPCR assays in *nap1l4a* ^−/−^ and WT embryonic cells, with anti‐IgG as the negative control. D) Co‐IP showing that Nap1l4 physically interacts with β‐Catenin. E) Scl (1of n = 2) density on representative WNT genes, centered ±2.5kb around the TSS, in *nap1l4a* ^−/−^ and WT embryos. F) Enrichment of Nap1l4a and H3K4me1 binding (by ChIP ‐ qPCR) on promoter or enhancer regions of targeted gene *tcf7l1a* in WT and *nap1l4a* ^−/−^ embryos. G) Binding profiles of representative H2A.Z and Nap1l4a target genes *tsc2*, visualized in the IGV browser, with ATAC tracks (1 of n = 2). H) Enrichment of Nap1l4 and H2A.Z (by ChIP‐qPCR) on promoter or enhancer regions of *tsc2*. Tested loci were underlined in red. I) WISH analysis of *gata1a*, *hbbe3* and *hbbe3* expression in *nap1l4a* ^−/−^ and WT embryos and in embryos treated with BIO at 24 hpf, and box and whisker plots show relative transcription levels across groups. Each experiment was repeated at least three times, and a representative result is shown. Signals from WB and WISH images were quantified with ImageJ. Data were analyzed by *t* test using GraphPad Prism 8.0. Data are presented as mean ± SD. I, lateral view, anterior to the left. ***P < 0.001. Scale bars, 75 µm (I).

The above data suggest that Nap1l4a is required for the regulation of WNT signaling (Figures 7F; S9, Supporting Information). The WNT/β‐Catenin pathway is well known to play crucial roles in hematopoietic development, including early hematopoiesis and primitive erythrogenesis.^[^ ^46^ ^]^ In this study, transcriptomic analysis revealed that the WNT/β‐Catenin signaling pathway was markedly disrupted in *nap1l4a* ^−/−^ mutants (Figure S12A,B, Supporting Information). The expression of the WNT/β‐Catenin signaling indicator gene *axin2* and the Wnt ligand *wnt3*, was significantly reduced in *nap1l4a* ^−/−^ embryos at both14 hpf and 24 hpf (Figure S12C–E, Supporting Information). Protein levels of β‐Catenin and Phospho‐β‐Catenin (Ser552) (P‐β‐Catenin‐Ser552), were also significantly decrease in *nap1l4a* ^−/−^ embryos (**Figure** **9**A). In vivo embryonic TopFlash luciferase reporter assays, monitoring WNT/β‐Catenin transcriptional activity, revealed substantially weakened reporter signals in *nap1l4a* ^−/−^ embryos at both 14 and 24 hpf (Figure 9B), indicating down‐regulation of WNT signaling in *nap1l4a* ^−/−^ mutant. Additionally, *nap1l4a* ^−/−^ embryonic cells exhibited a significant reduction in TCF4 binding at the promoters of *scl* and *lmo2* (Figure 9C), whereas no significant change was observed at the *gata1a* promoter (Figure S12F, Supporting Information). Furthermore, a physical interaction between Nap1l4a and β‐Catenin was identified (Figure 9D), suggesting that Nap1l4a interacts with β‐Catenin to regulate WNT signaling.

We observed an overall decrease in Scl binding density at genes involved in the WNT signaling pathway (Figure 9E). Along with Nap1l4a and H3K4me1, Scl exhibited reduced binding enrichment at key WNT signaling genes, including *tcf7l1a*,^[^ ^47^ ^]^ *dact3a*,^[^ ^48^ ^]^ *fzd1*,^[^ ^49^ ^]^ *lef1*,^[^ ^50^ ^]^ and *tsc2*,^[^ ^51^ ^]^ in *nap1l4a* ^−/−^ mutants, accompanied by a marked decrease in chromatin accessibility (Figures 9F–H; S12G, Supporting Information). These genes were all significantly down‐regulated in *nap1l4a* ^−/−^ mutants (Figure S12H, Supporting Information). Moreover, the decreased expression of *gata1a*, *hbbe1* and *hbbe3* in *nap1l4a* ^−/−^ mutants was effectively rescued by treatment with the WNT agonist BIO at 24 hpf (Figure 9I). Taken together, these results demonstrate that *nap1l4a* is critical for primitive erythrogenesis by modulating WNT/β‐Catenin signaling, through maintaining β‐Catenin stability and physically interacting with β‐Catenin to recruit H2A.Z, thereby promoting chromatin accessibility at WNT target genes and facilitating their transcription. The role of Nap1l4a in erythropoiesis is analogous to PGE2 role in promoting hematopoietic stem cell fate via regulation of the Wnt pathway,^[^ ^52^ ^]^ and in modulating chromatin through the H2A.Z‐variant.^[^ ^52^ ^]^

## 3 Discussion

Nap1l4a belongs to the NAP1L protein family and functions as a histone chaperone.^[^ ^8^ ^]^ NAP1L proteins have been reported to participate in tumor progression, histone transport, and cell cycle progression.^[^ ^53^, ^54^ ^]^ However, the roles of NAP1L4 in erythropoiesis, hypoxia tolerance and related diseases remain unclear. Here, we reveal that zebrafish *nap1l4a* ^−/−^ embryos and larvae are sensitive to hypoxic stress and exhibit defects in primitive erythropoiesis. Nap1L4a physically interacts with erythropoiesis TFs Scl and Klf1 to recruit the H2A.Z variant, thereby influencing nucleosome assembly and histone acetylation, ultimately promoting chromatin accessibility and transcriptional activation of erythropoiesis and epigenetic genes. Nap1L4a deficiency results in chromatin compaction at loci such as *kdm6bb* and *kmt2ca*, resulting in altered deposition of their histone targets H3K27me3 and H3K4me1 at erythropoiesis‐related genes in *nap1l4a* ^−/−^ mutants. These findings reveal the integral role of Nap1l4a in maintaining chromatin structure and gene transcription during primitive erythropoiesis, which in turns affects hypoxia tolerance in zebrafish. The novel transcriptional and epigenetic functions of Nap1l4a uncovered in this study provide valuable insights into the molecular mechanisms underlying erythropoiesis.

This study demonstrates that loss of *nap1l4a* disrupts erythrocyte differentiation and formation, as well as the maintenance of HSPCs, while leaving vascular cells and germ layers development largely unaffected. These findings explain the high expression of NAP1L4 in MEPs and erythroid cells, underscoring its critical role in normal erythroid differentiation. This role may also account for the strong association of NAP1L4 mutations or deletions with hematopoietic malignancies, such as acute myeloid leukemia. Erythrocytes are essential for oxygen transport, and defects in their developmental can lead to early‐onset fatal hypertrophic cardiomyopathy and other human diseases.^[^ ^55^ ^]^ For instance, high‐altitude populations exhibit increased erythrocyte production as an adaptive response to hypoxia.^[^ ^56^ ^]^ Together, these finding reveal that Nap1l4a is required for erythrocyte development, highlighting its fundamental role in maintaining hematopoietic homeostasis and promoting hypoxia tolerance.

We reveal that Nap1l4a functionally interacts with the erythropoiesis TFs Klf1 and Scl, to recruit the histone variant H2A.Z, thereby regulating chromatin assembly and shaping enhancer landscape to activate the transcription of key erythropoiesis and epigenetic genes, such as *klf6a*, *kdm6bb, kmt2c*. The histone demethylase KDM6B removes the H3K27me3 marks.^[^ ^32^, ^57^ ^]^ Both KDM6B and KMT2C are essential for hematopoiesis,^[^ ^58^, ^59^ ^]^ although their specific roles in erythropoiesis remain unknown. Our findings highlight the critical and novel functions of *nap1l4a* in synergistically cooperating with erythropoiesis factors to establish an intricate regulatory network governing erythroid differentiation. Moreover, they delineate a previously unrecognized role of *nap1l4a* in orchestrating epigenetic regulation during primitive erythropoiesis through remodeling histone chromatin.

Here, we demonstrate that Nap1l4a functionally interacts with H2A.Z‐containing nucleosomes. Previous studies have reported that NAP1L4 interacts with P300,^[^ ^33^ ^]^ which is required for H3K27ac deposition and the promotion of chromatin accessibility.^[^ ^15^ ^]^ Consistently, we reveal that *nap1l4a* deficiency alters the enhancer landscape of erythrocyte TFs and epigenetic regulators, including *kdm6bb* and *kmt2c*. Moreover, the interaction between Nap1l4a and H2A.Z is required to maintain enhancer landscape and transcriptional activation of WNT signaling genes, a pathway known to regulate erythropoiesis in zebrafish.^[^ ^46^, ^60^ ^]^ Taken together, our results indicate that Nap1l4a cooperates with H2A.Z to regulate chromatin accessibility, thereby enabling selective gene induction in the erythrocyte lineage. These findings also uncover a previously unrecognized role for Nap1l4a as a modulator of WNT signaling during erythropoiesis.

Additionally, this study for the first time reveals that H3K27me3 enrichment is markedly reduced at the chromatin of adult hemoglobin genes (*hbaa2*, *hbaa1*, *hbba2*, and *hbba1*) in *nap1l4a* ^−/−^ mutants. These findings suggest that *nap1l4a* may play a critical role in primitive erythropoiesis by suppressing the premature onset of definitive erythropoiesis or the transition from primitive to definitive erythropoiesis, analogous to the function of Bcl11a in erythroid development.^[^ ^42^, ^61^ ^]^ This provides novel mechanistic insights into the regulation of erythroid development.

In this study, we observe that less than 1% of the binding loci of erythrocyte TFs Scl and Klf1, exhibit reduced occupancy in *nap1l4a* ^−/−^ mutants, whereas over 10% exhibit increased occupancy. Notably, the loci with decreased binding are primarily associated with genes involved in primitive erythropoiesis. Erythropoiesis is a complex process encompassing TFs and globin chain production, heme synthesis and transport, cell membrane and cytoskeleton production and iron procurement.^[^ ^62^, ^63^, ^64^, ^65^ ^]^ Klf1 functions broadly across nearly all stages of erythropoiesis, whereas Scl also participates in blood vessel development and neurogenesis, in addition to its essential roles in primitive and definitive hematopoiesis.^[^ ^66^, ^67^, ^68^ ^]^ We observed a reduction in primitive erythrocytes and an expansion of the vasculature in *nap1l4a^−/−^* mutants. These results suggest that Nap1l4a is crucial not only for regulating key erythropoiesis genes, but also for restraining the roles of Scl in promoting vessel cell fate, reminiscent of the function of AP‐1 in balancing cell fate between vascular smooth muscle and hemogenic cell fate.^[^ ^69^ ^]^ Furthermore, motif analysis using the HOMER tool suggests that co‐occupy of Nap1l4a with AP‐1, however, whether Nap1l4a co‐operates with AP‐1 during organogenesis remains to be elucidated.

This study provides objective evidences for the critical role of Nap1l4a in regulating zebrafish erythropoiesis through transcriptional regulation and chromatin remodeling in a developmentally stage‐specific manner (Graphical Abstract, made in BioRender.com). However, the mechanism by which Nap1l4a facilitates H2A.Z deposition into nucleosomes remains unclear. Interestingly, we found that Nap1l4a may interact with the chromatin remodeler Smarca1, and recent studies indicate that SWI/SNF complexes play key roles in poising the T cell effector landscape.^[^ ^70^ ^]^ Collectively, these findings have broad implications for understanding human anemia and may provide valuable insights into economically or physiologically relevant traits in fish, such as disease resistance and hypoxia tolerance.^[^ ^71^ ^]^

## 4 Conclusion

Collectively, our results reveal a novel role for Nap1l4a in primitive erythropoiesis by orchestrating chromatin remodeling and transcriptional regulation, highlighting its potential relevance to human hematological disorders. This study underscores the critical function of *nap1l4a* in vertebrate erythropoiesis and provides new mechanistic insights into the molecular mechanisms regulation of this process.

## 5 Experimental Section

**Scheme 1:**
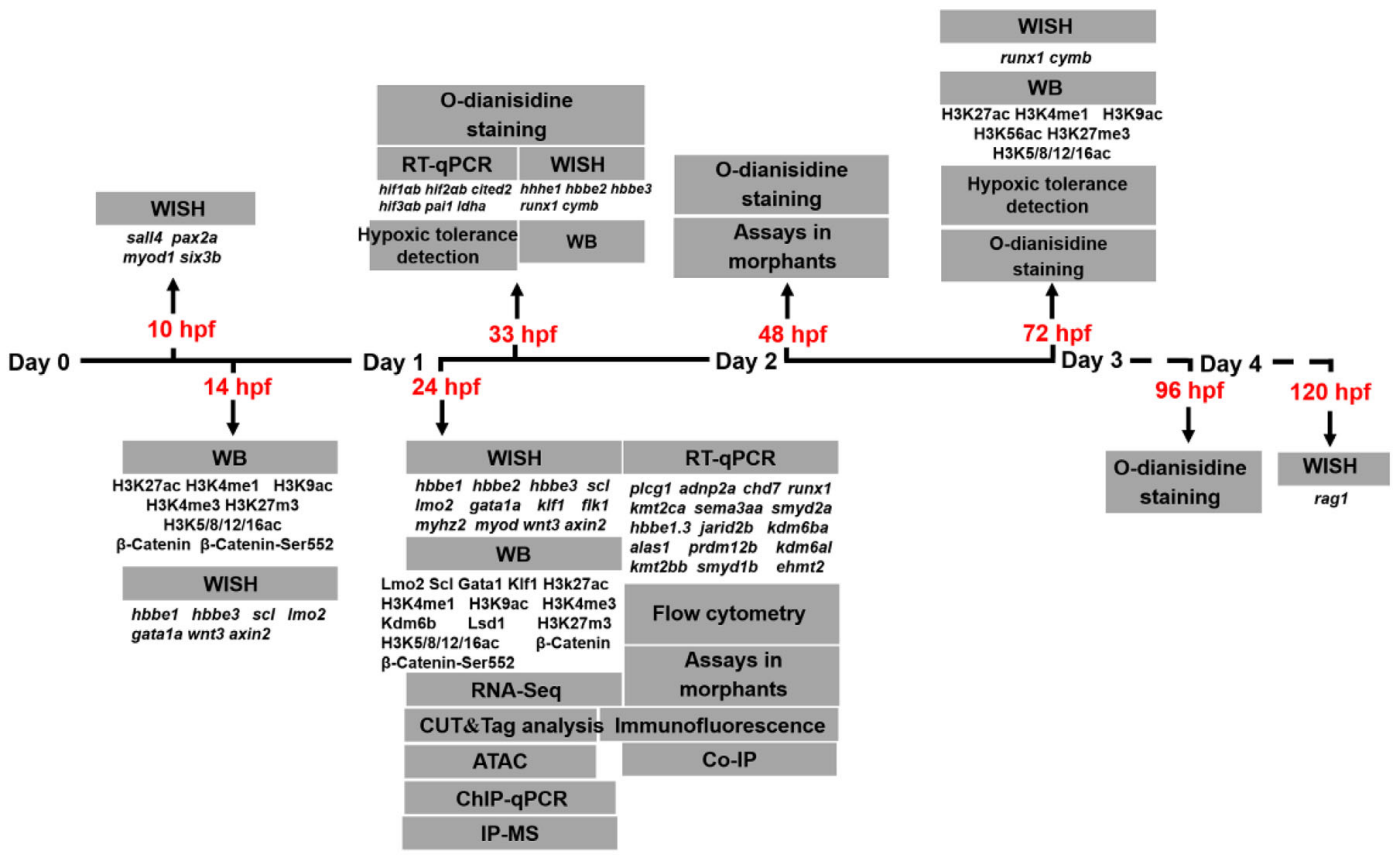
Experimental performance and sample collection in this study.

The full names and abbreviations of genes tested in this study are listed in Table S16 (Supporting Information). The sample collections and the following experiments were performed as indicated in **Scheme** **1**.

### Maintenance of Zebrafish Stocks and Generation of Zebrafish Mutants using CRISPR/Cas9

In this study, *nap1l4a* ^−/−^ (‐5bp deletion) mutants were generated via the CRISPR/Cas9 system, using the following guide RNA (gRNA) targeting sequence: 5′‐ TGCTGTCGAACCTACAGATGAGG‐ 3′. The gRNA was synthesized using T7 RNA polymerase (Cat# K0441, Invitrogen, USA). The mixture of gRNA (500 ng/µL) and Cas9 mRNA (600 ng/µL) was co‐injected into one‐cell stage embryos. Next, the culture of the injected embryos, the following screening and culture of F0 generation, F1 heterozygous, and F2 homozygous line, respectively, were performed as reported recently.^[^ ^7^, ^46^, ^72^ ^]^ Genotyping assays for *nap1l4a* heterozygote (*nap1l4a* ^+/−^) and homozygous mutants (*nap1l4a* ^−/−^) were performed using the primers listed in Table S17 (Supporting Information). The phenotype of *nap1l4a* ^−/−^ embryos or larvae, were observed and photographed using a stereoscopic microscope (Leica, M205FA, Germany). All zebrafish maintenance and experiments were conducted in accordance with the Guidelines for Experimental Animals approved by the Institutional Animal Care and Use Ethics Committee of Huazhong Agricultural University (permit number HZAUFI‐2016‐007).

### Drug Treatment

Bio (6‐Bromoindirubin‐3′‐oxime) (B1686, Sigma‐Aldrich) was prepared as described previously.^[^ ^73^ ^]^ GSK‐J1 (HY‐15648, MedChemExpress) was dissolved in DMSO (D2650, Biosharp). Embryos from the control and *nap1l4a* ^−/−^ mutants at bud stage were exposed to BIO (0.05 µm) or GSK‐J1 (10 µm), respectively, and were harvested at the indicated stages. Biological replicates were performed three times with over 20 embryos per group one time.^[^ ^7^ ^]^

### Quantitative Real‐Time PCR (qRT‐PCR)

To determine the transcriptional expression of *nap1l* family genes, expression of hypoxia responsive genes *hif1αb*, *hif2αb*, *hif3α*, *cited2*, *pai1* and *ldha*, and transcriptional expression of Nap1l4a targets, qRT–PCR was conducted in this study as reported previously.^[^ ^74^ ^]^ The primer sequences were listed in Table S18 (Supporting Information), and the primer sequences of genes *cited2*, *pai1*, and *ldha* have been reported recently.^[^ ^46^ ^]^ Each sample was repeated at least three times. Differences were calculated by the HCt comparative quantification method using *β‐actin* or *18s* as an internal control.

### Hypoxia Treatment

In this study, the hypoxia treatment was performed as reported recently.^[^ ^46^ ^]^ Briefly, the InvivO_2_ 300 Hypoxia Workstation was used for hypoxia treatment of zebrafish embryos and larvae (33 hpf and 72 hpf), with the O_2_ concentration adjusted to the appropriate value (2% for the embryos and larvae) before the experiments. *nap1l4a* ^−/−^ and WT control zebrafish larvae were placed into a 60 mm cell culture dish filled with 5 mL of water in the workstation, with 30 larvae per dish. Each experiment was repeated three times. Meanwhile, *nap1l4a* ^−/−^ and WT control embryos or larvae exposed to normoxia (21% O_2_) were used for the controls.

### O‐Dianisidine Staining

To detect the hemoglobin level in living zebrafish embryos and larvae, O‐dianisidine (D9143, Sigma–Aldrich) staining was used to indicate hemoglobin in the WT control and *nap1l4a* ^−/−^ embryos or larvae at 36, 48, 72, and 96 hpf (hours post fertilization), respectively, and the experiments were performed as reported previously.^[^ ^46^ ^]^ After staining, a rust‐colored precipitate (labeled hemoglobin) appeared specifically in erythrocytes, and the treated embryos or larvae with a lighter colored precipitate were defined as embryos or larvae with reduced erythrocytes (hemoglobin). Next, the embryos or larvae were transferred to 100% glycerol for stereoscopic observation and photographs (Leica, M250FA, Germany), followed by calculating the percentage of embryos or larvae with reduced hemoglobin as mentioned above and reported previously.^[^ ^46^ ^]^

### Morpholino (MO) Injection

The morpholinos (MOs) of *nap1l4a*, *p53*, *kmt2ca*, *kmt2cb* and *kdm6bb*, were purchased from Gene Tools LLC (Philomath, Oregon, USA) and dissolved in ddH_2_O at 3 mm concentration (stock solution). The sequences of MOs are listed in Table S19 (Supporting Information). In all experiments, the MOs were injected into one‐cell stage embryos, with the MO doses of *nap1l4a*, *p53*, *kmt2ca*, *kmt2cb* and *kdm6bb* at 0.6 mm, respectively. Meanwhile, 0.3 mm *kmt2ca* and 0.3 mm *kmt2cb* were together used in the two MOs injected embryos. 0.15 mm *kmt2ca* and 0.15 mm *kmt2cb* and 0.3 mm *kdm6bb* were together used in the three MOs injected embryos.

### Whole‐Mount In Situ Hybridization (WISH)

WISH detection followed the procedure reported recently.^[^ ^75^ ^]^ In this study, the following genes were tested: *hbbe1* (hemoglobin beta embryonic‐1.1), *hbbe2* (hemoglobin beta embryonic‐2), *hbbe3* (hemoglobin beta embryonic‐3), *tal*/*scl* (T‐cell acute lymphocytic leukemia 1), *lmo2* (LIM domain only 2), *gata1a* (GATA binding protein 1a), *klf1* (Kruppel like factor 1), *sall4* (spalt‐like transcription factor 4), *pax2a* (paired box 2a), *myod* (myogenic differentiation 1), *six3b* (SIX homeobox 3b), *flk1* (kinase insert domain receptor like), *cmyb* (v‐myb avian myeloblastosis viral oncogene homolog), *runx1* (RUNX family transcription factor 1), *rag1* (recombination activating 1), *wnt3* (wingless‐type MMTV integration site family, member 3), *axin2* (conductin, axil). The probes for the genes were reported in the papers recently,^[^ ^76^, ^77^ ^]^ and some probes, such as *klf1*, were amplified using the primers displayed in Table S20 (Supporting Information). WISH embryos or larvae in each group were observed and photographed using a stereoscopic microscope (Leica, M250FA, Germany). For WISH data, the number in the right‐down corner in each panel in WISH figures was shown as N_changed_/N_total_, where N_changed_ indicates the number of embryos with reduced or increased expression, and N_total_ indicates the total number of embryos in a group.

### Western Blotting

For Western blotting (WB) analysis, total protein was extracted from embryos or larvae (30–50 embryos/sample) using lysis buffer (10 mm Tris–HCl, 10% glycerol, 1% SDS, 1% Chaps) with Protease Inhibitor (P2714, Roche, 1 µL for 1 mL) on ice for 30 min, followed by centrifugation at 20 000 g for 10 min to clear the cell lysate. The protein concentration was measured using the BCA protein assay kit (BioRad, American). Then, each sample was added with SDS‐loading buffer and boiled for 10 min. Next, the samples were loaded in 10% SDS‐PAGE gel for electrophoretic separation. Proteins were transferred onto a polyvinylidene difluoride (PVDF) membrane (Millipore, American), probed with primary antibodies, and detected with horseradish peroxidase‐conjugated secondary antibodies (BL033A, Biosharp). The following antibodies were used in the assays: Nap1l4 (A15238, ABclonal), HIF‐1a (A7553, ABclonal), Tal1/Scl (Cat No:55317‐1‐AP, Proteintech), Lmo2 (Cat No: 21966‐1‐AP, Proteintech), Gata1 (Cat No: 10917‐2‐AP, Proteintech), Klf1 (A24503, ABclonal), β‐Catenin (AF8340, Affinity), Phospho‐β‐Catenin‐S552 (AP0979, ABclonal), Actin (AC026, ABclonal), H3K4me1 (A2355, ABclonal), H3K4me3 (A2357, ABclonal), H3K27me3 (A2363, ABclonal), H3K27ac (A7253, ABclonal), H3K9ac (A7255, ABclonal), H3K56ac (A7256, ABclonal), H3K5/8/12/16 (A20764, ABclonal), anti‐H3 (A2348, ABclonal), Kdm6B (Cat No. 55354‐1‐AP, Proteintech), Lsd1 (A21801, ABclonal), H2A.Z (Cat No:16441‐1‐AP, Proteintech), HRP‐conjugated Goat anti‐Rabbit IgG (H+L) (AS014, ABclonal), HRP‐conjugated Mouse anti‐Rabbit IgG Light Chain (AS061, ABclonal), HRP‐conjugated Goat anti‐Rabbit IgG Heavy Chain (AS063, ABclonal). The antibody probed membranes were stained using the ECL‐Plus Kit (P0018FS, Beyotime), then, the blots were photographed using a LAS4000 mini luminescent image analyzer. ImageJ was used for quantifying the protein levels based on the band density obtained in the WB analysis.

### EdU (5‐Ethynyl‐2′‐deoxyuridine) Labeling and Immunofluorescence

EdU labeling was performed based on the previously reported methods.^[^ ^78^ ^]^ Briefly, EdU (10 mm, Abbkine, KTA2031) was injected into Tg (*gata1a*: DsRed) and Tg (*nap1l4a^−/−^*; *gata1a*: DsRed) embryos at 22 hpf, respectively, and incubated for 2 h at 28 °C. Subsequently, the embryos were fixed in 4% paraformaldehyde (PFA) and preserved overnight at 4 °C. Next, the fixed embryos were permeabilized and stained using the EdU Cell Proliferation Image Kit (Orange Fluorescence) (KTA2031, Abbkine) in accordance with the manufacturer's protocol. Finally, the *gata1a*‐positive cells were stained using anti‐RFP primary antibody (5F8, Proteintech) and FITC‐conjugated goat anti‐rat IgG (H+L) secondary antibody (SA00003‐11, Proteintech).

Immunofluorescence of whole‐mount zebrafish embryos was performed as reported recently.^[^ ^79^ ^]^ *Tg* (*drl*: GFP) embryos at 24 hpf, injected with or without *nap1l4a* MOs, and *Tg* (*nap1l4a^−/−^*; *drl*: GFP) with their control, respectively, were collected and fixed in 4% paraformaldehyde overnight, and permeabilized with 1 mg/mL collagenase (AC15L141, Life‐iLab Biotech, China) for 25 min, and then blocking in 5% BSA for 1 h. Next, the embryos were incubated with anti‐GFP (AE011, ABclonal Technology, anti‐mouse) (to indicate *drl^+^* erythrocytes) and anti‐H3K27me3 (A2363, ABclonal Technology, anti‐rabbit), or anti‐H3K4me1 (A2355, ABclonal Technology, anti‐rabbit) overnight at 4 °C, respectively. After washing with PBST, the embryos were incubated with Alexa Fluor 555‐conjugated anti‐mouse (AS057, ABclonal Technology) and FITC‐conjugated anti‐rabbit antibodies (BL033A, Biosharp, China), respectively. Then, the immunofluorescence staining embryos were observed and photographed.

### Confocal Microscopy

For in vivo observation, the embryos from *Tg* (*drl*: GFP) and *Tg* (*LCR*: GFP) embryos at 24 hpf and *Tg* (*gata1a*: DsRed) at 48 hpf, injected with or without *nap1l4a* MOs, and *Tg* (*nap1l4a^−/−^*; *drl*: GFP) and *Tg* (*nap1l4a^−/−^*; *gata1a*: DsRed) with their control, were anesthetized using 0.168 mg mL^−1^ tricaine (Sigma–Aldrich, USA), followed by observation and photographing under a confocal microscope (Leica M205FA, Germany). The number of *drl*GFP^+^ or *gata1a*DsRed^+^ cells in *nap1l4a* morphants or *nap1l4a^−/−^* were counted, and the fluorescence of *drl*GFP^+^ or *gata1a*DsRed^+^ cells were calculated.

Immunofluorescence signals in whole embryos, H3K27me3 or H3K4me1 positive signaling in *drl*GFP^+^ at 24 hpf were scanned using a super resolution microscope (STORM‐A1R) in the State Key Laboratory of Agricultural Microbiology of Huazhong Agricultural University (Wuhan, China). The images were processed and quantified using NIS‐Elements Viewer 4.50 (Nikon, Japan).

### Flow Cytometry Analysis and Cell Apoptosis Assays

The DsRed‐positive cells in *Tg* (*gata1a*: DsRed) (control) and *Tg* (*nap1l4a* ^−/−^; *gata1a*: DsRed) embryos, and the GFP‐positive cells in *Tg* (*LCR*: GFP) and *Tg* (*drl*: GFP) (control) embryos injected with or without *nap1l4a* MOs at 24 hpf, were collected and homogelized. These cells were then analyzed by flow cytometry (BD FacsAria SORP 650110M3 BioDot, USA), and the percentages of *LCR*GFP^+^ and *drl*GFP^+^ cells in different groups were calculated.

Flow cytometry analysis was conducted on adult whole kidney marrow (WKM) cells from WT control and *nap1l4a* ^−/−^ zebrafish at 3 months post fertilization (mpf) as described previously.^[^ ^46^ ^]^ The samples were analyzed based on forward and side scatter characteristics, propidium iodide exclusion using CytoFLEX Flow Cytometer (Beckman Coulter, USA), and the flow cytometry data were analyzed with FlowJo software.

The early apoptosis and late apoptosis of zebrafish erythrocytes were detected using Annexin V‐PE Apoptosis Detection Kit (Cat# C1065S, Beyotime, China) and One‐step TUNEL Apoptosis Assay Kit Cat#KTA2031, Abbkine, China), respectively. The embryos from *Tg* (*drl*: GFP) injected with or without *nap1l4a* MOs, and *Tg* (*gata1a*: DsRed) and *Tg* (*nap1l4a* ^−/−^; *gata1a*: DsRed) at 24 hpf, were harvested and dissociated separately into single cells in phosphate‐buffered saline (PBS). Next, the cells from the Tg (*drl*: GFP) embryos were co‐stained separately with Annexin V‐PE and DAPI as instructed for the Annexin V‐PE Apoptosis Detection Kit, and the cells from *Tg* (*gata1a*: DsRed) embryos were co‐stained with TUNEL‐FITC and DAPI as instructed for the One‐step TUNEL Apoptosis Assay Kit, followed by analyzing Annexin V‐PE apoptosis in GFP‐positive cells, and TUNEL‐FITC apoptosis in DsRed positive cells, respectively, using flow cytometry analysis (CytoFLEX S, Beckman Coulter, USA) as performed recently.^[^ ^80^, ^81^ ^]^

### One Step Cell‐Direct qRT‐PCR

One step cell‐direct qRT‐PCR was performed as reported recently.^[^ ^80^ ^]^ Briefly, the Tg(*drl*: GFP) embryos injected with or without *nap1l4a* MOs at 24 hpf, were collected and homogenized, followed by sorting the different cell groups, *drl* ^+^ cells from WT control or *nap1l4a* MO injected embryos, respectively, into the lysate of the CellsDirect One‐Step qRT‐PCR Kit (Thermo Fisher Scientific, Cat#11753‐100) by FACS (BD FacsAria SORP, 650110M3, BioDot, American). Primer sequences for the tested genes are shown in Table S17 (Supporting Information), including zebrafish genes (*gata1a*, *scl*, *lmo2*, *klf1*, *hbbe1*, *hbbe2*, *hbbe3*). The qPCR data were analyzed as described above, and each sample was biologically repeated at least three times. Differences were calculated by the ΔΔCt comparative quantization method using *18s* as an internal control.

### Luciferase Reporter Assays

Luciferase reporter assays were performed as reported recently. 8×TopFlash reporter (25 ng/µL) and pTK‐renilla (5 ng/µL) were co‐injected into one‐cell stage*nap1l4a* ^−/−^ and WT control embryos respectively. *nap1l4a* ^−/−^ and WT control embryos at 14 hpf and 24 hpf were homogenized, respectively, then, the homogenates were separately measured using the Dual‐luciferase Reporter Assay System (DL101, Vazyme) following the protocol of the manufacturer to indicate the luciferase activities of 8×TopFlash reporter in different group respectively. The data were reported as the mean ± SD of three independent experiments in triplicate.^[^ ^80^ ^]^

### Transcriptome Sequencing

cDNA library construction and subsequent RNA sequencing (RNA‐Seq) were performed on the BGISEQ‐500 and Illumina NovaSeq 5000 platforms, respectively. Each sample was performed with at least two biological replicates. Sequencing reads were mapped to the GRCz11 reference genome using HISAT2 (v2.1.0). Gene expression counts were quantified using FeatureCounts (v1.6.0) with the current Ensemble annotation. Differential gene expressions were analyzed using the R package DESeq2, followed by Gene Ontology (GO) and Kyoto Encyclopedia of Genes and Genomes (KEGG) enrichment analyses with the ClusterProfiler (v 4.8.1) R package. TIGR Multi‐Experiment Viewer (MeV) was used for hierarchical clustering analysis to generate different volcano plot to show the expression of DEGs among different samples.

### ATAC‐Seq Sequencing

Raw sequencing data were filtered and quality controlled using fastp (v1.0.1) to remove low‐quality reads and trim adaptor‐contaminated sequences, yielding high‐quality clean reads for downstream analysis. The cleaned reads were aligned to the zebrafish (*Danio rerio*) reference genome (GRCz11) using Bowtie2 (v2.5.1) with default parameters. Reads mapped to the mitochondrial genome (for plants: reads mapped to both mitochondrial and chloroplast genomes) were filtered out using in‐house scripts. The resulting alignment files were processed with Sambamba (v0.7.1) for SAM/BAM format conversion and PCR duplicate removal.

To assess the correlation between samples, the genome was divided into 10 kb bins using multiBamSummary (v3.5.1). Read counts within each bin were normalized, and pairwise correlations among samples were then calculated. Peaks were identified using MACS2 (v2.2.9.1), and reproducibility between replicates was evaluated using IDR (v2.0.3).

### CUT&Tag Sequencing

WT control and *nap1l4a* ^−/−^ embryos at 24 hpf (50 embryos per sample) were homogenized in 1×PBS (50% FBS), and cells subsequently collected from dechorionated embryos. Cut & Tag (Vazyme, TD903) was performed on 10^5^ cells per sample from 24 hpf WT control and *nap1l4a* ^−/−^ embryos, using antibodies against Nap1l4, H3K4me1, H3K27me3, or H3K27ac, Scl, Klf1, or H2A.Z, respectively. Each experiment was performed with at least two biological replicates. CUT&Tag libraries were sequenced on the Illumina NovaSeq 6000.

The data processing and analysis included several steps. Initially raw sequencing data were quality controlled and filtered using fastp (v1.0.1), yielding high‐quality reads for downstream analysis. The cleaned reads were aligned to the zebrafish (*Danio rerio*) reference genome (GRCz11) using Bowtie2 (v2.5.1). Alignment files were processed with samtools (v1.6) to convert SAM to BAM format, and low‐quality alignments (Q < 30) were filtered out to ensure data reliability. To eliminate PCR duplicates, Picard (v2.25.2) was used to mark and remove duplicates from the BAM files. Subsequently, bamCoverage from Deeptools (v3.5.5) was employed to generate normalized signal intensity tracks from the BAM files. Peaks were identified using MACS2 (v 2.2.9.1) and differential peaks across experimental conditions were detected with the bdgdiff module. The resulting peaks were annotated with ChIPseeker (v1.36.0), followed by GO and KEGG enrichment analyses using ClusterProfiler (v 4.8.1) R package.

Representative snapshots of CUT&Tag tracks and transcriptome tracks were visualized using the IGV genome browser (v2.17.4). Motif analysis was performed with Homer software (v4.11). Enriched motif sequences were identified within these peak regions using findMotifsGenome.pl (Homer) against the GRCz11 reference genome. Enrichment chord diagrams were generated using the circlize (v0.4.15) R package. Enrichment results, including significant terms and their associated genes, were used to visualize connections between functional categories and genes.

### ChIP‑qPCR Assays

Chromatin immunoprecipitation qPCR (ChIP‐qPCR) assays were performed as reported recently.^[^ ^75^ ^]^ The chorions of *nap1l4a* ^−/−^ and WT control embryos (50 embryos/sample) at 24 hpf, respectively, were removed separately by pronase, followed by collecting the cross‐linked cells from the dechorinated eggs. The cells were washed twice with 1xPBS, and were lysed and then the precipitation of the cells was obtained by centrifugation, followed by successive treatment for 10 min in lysis buffer 1 (50 mm HEPES‐KOH pH 7.5, 140 mm NaCl, 1 mm EDTA, 10% glycerol, 0.5% NP‐40, 0.25% Triton X‐100) and lysis buffer 2 (10 mm Tris‐HCl pH 8.0, 200 mm NaCl, 1 mm EDTA, 0.5 mm EGTA) separately. Next, the pellet was suspended in 1 mL nucleus lysis buffer 3 (10 mm Tris‐HCl pH 8, 100 mm NaCl, 1 mm EDTA, 0.5 mm EGTA, 0.1% Na‐Deoxycholate, 0.5% N‐lauroylsarcosine), followed by sonication to obtain ≈200–500 bp chromatin DNA fragments.

After sonication, the input control, TCF4 (A1141, ABclonal Technology), H3K27me3, H3K4me1, H3K27ac, Nap1l4, H2A.Z, Scl, Klf1, and IgG (A7016, Beyotime) ChIP groups were treated and proceeded as reported recently.^[^ ^46^, ^60^ ^]^ Finally, the ChIP DNA was recovered by phenol/chloroform/isoamylal‐cohol (25:24:1) extraction and precipitated by ethanol. The pellet was re‐suspended in sterile water and used as a template for qPCR. The tested genes and their primers used for ChIP‐qPCR are listed in Table S21 (Supporting Information), and qPCR and data analysis followed a recently reported method.^[^ ^75^, ^82^ ^]^

### Separation of Cytoplasmic and Nuclear Proteins

The separation of cytoplasm from nucleus was performed using hypotonic buffer according to the protocol, and then high‐salt buffer was used to allow the nuclear proteins to be released.^[^ ^83^ ^]^ Cells of 24 hpf embryos (50 embryo/sample) were collected and washed with pre‐cooled PBS. The collected cells were added with buffer A (10 mm Hepes, 3.2 mm MgCl2, 10 mm KCl) and placed on ice for 10 min. Then, the cells were centrifuged at 2500 rpm for 10 min at 4 °C and re‐suspended in Buffer A containing 0.1% NP‐40. After a 10‐min incubation on ice, cytoplasmic proteins were released, and the cytoplasm was separated from the nuclei by centrifugation at 5000 rpm for 10 min. The nuclei, retained in the pellet, were re‐suspended in Buffer B (20 mm Hepes, 0.42 m NaCl, 0.1 mm EDTA, and 25% glycerol) with 0.1% NP‐40. The suspension was incubated on ice for 40 min and then centrifuged at 12 000 rpm for 15 min to extract the nuclear proteins.

### Co‐IP (Co‐Immunoprecipitation) and IP‐MS (Immunoprecipitation‐Mass Spectrometry)

In this study, the immunoprecipitation followed by mass spectrometry (IP‐MS) was performed in this study. The cells collected from 24 hpf *nap1l4a* ^−/−^ and WT control embryos (50 embryos/sample), respectively, were lysed with Western & IP Lysis Buffer (Gbcbio Technologies, G3423) with PMSF (biosharp, BL507A‐P; 1:1000) and protease inhibitors (Roche, 4 693 159 001, 1:100). Cell extracts containing an equal amount of protein from both the WT control and *nap1l4a* ^−/−^ were incubated overnight at 4 °C with the indicated primary antibody (anti‐Nap1l4, dilution at 1:100). Protein A/G Magnetic Beads (HY‐K0202, MedChemExpress) (50 µL of 50% slurry) were then added, and the incubation was continued for an additional 1 h at room temperature. The magnetic beads were then collected by magnetic sorting (OSE‐MF‐01, Tiangen Biotech). The magnetic beads were used for immunoprecipitation‐mass spectrometry (IP‐MS) analysis in the following. Heatmaps for proteins from IP‐MS data were generated using the Deeptools software.

In Co‐IP assays, the magnetic beads were washed for three times using lysis buffer, followed by one wash with 1xPBS. Then, the co‐immunoprecipitated protein samples were subjected to SDS‐PAGE and the following WB assays. The primary antibodies used here included Nap1l4, H2A.Z, beta‐Catenin (8480, Cell Signaling Technology), Tal1/Scl, Klf1, and IgG (A7016, Beyotime), respectively.

### Statistical Analysis

The sample size exceeded 10 embryos (n > 10) for WISH and immunofluorescence, while at least 50 embryos in each group was used for RNA and protein extraction. Each assay included two to three biological replicates per group. Percentage analysis across groups were performed using the hypergeometric distribution in R. Signals from WISH, WB, and immunofluorescence images were quantified with ImageJ (NIH, Bethesda, Maryland), and analyzed by *t* test using GraphPad Prism 8.0. Each dot represents the signal level from a single embryo image within a group. qPCR data were analyzed by one‐way ANOVA followed by Tukey's post hoc test using IBM SPSS Statistics for Windows, Version 22 (Released 2013; IBM Corp., USA). Each dot represents a single replicate. Statistically significance was denoted as follows: *P* < 0.05 (*), *P* < 0.01 (**), and *P* < 0.001 (***). A threshold of ≥1.5‐fold change (increase/decrease, *P* ≤0.05) was used to define significant differences between groups.

## Conflict of Interest

The authors declare no conflict of interest.

## Author Contributions

J.S., F.L., and Z.S. contributed equally to this work. J.S., F.L., and Z.S. were responsible for writing the original draft, conducting experiments, data curation, formal analysis, methodology development, software implementation, validation, and visualization. J.S., F.L., Z.S., X.Y.Z., W.Y.L., and Y.W. contributed to experiments, formal analysis, methodology, validation, and visualization. G.L. and K.L. provided writing – review and editing. Y.P.F. and J.X.L. contributed through writing – review and editing, supervision, project administration, formal analysis, methodology, funding acquisition, and conceptualization. All authors have agreed on the contents of the manuscript.

## Supporting information



Supporting Information

Supporting Information

Supporting Information

## Data Availability

The raw CUT&Tag, ATAC‐Seq and RNA‐Seq sequence data generated in this study have been deposited into the Genome Sequence Archive at the National Genomics Data Center,^[^ ^83^ ^]^ China National Center for Bioinformation/Beijing Institute of Genomics, Chinese Academy of Sciences, under accession numbers: CRA022661(CUT&Tag data for Nap1l4a, H3K4me1, K3K27ac, H3K27me3, H2A.Z), CRA027566 (CUT&Tag data for Scl, Klf1), CRA033066 (ATAC‐Seq data), and CRA022407 (RNA‐Seq data), respectively. These data are publicly accessible at https:ngdc.cncb.ac.cn/gsa. The predicted P300 interacting proteins, including Nap1l4a, are obtained from GEO data, GSE229399.
