# Supplementary material for: Nap1L4a Cooperates with Scl/Klf1 to Recruit H2A.Z in Mediating Interactions Among Cis‐Regulatory Elements and Transcription Required for Primitive Erythropoiesis in Zebrafish
Source: Adv Sci (Weinh). 2025 Dec 12;13(12):e13762. doi: 10.1002/advs.202513762 (PMC12948261; doi:10.1002/advs.202513762)
Supplement: Supplementary file 1 — Supporting Information [file ADVS-13-e13762-s002.docx]

**Supporting information**


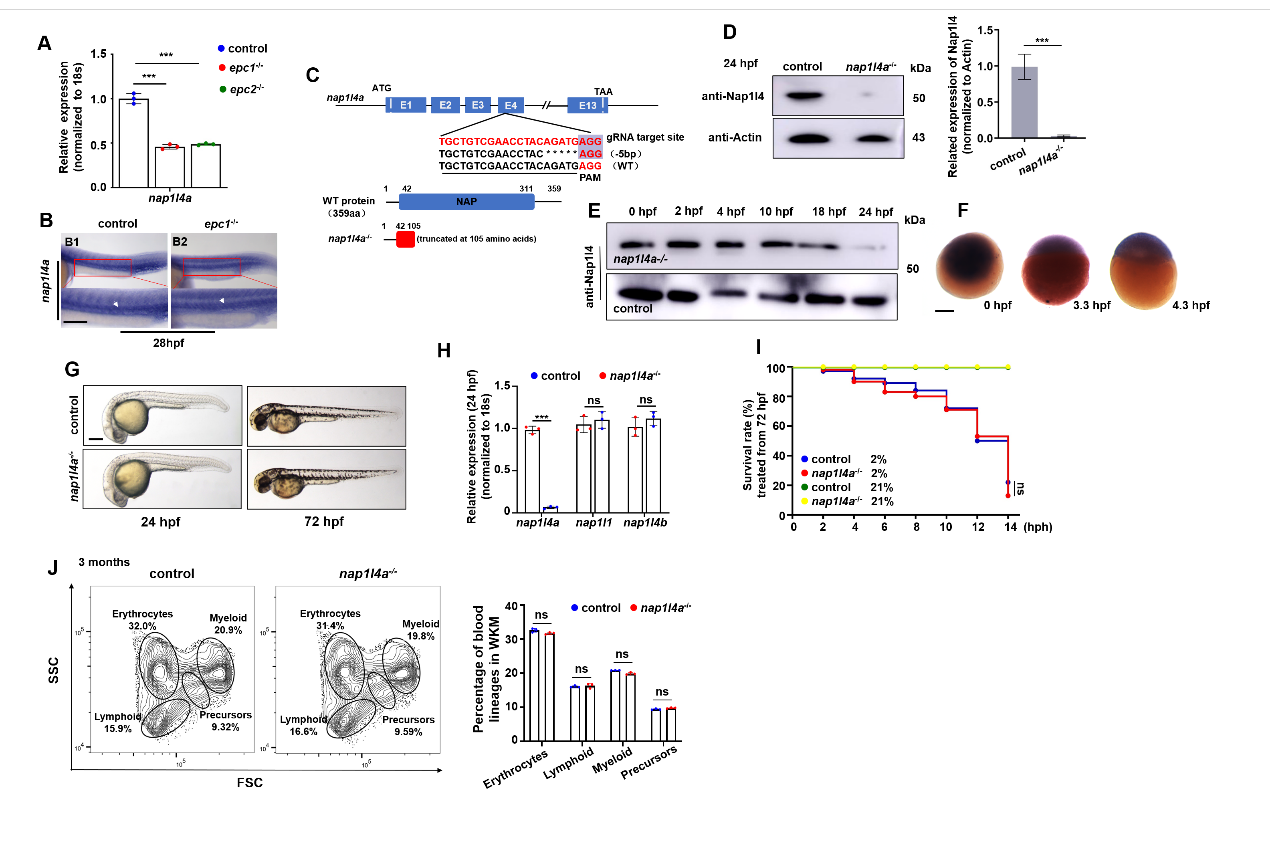


**Fig.S1 Spatially and temporally transcriptional expression of *nap1l4a* in embryogenesis and responses of *nap1l4a^-/-^* to hypoxia stress.**

(**A**) Transcriptional level of *nap1l4a* in *epc1^-/-^*, *epc2^-/-^* and in the wild type (WT) control embryos at 28 hpf, respectively. (**B**) Distribution of *nap1l4a* transcripts in *epc1^-/-^* and WT control embryos at 28 hpf. The red square and white arrows indicate the ICM region. (**C**) Schematic diagram showing the genomic structure and a genetic mutation of zebrafish *nap1l4a* gene. (**D**) Protein levels of Nap1l4 in *nap1l4a^-/-^* and WT at 24 hpf. (**E**) Protein levels of Nap1l4 in *nap1l4a^-/-^* and WT control from 0 hpf (hours post fertilization) to 24 hpf. (**F**) Transcriptional level of *nap1l4a* in WT embryos at early stages. (**G**) Phenotype of *nap1l4a*^-/-^ and WT embryos at 24 hpf and 72 hpf, respectively. (**H**) Transcriptional level of *nap1l4a*, *nap1l1*, and *nap1l4b* in *nap1l4a^-/-^* and WT respectively at 24 hpf. (**I**) Survival curves of *nap1l4a*^−/−^and WT embryos exposed to hypoxia (2% O_2_) beginning at 72 hpf for 14 h. (**J**) Proportion of different blood lineages in adult *nap1l4a*^−/−^ at 3 months (m). ImageJ was used for quantifying the protein levels. Each experiment was repeated at least three times, and a representative result is shown. Data were analyzed by *t* test using GraphPad Prism 8.0. Data are presented as mean ± SD. ****P* < 0.001, NS, not significant. Scale bar: 75μm in **B**, **F**, **G**.


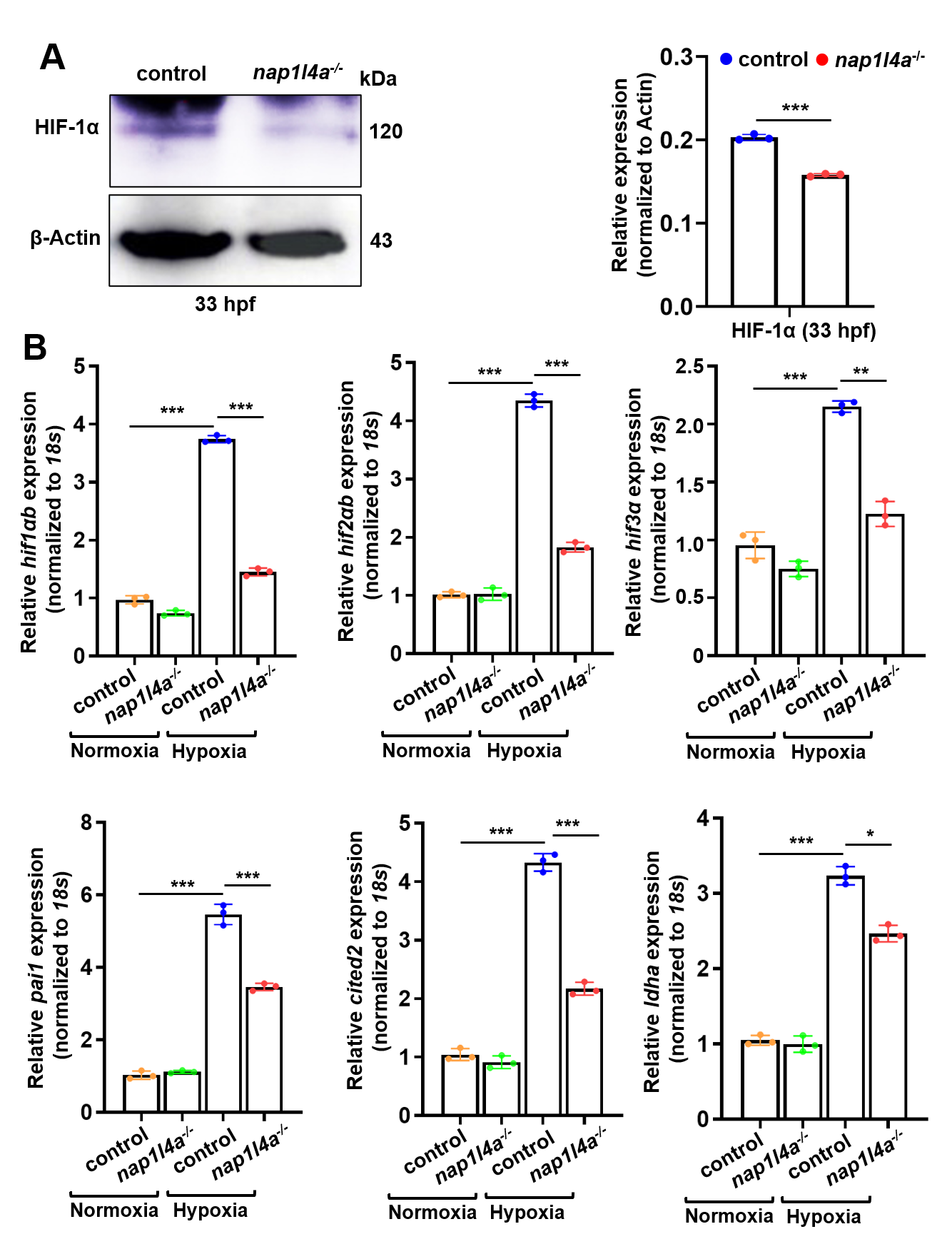


**Fig.S2 Effects of *nap1l4a* deficiency on the expressions of hypoxia inducible factor/genes** **in zebrafish embryos and larvae under hypoxia stress.** (**A**) Protein levels of HIF-1a in *nap1l4a*^-/-^ and WT embryos exposed to hypoxia (2% O_2_) beginning at 33 hpf for 14h, and graph shows the relative protein level in each sample. (**B**) qRT–PCR analysis of *hif1αb*, *hif2αb*, *hif3α*, *cited2*, *pai1* and *ldha* in *nap1l4a*^-/-^and WT control embryos exposed to hypoxia (2% O_2_), beginning at 33 hpf for 14h. Each experiment was repeated at least three times, and a representative result is shown. Data were analyzed by *t* test using GraphPad Prism 8.0. Data are presented as mean ± SD. **P* < 0.05, ***P* < 0.01, ****P* < 0.001.


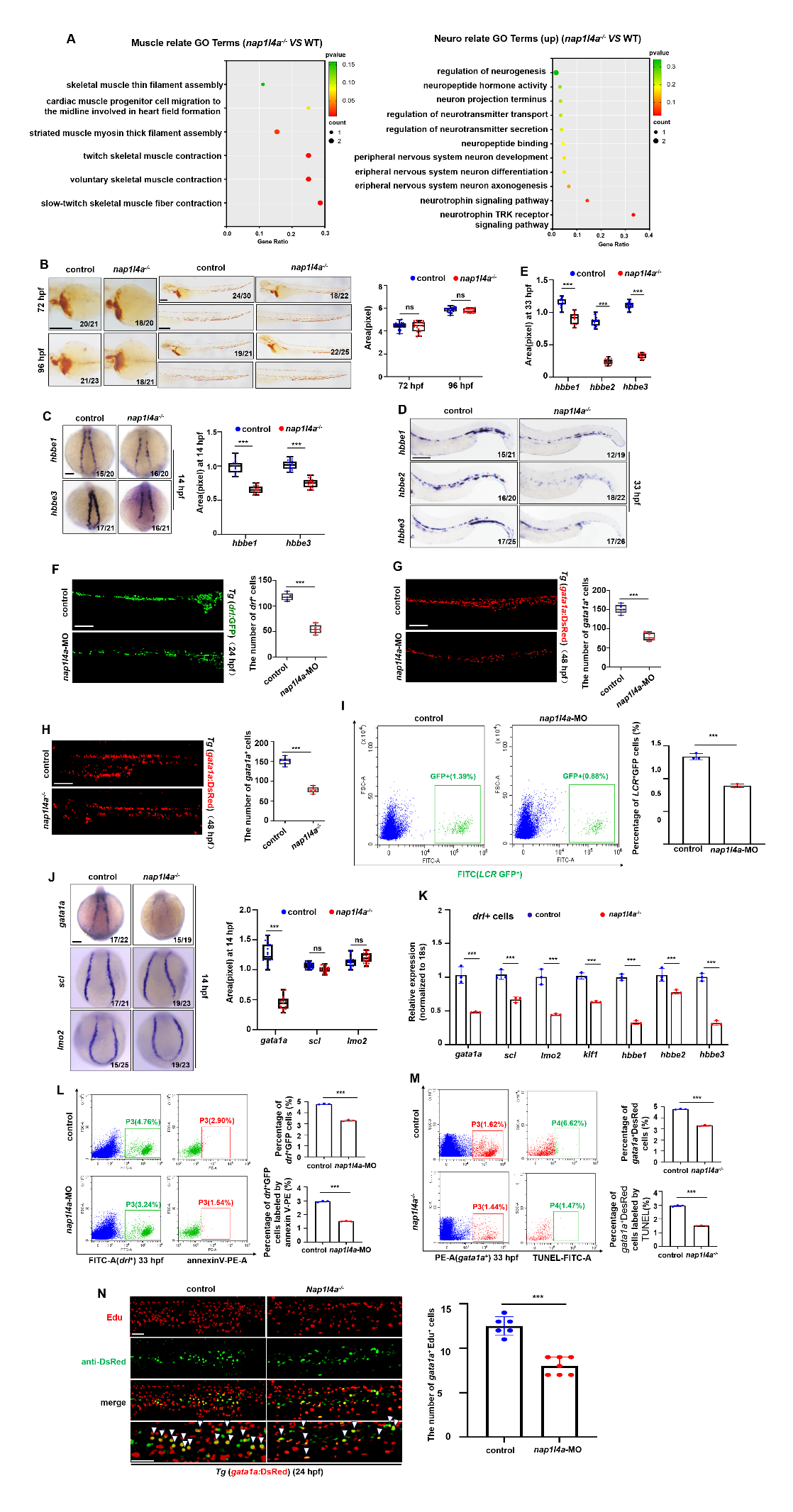


**Fig. S3** **Effects of *nap1l4a1* deficiency on erythrogenesis in zebrafish.** (**A**) Bubble plot showing muscle-related and neuro-related GO terms in *nap1l4a*^-/-^ relative to WT embryos at 24 hpf. (**B**) O-dianisidine staining of erythrocytes at 72 and 96 hpf in *nap1l4a*^-/-^ and WT embryos, with box plots show statistical analysis of O-dianisidine staining results. (**C**) WISH analysis of embryonic hemoglobin genes (*hbbe1* /*hbbe3*) in *nap1l4a*^-/-^ and WT control embryos at 14 hpf, with box and whisker plots showing relative transcriptional levels. (**D, E**) WISH analysis of embryonic hemoglobin genes (*hbbe1*/*hbbe2/hbbe3*) in *nap1l4a*^-/-^ and WT control embryos at 33 hpf, with box and whisker plots showing relative transcriptional levels (**E**). (**F**) Representative images of *Tg* (*drl*: GFP) embryos in *nap1l4a* MO and WT genetic background at 24 hpf, with box and whisker plots showing the number of *drl*^+^ cells. (**G**) Representative images of *Tg* (*gata1a*: DsRed) embryos in *nap1l4a* MO and WT genetic background at 48 hpf, with box and whisker plots showing the number of *gata1a*^+^ cells. (**H**) Representative images of *Tg* (*gata1a*: DsRed) embryos in *nap1l4a*^-/-^ and WT genetic background at 48 hpf, with box and whisker plots showing the number of *gata1a*^+^ cells. (**I**) Representative images of *Tg* (*LCR*: GFP) embryos in *nap1l4a* MO and WT genetic background at 24 hpf, with box and whisker plots showing the number of *LCR*^+^ cells. (**J**) WISH analysis of erythroid transcriptional factors (TFs) (*gata1a*, *scl*, and *lmo2*) in *nap1l4a*^-/-^ and WT control embryos at 14 hpf, with box and whisker plots showing their relative transcriptional levels. (**K**) **q**RT-PCR assays unveiled significantly down-regulated expression of erythroid genes in *drl*^+^ cells. (**L**, **M**) Flow cytometry analysis of erythrocytes and their progenitor, *drl^+^*(**L**) and *gata1a^+^*(**M**) in *nap1l4a* functional deficiency and WT control embryos at 33 hpf, with graphs showing the percentage of erythrocytes in each sample (**up**) and the percentage of apoptosis erythrocytes in each sample (**down**). (**N**) Proliferation assays for *gata1a^+^* cells in *nap1l4a*^-/-^ and WT control embryos at 24 hpf.  **B**, **D, E, F, G, H, M,** lateral view, anterior to the left, and dorsal to the up. Some panels in **B** are ventral view; **C**, **J**, dorsal view, anterior to the up. Each experiment was repeated at least three times, and a representative result is shown. Signals from WISH, WB, O-dianisidine staining, and immunofluorescence images were quantified with ImageJ. Data were analyzed by *t* test using GraphPad Prism 8.0. Data are presented as mean ± SD. ***P* < 0.01, ****P* < 0.001, NS, not significant. Scale bars: 75 μm in **B, C**, **D, E, F, G, H, J, N**.


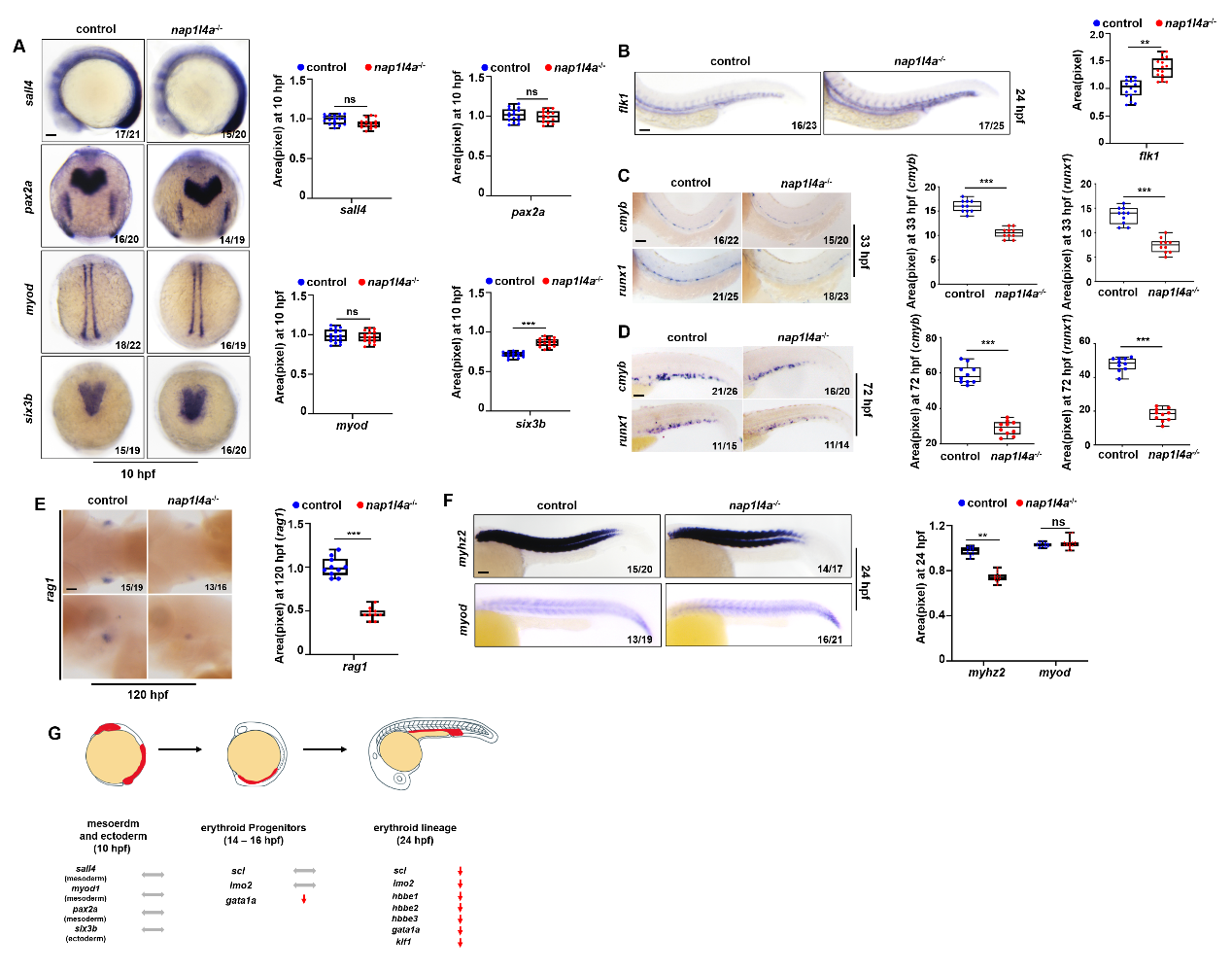


**Fig.S4 Effects of *nap1l4a1* deficiency on the expression of genes *myod*/*pax2a*/*six3b*/*sall4*/*flk1*/*runx1*/*cmyb*/*rag1*/*myhz2*.** (**A**) WISH analysis of mesodermal markers *sall4* and *pax2a*, mesoderm progenitor *myod*, and ectodermal marker *six3b* in *nap1l4a*^-/-^ and WT control embryos at 10 hpf, with box and whisker plots showing relative transcriptional levels. (**B**) WISH analysis of vascular marker *flk1* in *nap1l4a*^-/-^ and WT control embryos at 24 hpf, with box and whisker plots showing relative transcriptional levels. (**C**, **D**) WISH analysis of HSPC markers *runx1* and *c-myb* in *nap1l4a*^-/-^ and WT embryos at 33 hpf (**C**) and 72 hpf (**D**), with box and whisker plots showing relative transcriptional levels. (**E**) WISH analysis of lymphoid marker *rag1* in *nap1l4a*^-/-^ and WT embryos at 120 hpf, with box and whisker plots showing relative transcriptional levels. (**F**) WISH analysis of fast muscle-specific marker gene *myhz2* and myogenic regulatory factors *myod* in *nap1l4a*^-/-^ and WT embryos at 24 hpf, with box and whisker plots showing relative transcriptional levels. (**G**) Summary of gene expression changes in primitive erythrogenesis in *nap1l4a^-/-^* mutants. **A**, embryos are shown in dorsal view, anterior to the up. **B, C, D, E, F,** embryos and larvae are shown in lateral view, anterior to the left, and dorsal to the up. Each experiment was repeated at least three times, and a representative result is shown. Signals from WISH, images were quantified with ImageJ. Data were analyzed by *t* test using GraphPad Prism 8.0. Data are presented as mean ± SD. **P* < 0.05, ***P* < 0.01, ****P* < 0.001, NS, not significant. Scale bar: 75 μm in **A, E**, **B**, **C**, **D, F**.


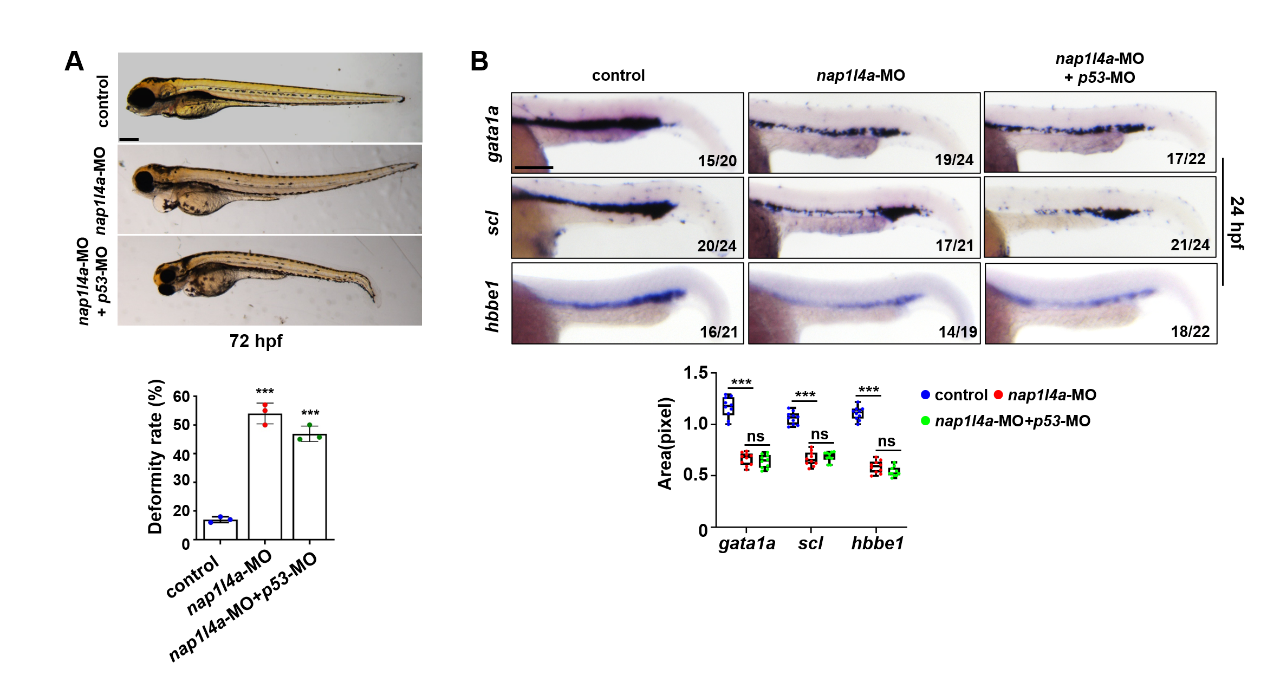


**Fig.S5** **Effects of *nap1l4a* MOs on erythropoiesis independent on *p53*.** (**A**) Phenotype of WT control, *nap1l4a* MO-injected, and *nap1l4a* MO plus *p53* MO-coinjected larvae at 72 dpf, and graphs show the deformity in different groups. (**B**) WISH analysis of *gata1a*, *scl* and *hbbe1* expression in the WT control, *nap1l4a* MO-injected, and *nap1l4a* MO plus *p53* MO-coinjected embryos at 24 hpf, with box and whisker plots showing the relative transcriptional levels. **A**, **B** lateral view, anterior to the left, and dorsal to the up. dpf, days post fertilization. Each experiment was repeated at least three times, and a representative result is shown. Signals from WISH images were quantified with ImageJ. Data were analyzed by *t* test using GraphPad Prism 8.0. Data are presented as mean ± SD. ****P* < 0.001, NS, not significant. Scale bars: 75 μm in **A** and **B**.


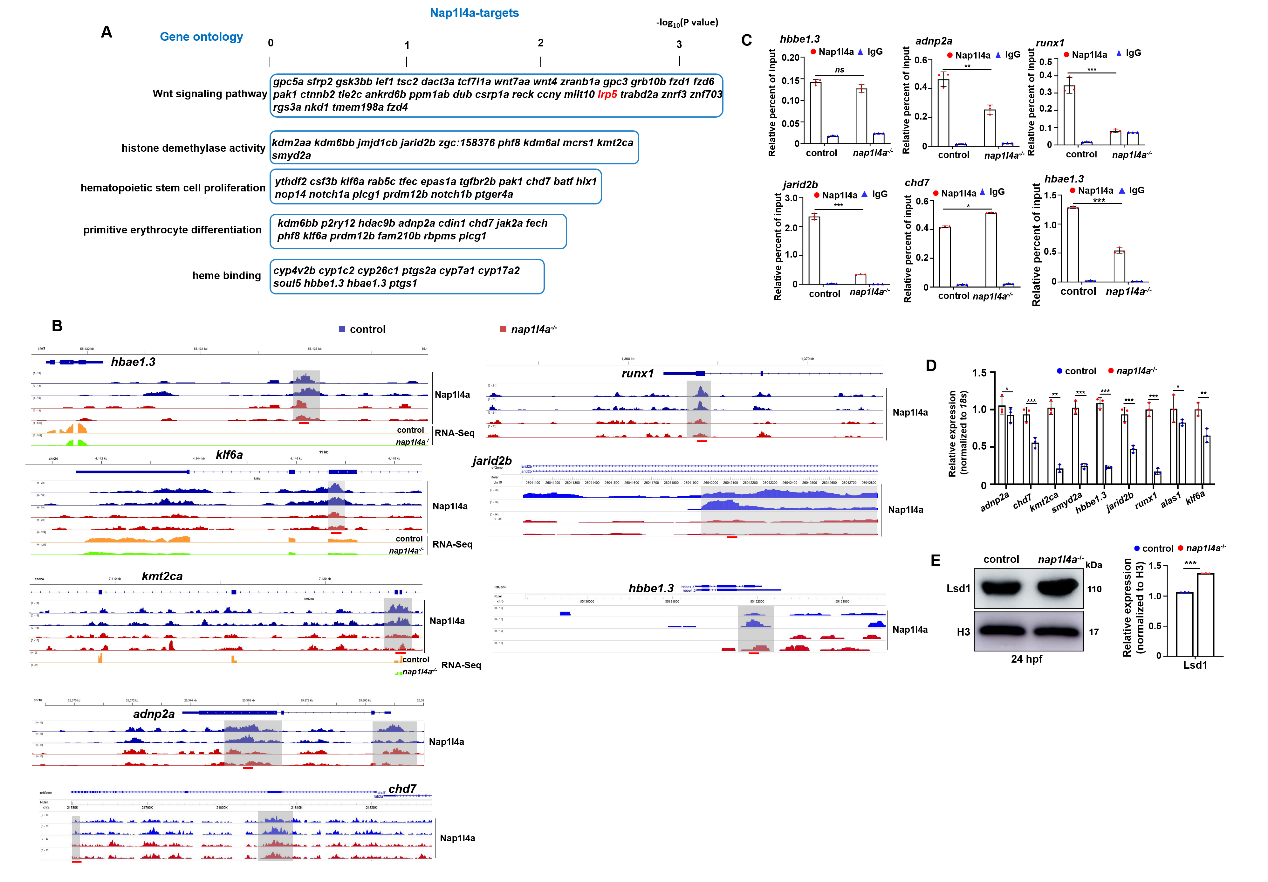


**Fig.S6 Nap1l4a targets genes in WNT signaling pathway, histone demethylase activity, primitive erythrocyte differentiation, and others.** (**A**) GO terms of Nap1l4a direct targets with integration of the RNA-seq and CUT&Tag data. (**B**) IGV browser tracks showing Nap1l4a binding profiles and corresponding targeted transcript levels for primitive erythrocyte differentiation genes (*adnp2a* and *klf6a*), heme binding genes (*hbae1.3* and *hbbe1.3*), HSPC and myeloid genes (*chd7* and *runx1*), as well as histone demethylase and methylase genes (*kmt2ca* and *jarid2b*). Gray-shaded regions highlight differential binding enrichment loci. (**C**) Enrichment of Nap1l4a binding (by ChIP-qPCR) on promoter or enhancer regions of targeted genes in WT control and *nap1l4a*^-/-^. (**D**) qRT-PCR assays for Nap1l4a targets in WT control and *nap1l4a* mutant at 24 hpf. (**E**) Protein levels of Lsd1 in *nap1l4a*^-/-^ and WT embryos at 24 hpf. Each experiment was repeated at least three times, and a representative result is shown. CUT&Tag tracks and transcriptome tracks were visualized using the IGV genome browser (v2.17.4). Signals WB images were quantified with ImageJ. Data were analyzed by *t* test using GraphPad Prism 8.0. Data are presented as mean ± SD. **P* < 0.05, ***P* < 0.01, ****P* < 0.001, NS, not significant.


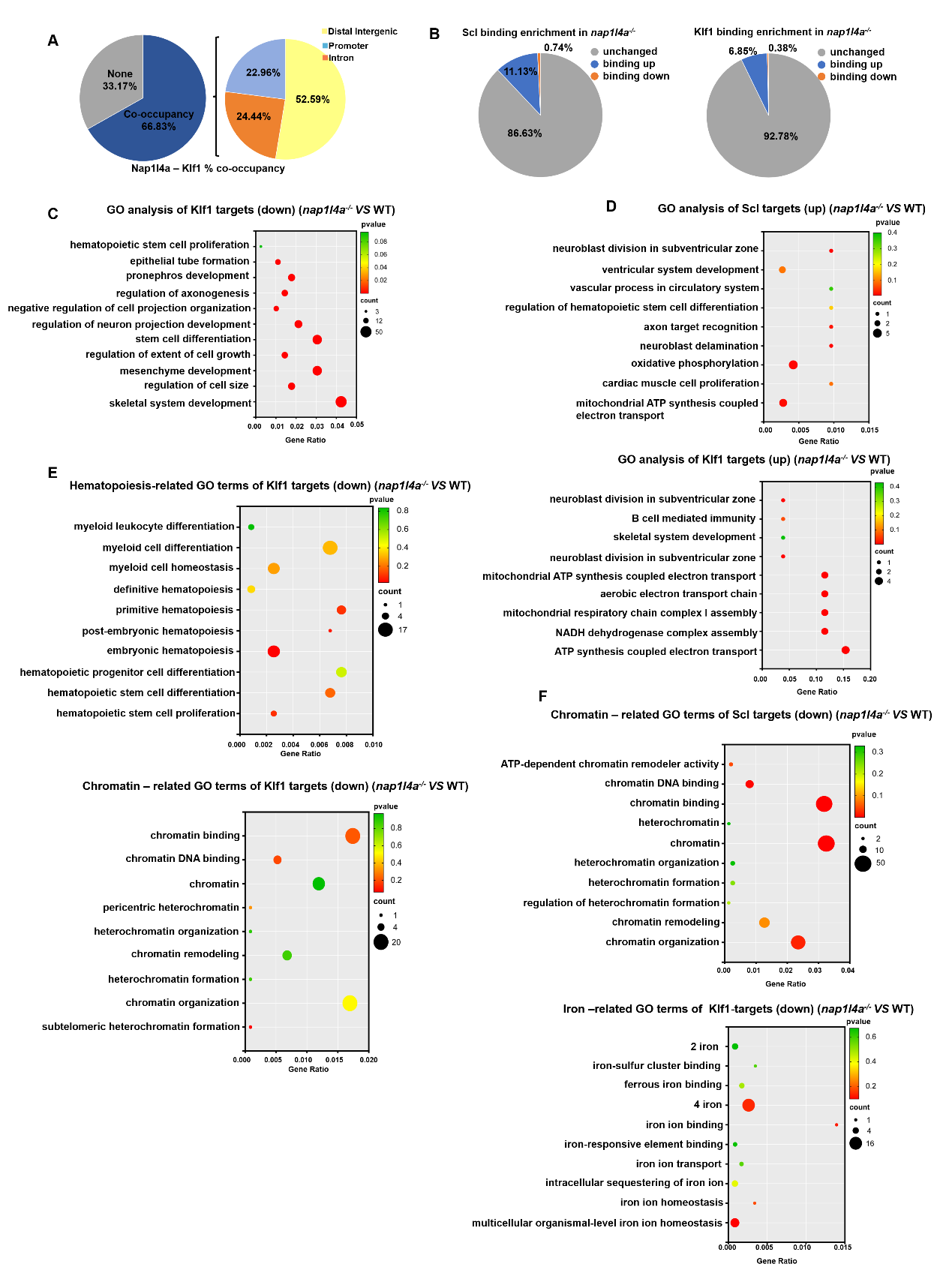


**Fig.S7 GO enriched bubble plots of Klf1 and Scl CUT&Tag targets integrating with transcriptome DEGs.** (**A**) Co-occupancy of Nap1l4a and Klf1 during zebrafish embryogenesis at 24 hpf, and the two proteins sharing 66.83% co-occupancy, with 52.69% co-occupancy loci distribute in distal intergenic. (**B**) Changes of the binding enrichment of Scl and Klf1 occupied sites on chromatin were analyzed in *nap1l4a*^-/-^ mutants. (**C**) Bubble plot showing GO terms of Klf1 targets with binding down in *nap1l4a*^-/-^ relative to WT embryos at 24 hpf. (**D**) Bubble plot showing GO terms of Scl and Klf1 targets, with binding up, in *nap1l4a*^-/-^ relative to WT embryos at 24 hpf. (**E**) Bubble plot showing hemopoiesis-related GO terms of Klf1 targets, with binding down, in *nap1l4a*^-/-^ relative to WT embryos at 24 hpf. (**G**) Bubble plot showing chromatin-related GO terms of Scl targets, with binding down, in *nap1l4a*^-/-^ relative to WT embryos at 24 hpf. GO and KEGG enrichment analyses using ClusterProfiler (v 4.8.1) R package.


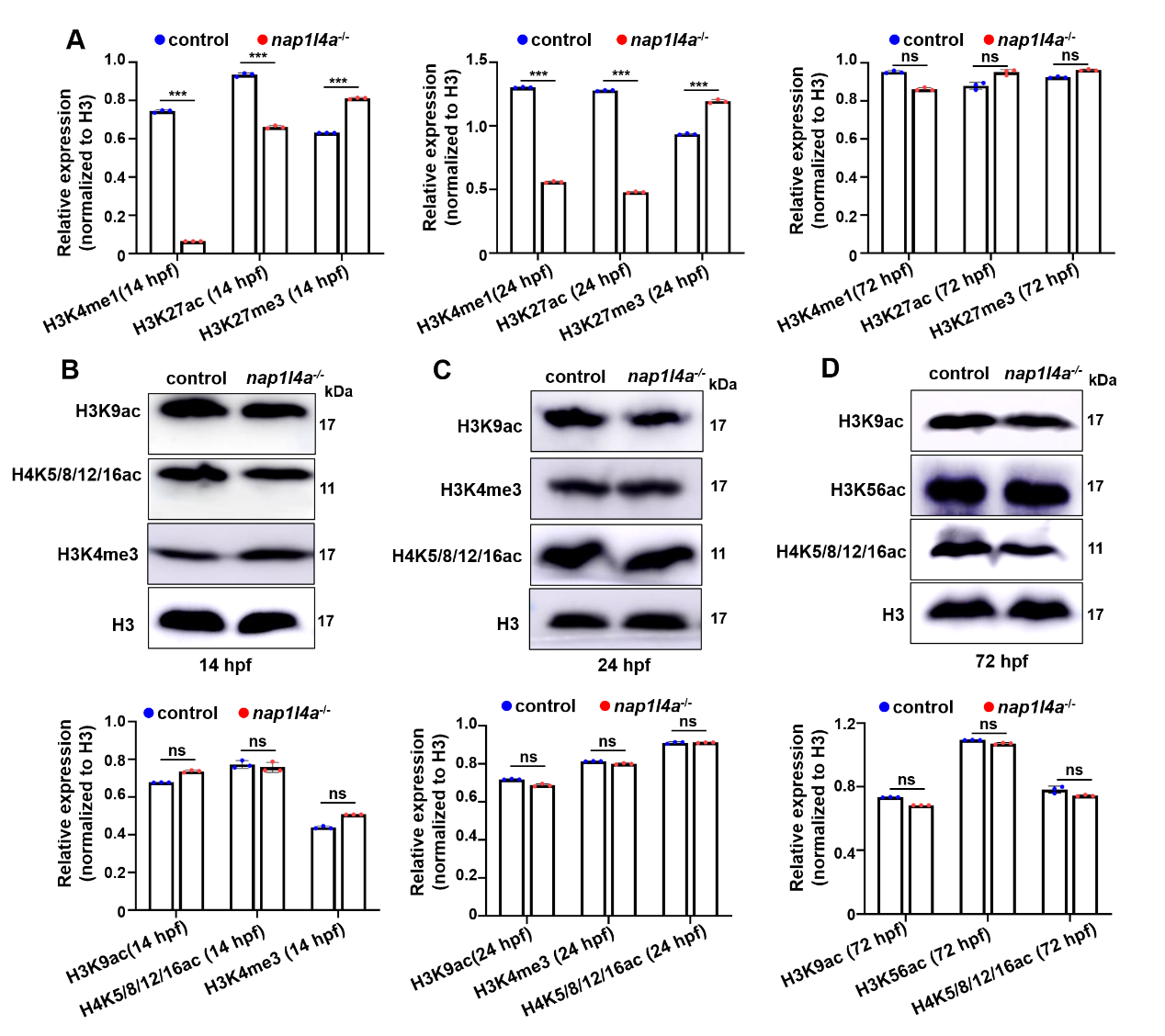


**Fig.S8 Effects of *nap1l4a* deficiency on epigenetic histone proteins**. (**A**) Graphs showing the relative level of proteins H3K4me1, H3K27ac, and H3K27me3 at 14 hpf (left), 24 hpf (middle), and 72 hpf (right) in *nap1l4a*^-/-^ and WT control group respectively. (**B-D**) Protein levels of H3K9ac, H3K5/8/12/16ac, H3K4me3, and H3K56ac in *nap1l4a*^-/-^ and WT control embryos at 14 hpf (**B**), 24 hpf (**C**), and 72 hpf (**D**), with graphs showing the relative level of proteins H3K9ac, H3K5/8/12/16ac, H3K4me3 (**B, C**) and proteins H3K9ac, H3K56ac, H3K5/8/12/16ac (**D**). Each experiment was repeated at least three times, and a representative result is shown. Signals from WB images were quantified with ImageJ. Data were analyzed by *t* test using GraphPad Prism 8.0. Data are presented as mean ± SD. ****P* < 0.001, NS, not significant.


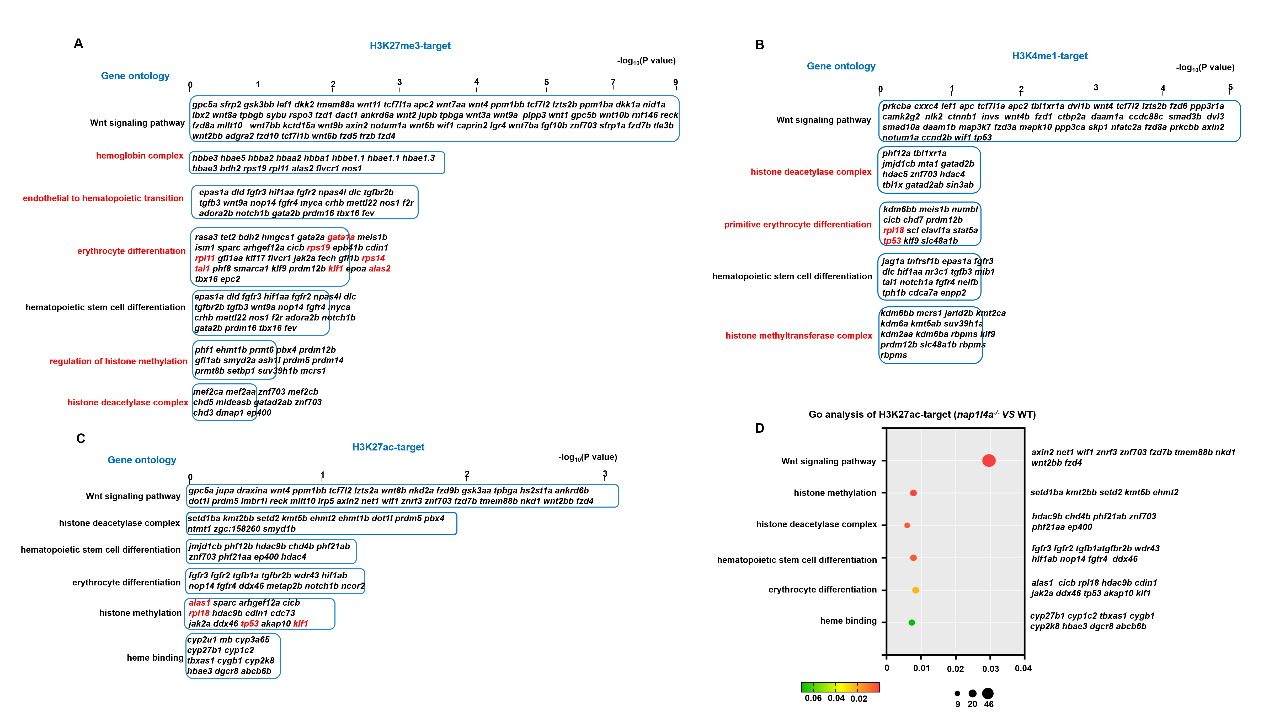


**Fig.S9 Effects of *nap1l4a* deficiency on histone epigenomes**. (**A**) GO terms of H3K27me3 targets with integration of the RNA-seq and CUT&Tag data, such as erythrocyte differentiation, regulation of histone methylation, histone deacetylase complex, and others. (**B**) GO terms of H3K4me1 targets with integration of the RNA-seq and CUT&Tag data, such as primitive erythrocyte differentiation, histone methytransferase complex, histone deacetylase complex, and others. (**C**) GO terms of H3K27ac targets with integration of the RNA-seq and CUT&Tag data, such as Wnt signaling pathway, erythrocyte differentiation, histone methylation, histone deacetylase complex, and others. (**D**) Bubble plot showing GO terms of H3K27ac targets in *nap1l4a*^-/-^ relative to WT control embryos at 24 hpf. GO and KEGG enrichment analyses using ClusterProfiler (v 4.8.1) R package.


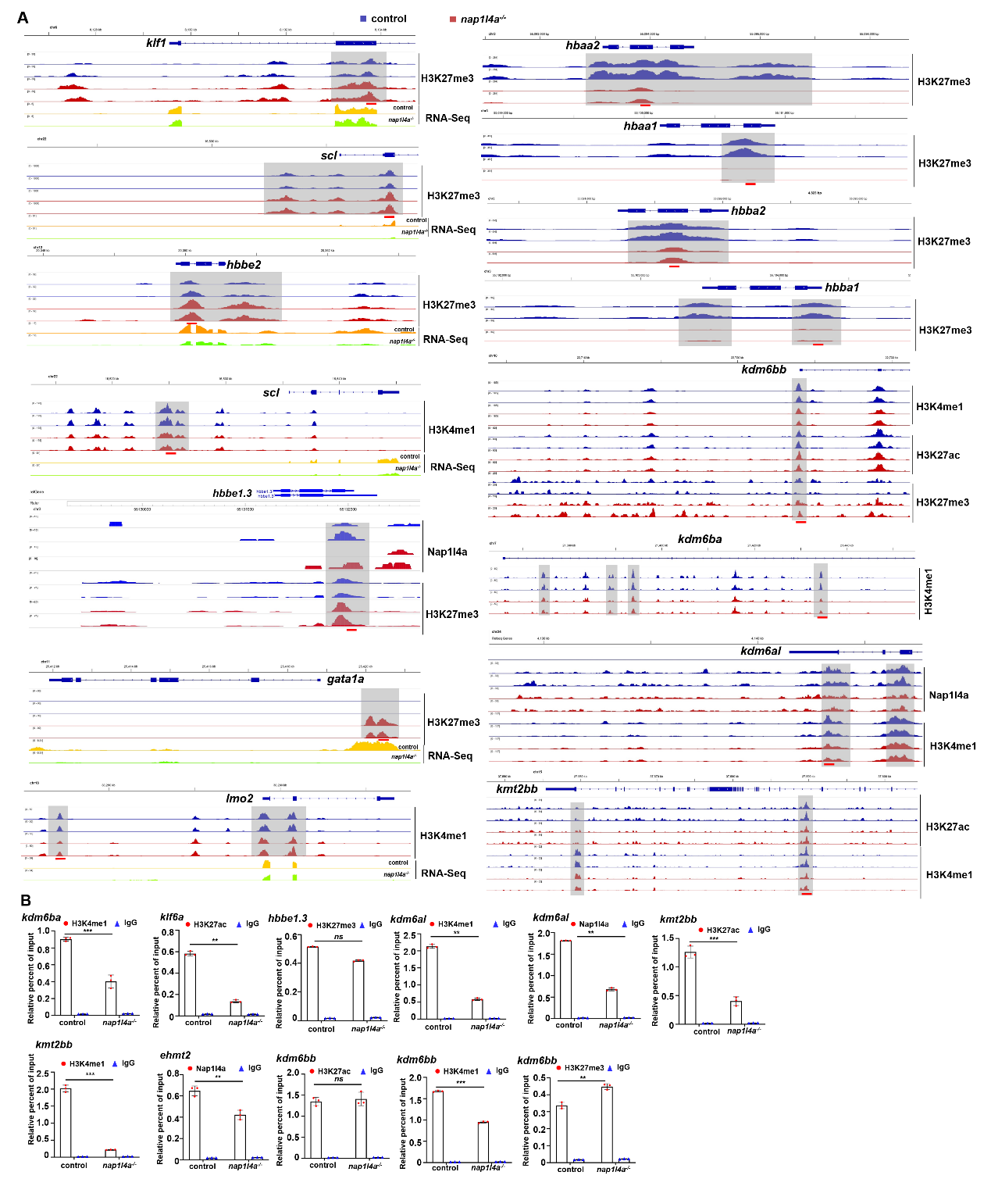


**Fig.S10 Effects of *nap1l4a* deficiency on histone epigenomes on erythropoiesis genes.** (**A**) IGV browser tracks showing binding profiles of H3K27me3, H3K4me1, H3K27ac, and Nap1l4a on genes *klf1*, *hbaa2*, *scl*, *hbaa1*, *hbba2*, *kdm6bb*, *hbbe1.3*, *kdm6ba*, *gata1a*, *kdm6al*, *lmo2* and *kmt2bb*, respectively. Gray-shaded regions highlight differential binding enrichment loci. (**B**) Enrichment of histone H3K27me3, H3K4me1, H3K27ac and Nap1l4a binding (by ChIP-qPCR) on promoter or enhancer regions of targeted genes in WT and *nap1l4a*^-/-^. Tested loci are underlined in red. Each experiment was repeated at least three times, and a representative result is shown. Data were analyzed by *t* test using GraphPad Prism 8.0. Data are presented as mean ± SD. ***P* < 0.01, ****P* < 0.001, NS, not significant.


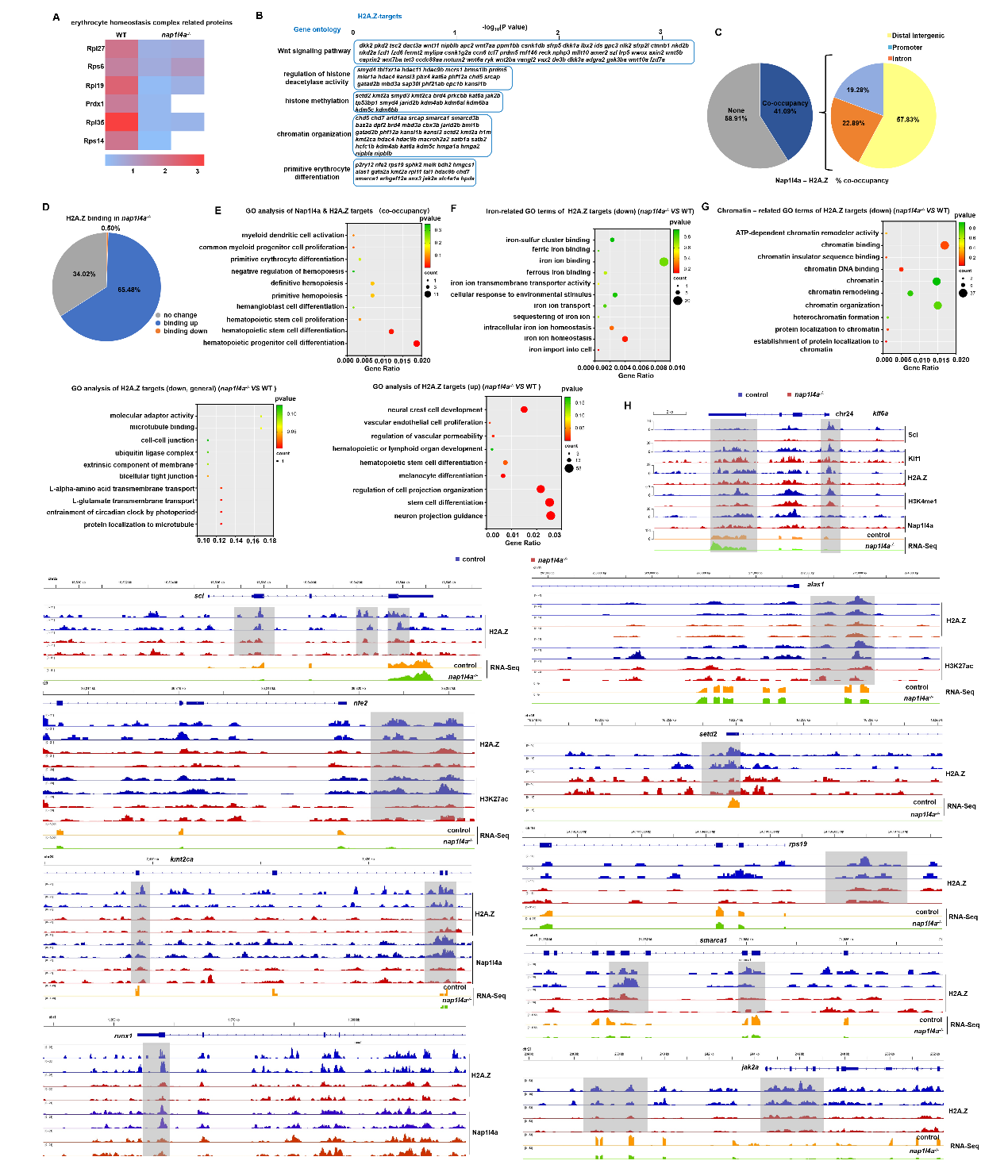


**Fig. S11 Effects of *nap1l4a* deficiency on H2A.Z chromatin profiles**. (**A**) Heatmaps of erythrocyte homeostasis complex related proteins (WT control *vs nap1l4a*^-/-^) identified by IP-MS using Nap1l4 antibody. (**B**) GO terms of H2A.Z targets with integration of the RNA-seq and CUT&Tag data. (**C**) Co-occupancy of Nap1l4a and H2A.Z during zebrafish embryogenesis at 24 hpf, and the two proteins sharing 41.09% co-occupancy, with 57.83% co-occupancy loci distribute in distal intergenic. (**D**) Changes of the binding enrichment of H2A.Z occupied loci on chromatin in *nap1l4a*^-/-^ mutants. (**E**) Bubble plot showing GO terms of Nap1l4a and H2A.Z co-occupancy targets. (**F**) Bubble plot showing chromatin-related, iron-related and general GO terms of H2A.Z targets, with binding down, in *nap1l4a*^-/-^ relative to WT control embryos at 24 hpf. (**G**) Bubble plot showing GO terms of H2A.Z targets, with binding up, in *nap1l4a*^-/-^ relative to WT control embryos at 24 hpf. (**H**) IGV browser tracks showing binding profiles of Scl, Klf1, H2A.Z, H3K4me1, H3K27ac, and Nap1l4a on genes *klf6a*, *kmt2ca*, *scl*, *setd2*, *alas1*, *rps19*, *nfe2*, *jak2a*, *runx1* and *smarca1*, respectively. Gray-shaded regions highlight differential binding enrichment loci. GO and KEGG enrichment analyses using ClusterProfiler (v 4.8.1) R package.


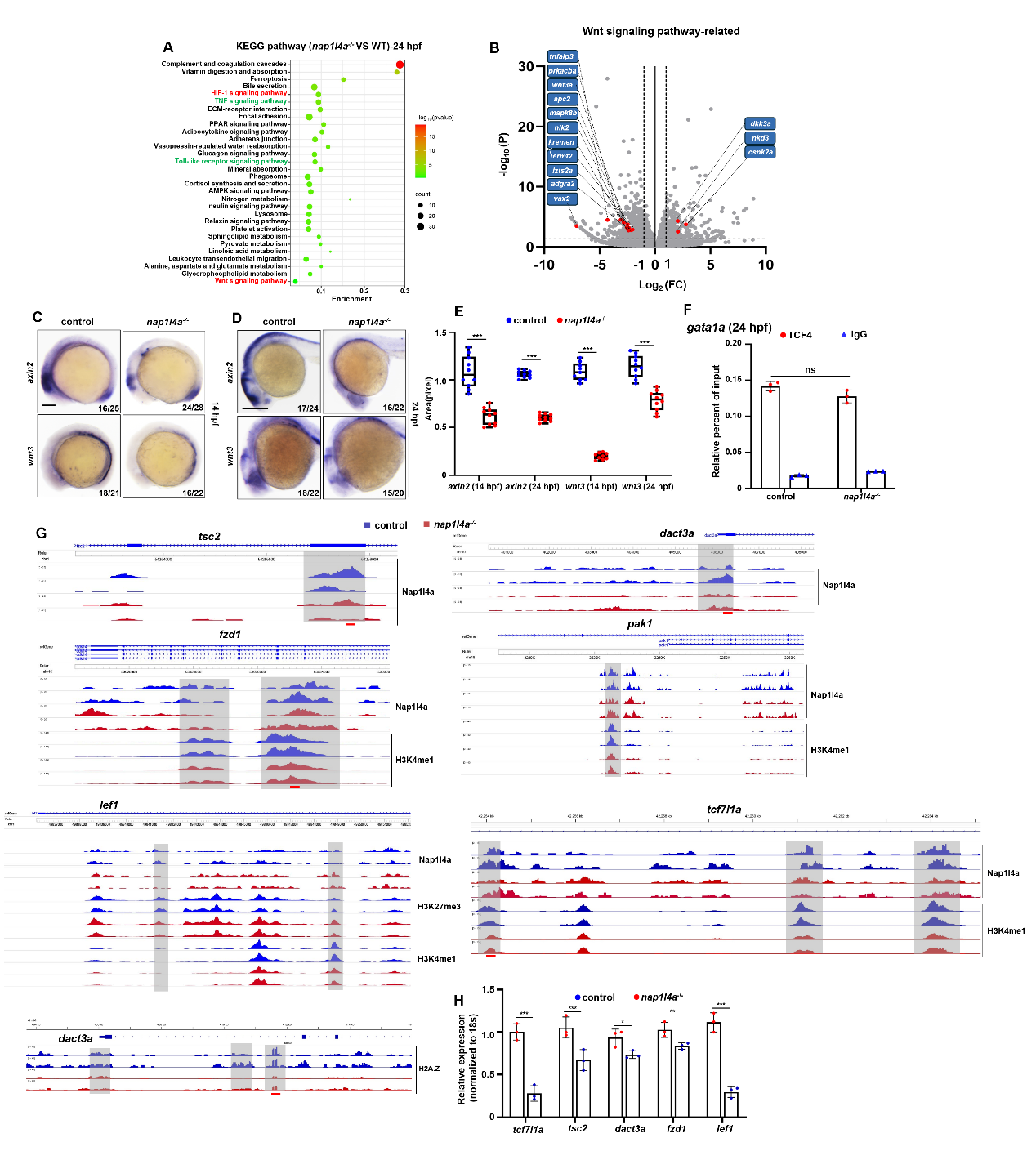


**Fig. S12 WNT/β-Catenin signaling pathway mediates *nap1l4a* effects on erythropoiesis**. (**A**) Bubble plot showing KEGG pathway in *nap1l4a*^-/-^ relative to WT control embryos at 24 hpf. (**B**) Volcano plot of WNT signaling pathway related DEGs between *nap1l4a*^-/-^ and WT control embryos at 24 hpf. (**C**-**E**) WISH analysis of *axin2* and *wnt3* in *nap1l4a*^-/-^ and WT control embryos at 14 hpf (**C**) and 24 hpf (**D**), with box and whisker plots showing relative transcriptional levels (**E**). (**F**) Enrichment of TCF4 binding (by ChIP - qPCR) on promoter or enhancer regions of targeted gene *gata1a* in WT control and *nap1l4a*^-/-^ embryos. (**G**) IGV browser tracks showing binding profiles of Nap1l4a, H3K4me1, H3K27me3 and H2A.Z on genes *tsc2*, *dact3a*, *fzd1*, *pak1*, *lef1*, t*cfal1a* and *dact3a*. Gray-shaded regions highlight differential binding enrichment loci. (**I**) qRT-PCR assays for Nap1l4a targets in WT control and *nap1l4a* mutant at 24 hpf. **C, D**. lateral view, anterior to the left. Each experiment was repeated at least three times, and a representative result is shown. TIGR Multi-Experiment Viewer (MeV) was used for showing volcano plots of DEGs. Signals from WISH images were quantified with ImageJ. Data were analyzed by *t* test using GraphPad Prism 8.0. Data are presented as mean ± SD. **P* < 0.05, ***P* < 0.01, ****P* < 0.001, NS, not significant. Scale bars: 75 μm in **C** and **D**.
