# Supplementary figures and images for: Nap1L4a Cooperates with Scl/Klf1 to Recruit H2A.Z in Mediating Interactions Among Cis‐Regulatory Elements and Transcription Required for Primitive Erythropoiesis in Zebrafish
Source: Adv Sci (Weinh). 2025 Dec 12;13(12):e13762. doi: 10.1002/advs.202513762 (PMC12948261; doi:10.1002/advs.202513762)

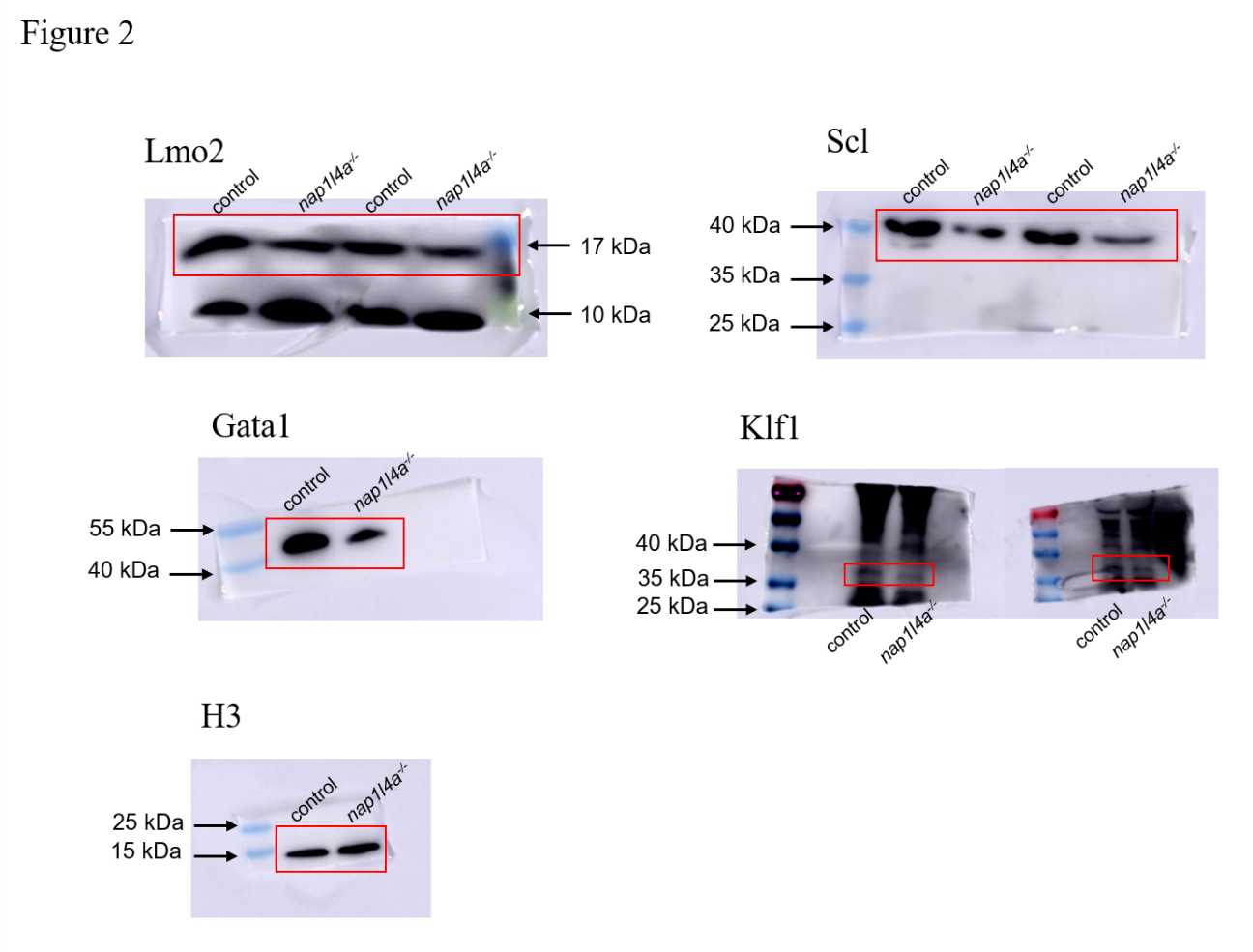


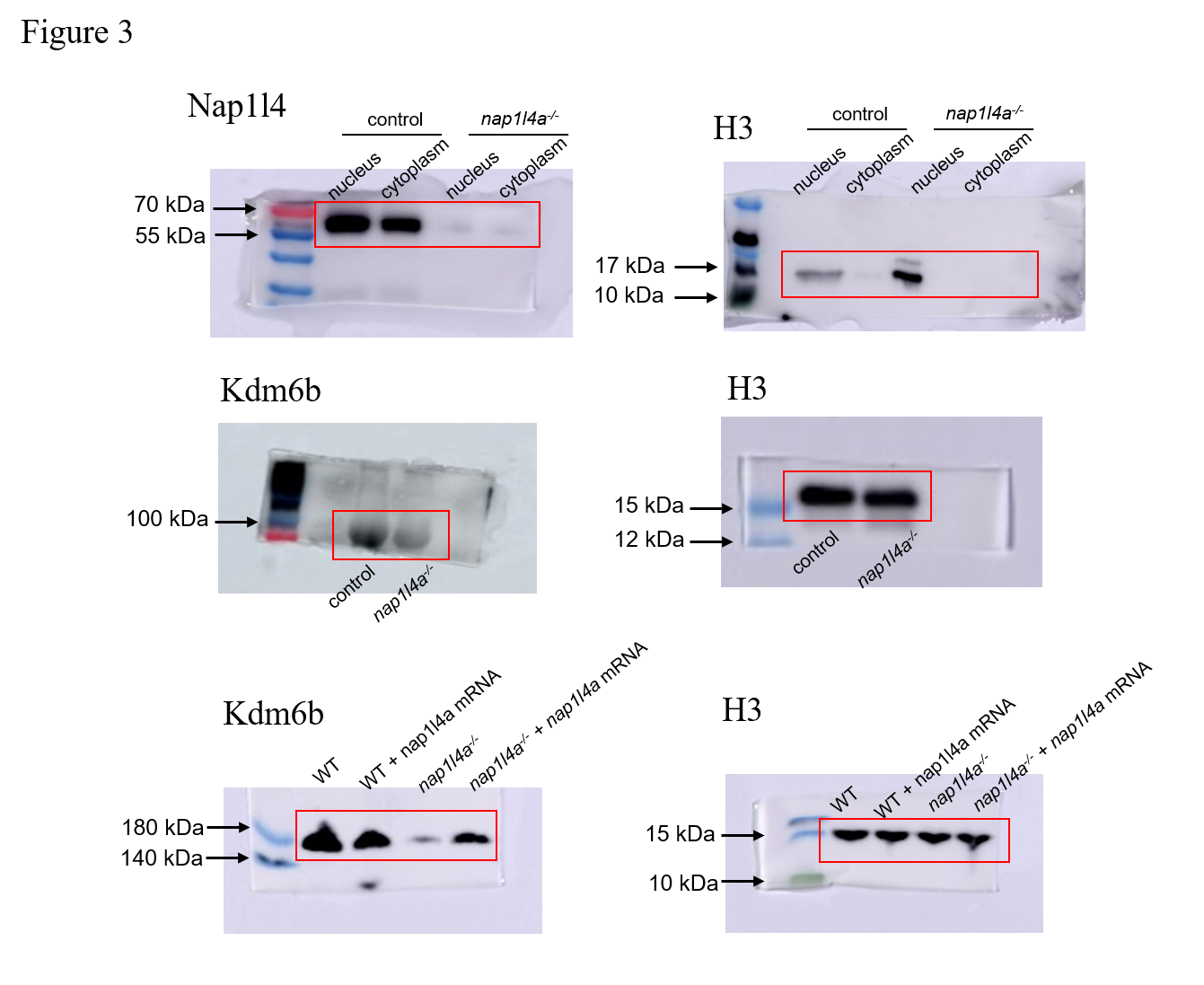


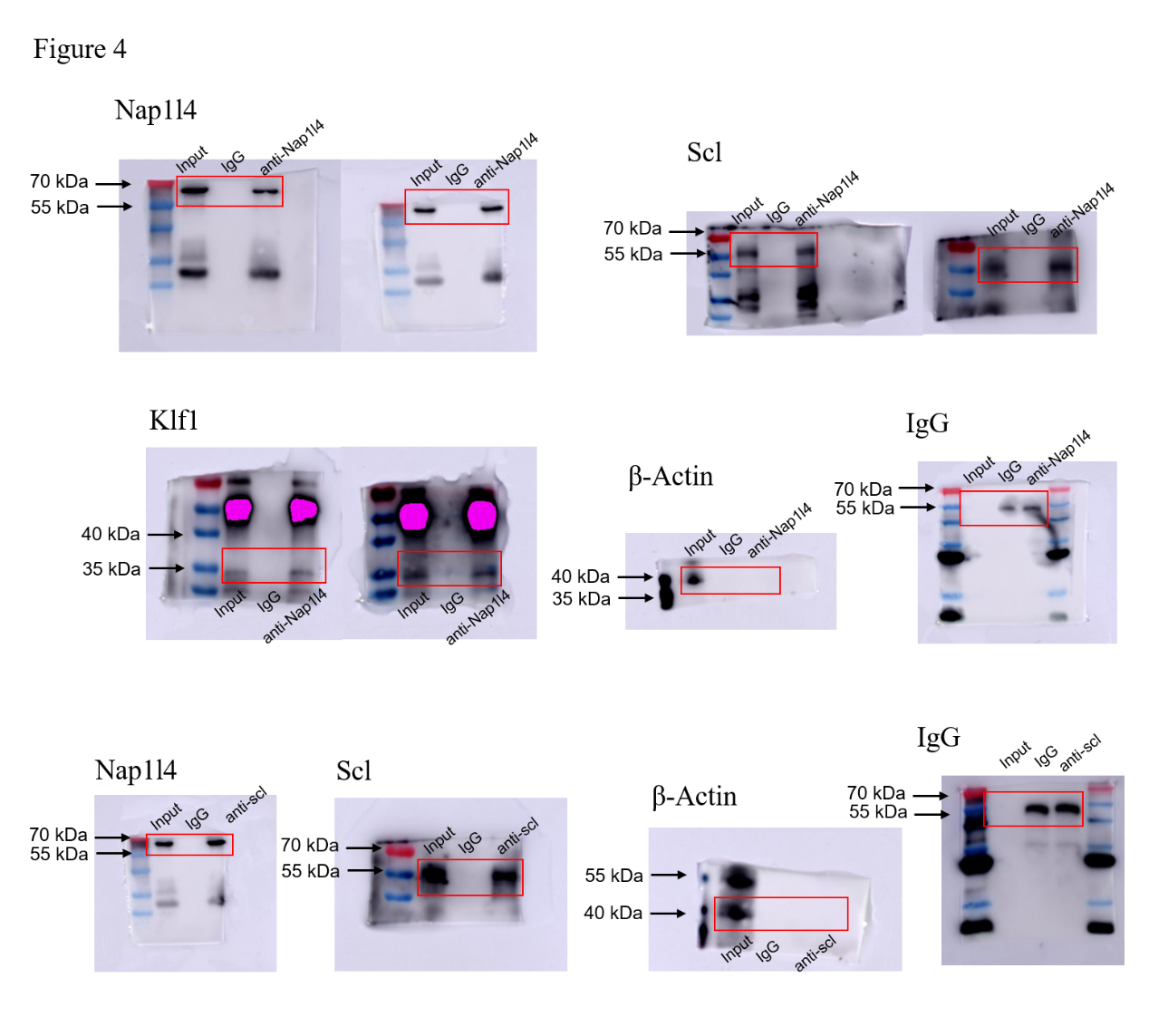

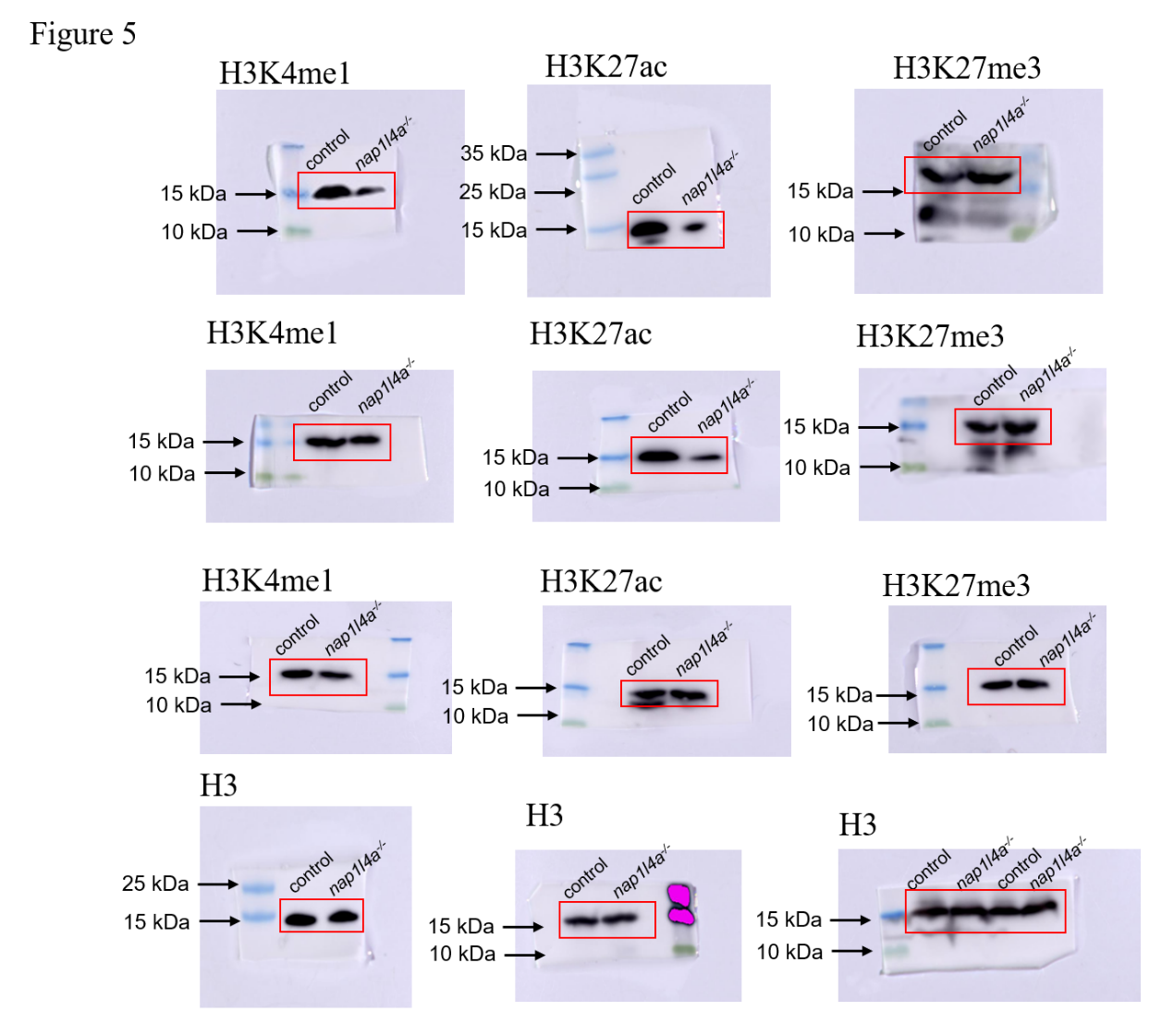

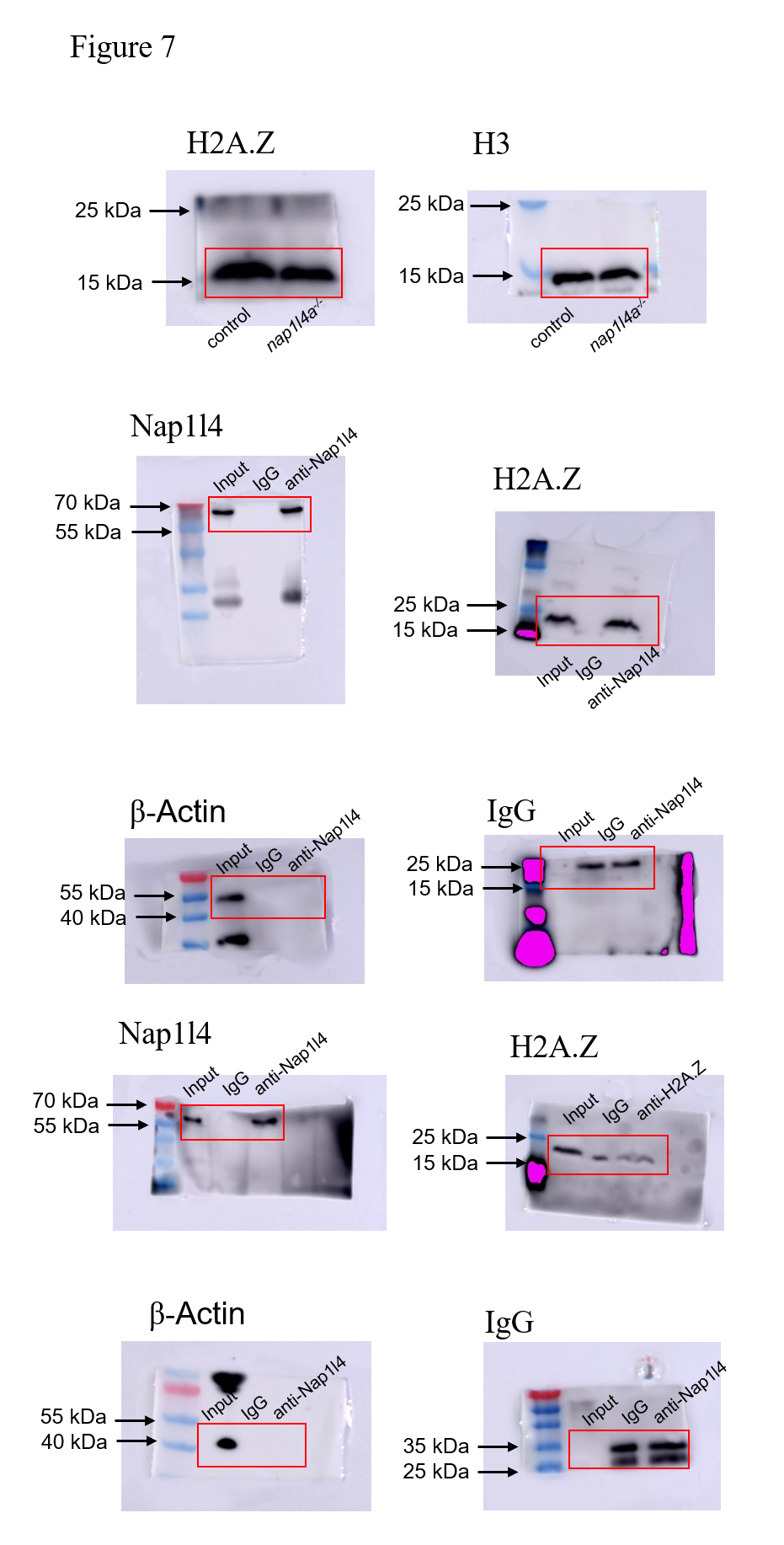

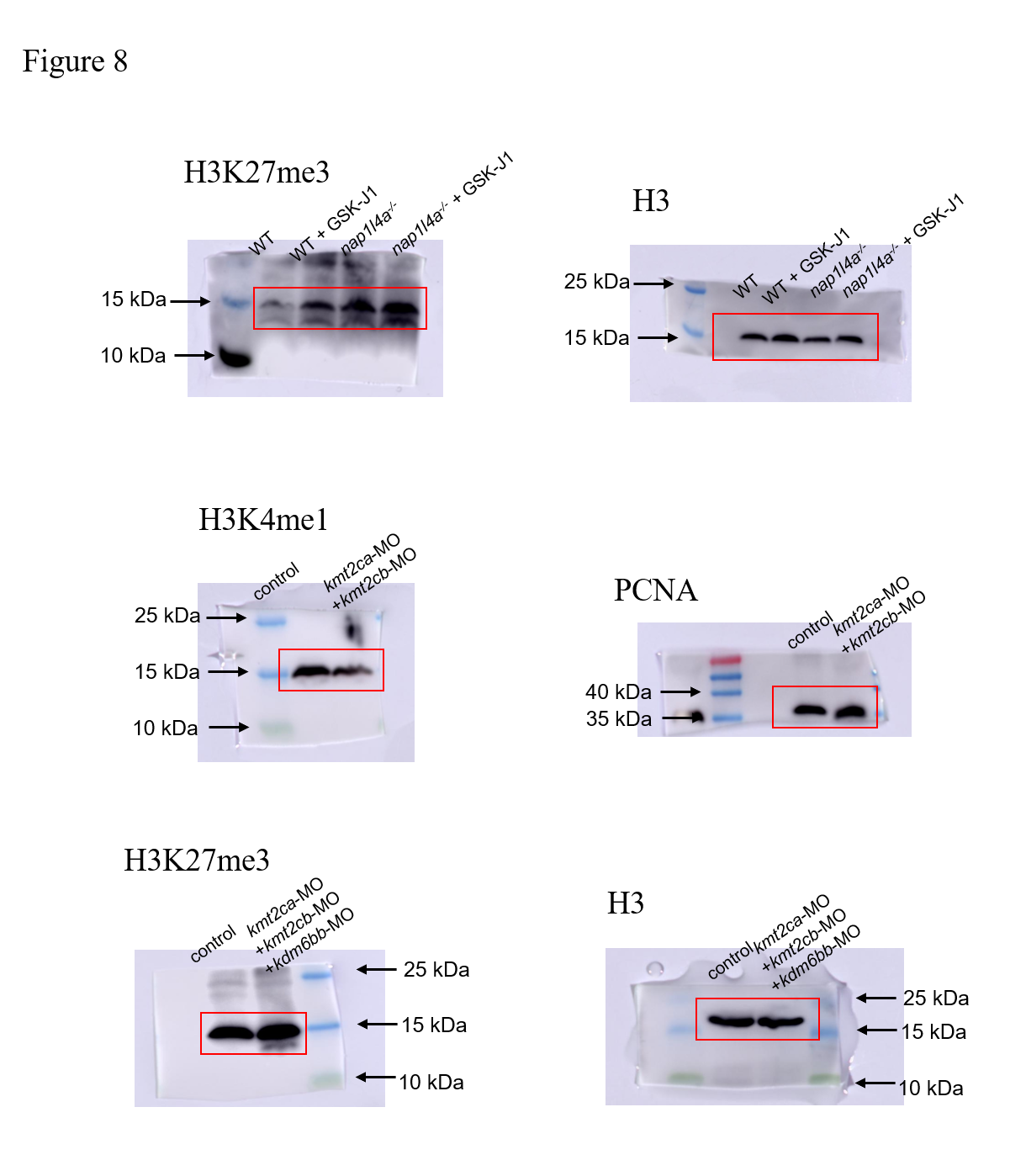


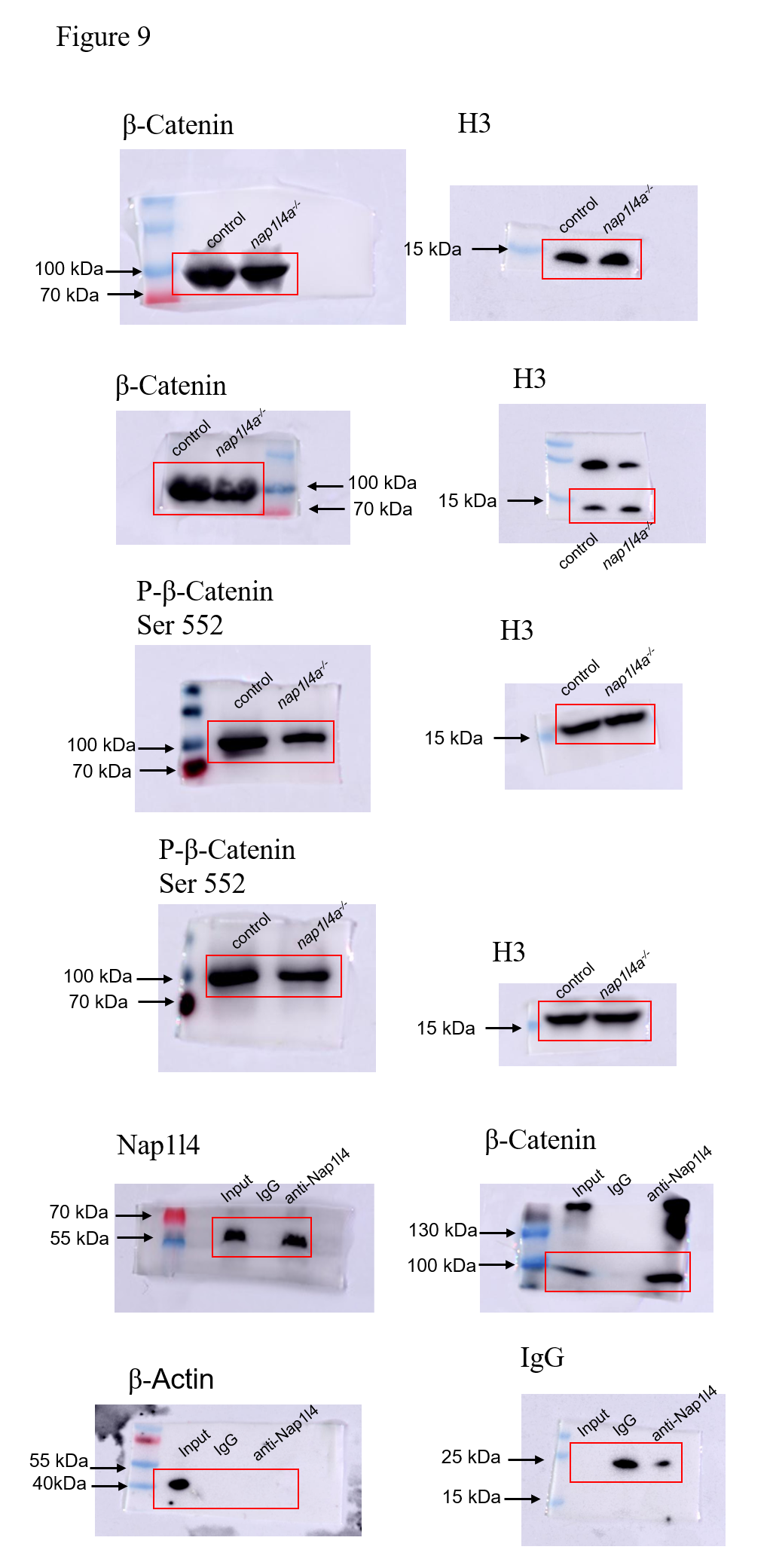

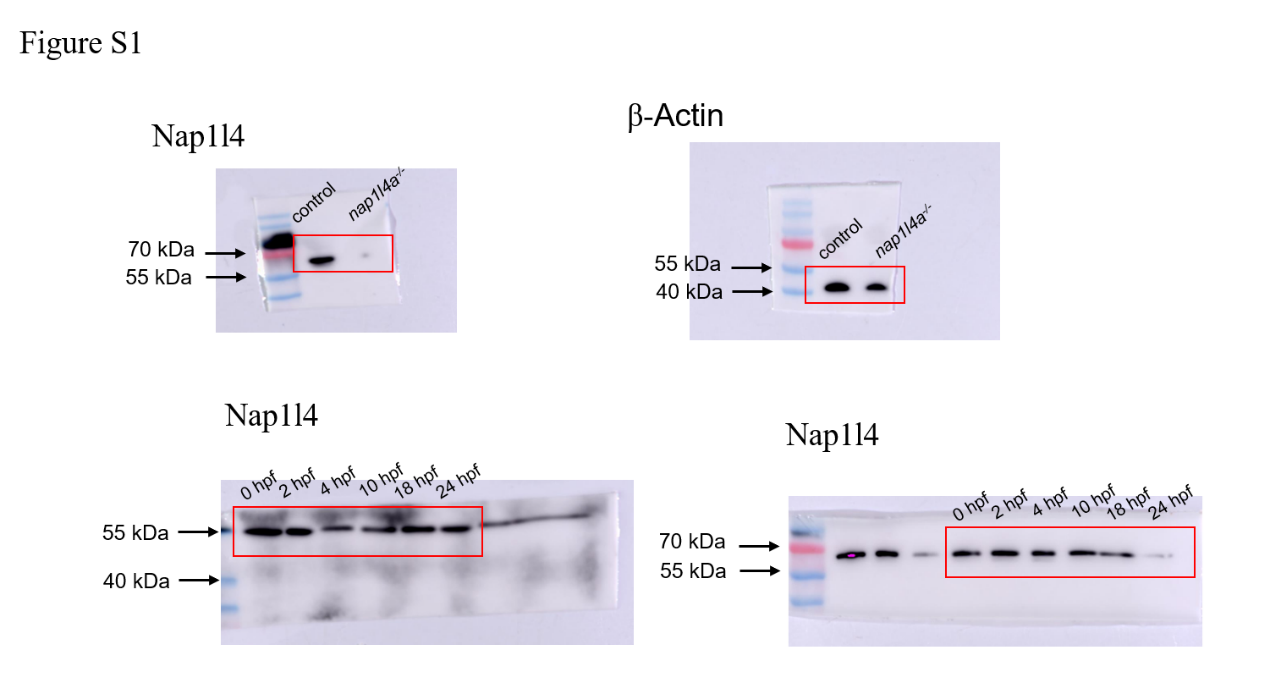

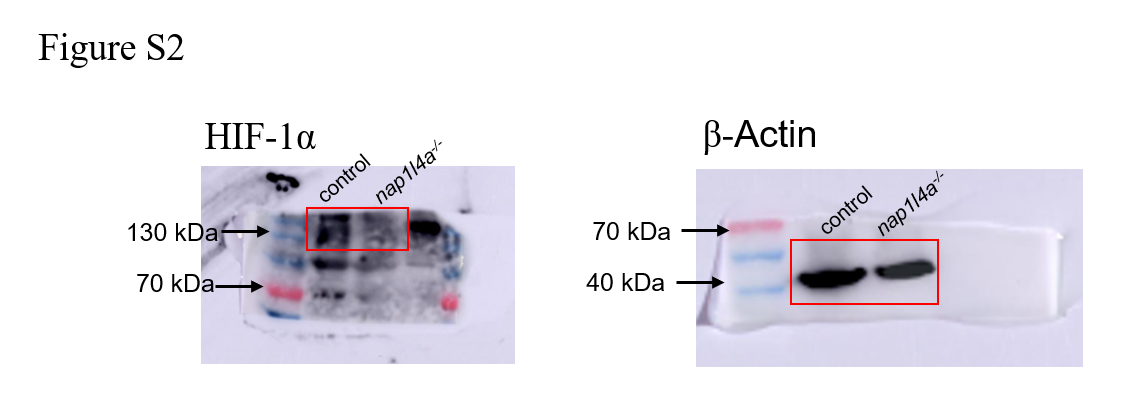

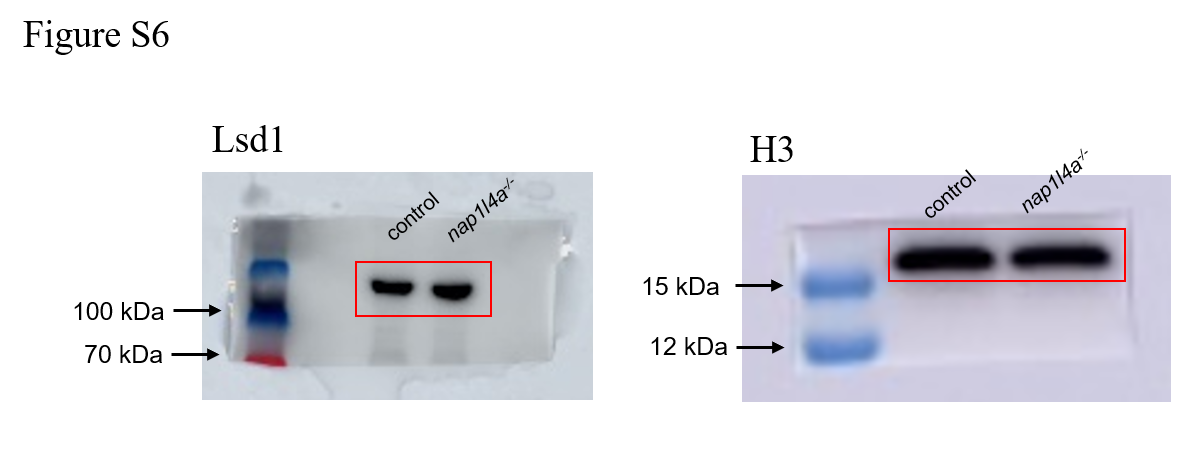

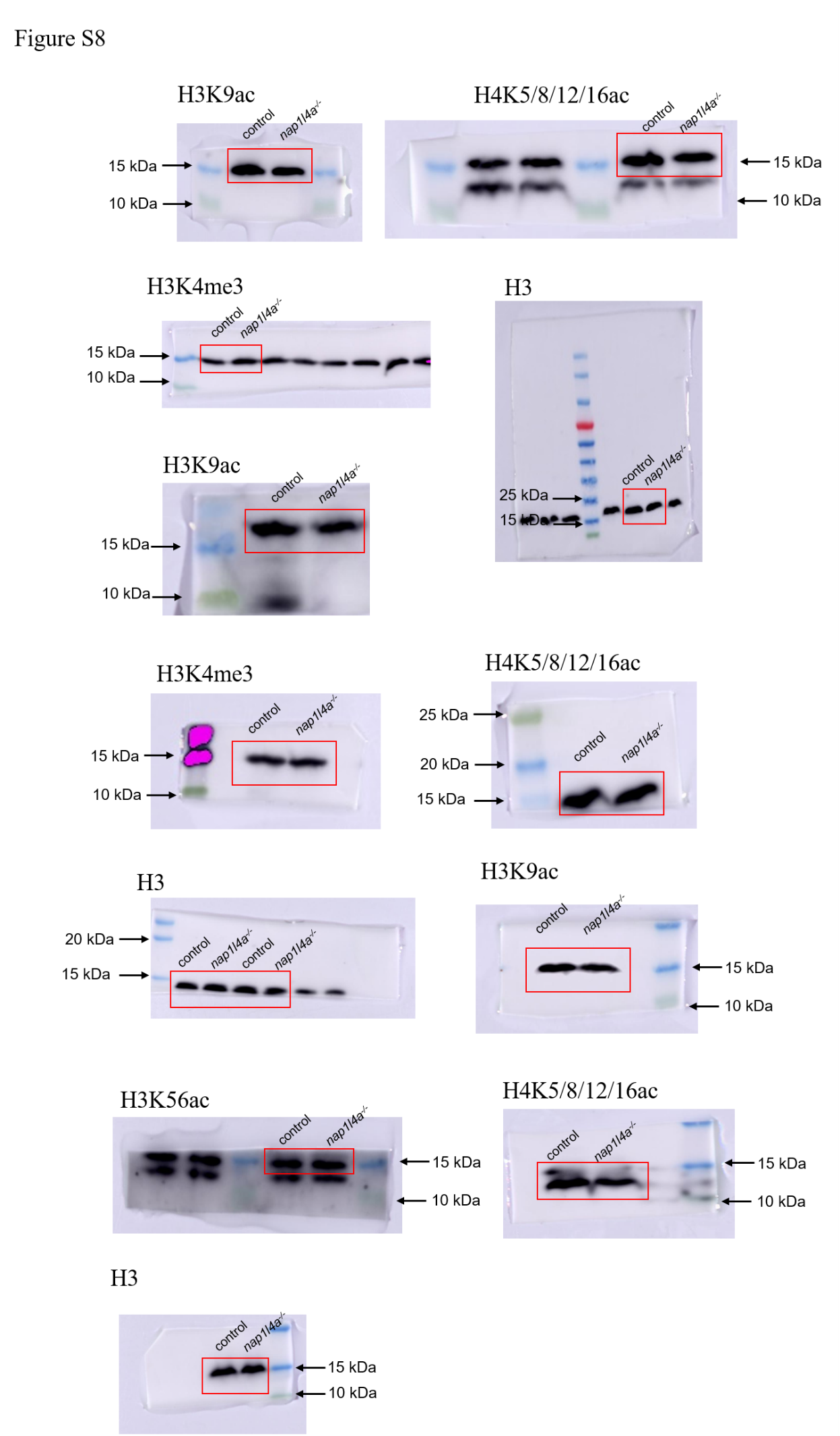

Supplement: Supplementary file 3 — Supporting Information [file ADVS-13-e13762-s003.xlsx]
